# Land Cover Constrains Range Shifts in Northern Iberian Bird Species Under Climate Change Scenarios

**DOI:** 10.1002/ece3.71863

**Published:** 2025-07-30

**Authors:** Laura Cardador, Irene Batlle, A. Márcia Barbosa, Neftalí Sillero, Luís Reino

**Affiliations:** ^1^ Departament de Biologia Evolutiva, Ecologia i Ciències Ambientals, Facultat de Biologia Universitat de Barcelona (UB) Barcelona Spain; ^2^ Institut de Recerca de la Biodiversitat (IRBio), Universitat de Barcelona (UB) Barcelona Spain; ^3^ Faculty of Sciences of the University of Porto CICGE, Research Centre on Geo‐Spatial Sciences Vila Nova de Gaia Portugal; ^4^ Faculty of Architecture, CEAU, Center for Studies in Architecture and Urbanism University of Porto Porto Portugal; ^5^ CIBIO, Centro de Investigação Em Biodiversidade e Recursos Genéticos InBIO Laboratório Associado, Campus de Vairão, Universidade do Porto Vairão Portugal; ^6^ BIOPOLIS Program in Genomics, Biodiversity and Land Planning CIBIO, Campus de Vairão Vairão Portugal; ^7^ CIBIO, Centro de Investigação Em Biodiversidade e Recursos Genéticos InBIO Laboratório Associado, Instituto Superior de Agronomia, Universidade de Lisboa, Tapada da Ajuda Lisboa Portugal

**Keywords:** avifauna, biogeography, distribution edges, ecological niche models, species distribution models, variation partitioning

## Abstract

Climate and land cover changes are considered some of the most important drivers of the current biodiversity crisis. The assessment of their combined impacts is starting to attract greater attention. In this study, we assess the role of land cover changes in constraining species range shifts under climate change scenarios in the Iberian Peninsula. We assessed the relative contribution of climate and land cover to the current distribution of 32 northern Iberian bird species using ecological niche models and deviance partitioning. We also assessed how different scenarios of climate change may affect species distributions, with or without changes in land cover. In addition to the independent effect of climate, the current distribution of northern Iberian birds was also shaped by the joint effects that can be indistinguishably attributed to climate or land cover (42% of the explained variations). When changes in climate and land cover were decoupled by allowing only one of them to change concerning current conditions, there was evidence of an effect of land cover in climate‐driven predictions: predicted range size shifts were significantly lower when changes in climate were not accompanied by changes in land cover. This highlights the importance of incorporating land cover management alongside climate adaptation strategies in conservation planning.

## Graphical abstract

This study examines how land cover changes influence species range shifts under climate change for 32 bird species in the Iberian Peninsula, highlighting the need to consider both factors in conservation planning.

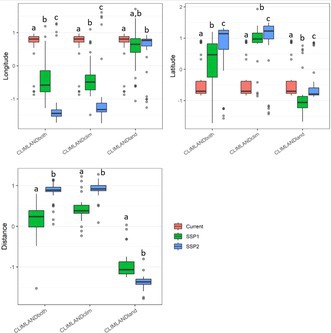

## 1 Introduction

Climate change is considered one of the major threats to biodiversity. The considerable increase in temperatures over the last century, as well as increases in the incidence and severity of droughts and extreme precipitation events (Karl and Trenberth 2003), jeopardise the environmental suitability within current species' distribution ranges. This environmental pressure has forced species to either adapt or shift their distributions towards more suitable areas beyond their previous range limits (Thomas et al. 2004; Enriquez‐Urzelai et al. 2019; Sillero et al. 2022).

To understand and predict species responses under future climate change scenarios, ecological niche models (ENMs, also termed species distribution models, SDMs) have become invaluable tools (Sillero et al. 2021). Correlative models (the most common form of ENMs) use information on current or historical species‐environment relationships (Araújo and Peterson 2012; Guisan and Thuiller 2005) for predicting species responses under future scenarios. Although most such models have focused on climate, a growing body of evidence shows that other factors, particularly land cover in terrestrial systems, play significant roles in shaping species distributions (Reino et al. 2018; Zamora‐Gutierrez et al. 2018; Cadieux et al. 2020; Labadie et al. 2024). Land cover refers to the natural (e.g., native forests, wetlands), semi‐natural (e.g., agricultural habitats) and artificial features (e.g., urban areas) present on the land. It determines the quantity and quality of resources and habitat conditions to which species are exposed, thereby influencing the physiological and resource limitations imposed by broad‐scale climate (e.g., Mantyka‐Pringle et al. 2014; Zamora‐Gutierrez et al. 2018).

Although the impact of climate and land cover variables on species distributions has been extensively studied in isolation, relatively few studies have examined the combined effects of climate and land cover heterogeneity, though this area has gained more attention in recent years (Barbet‐Massin et al. 2012; Princé et al. 2015; Cadieux et al. 2020; Labadie et al. 2024). This gap may stem from the complexity of simulating land cover changes realistically and/or the prevailing view that climate primarily drives species distributions at large scales, with land cover playing a secondary role in refining these patterns (Pearson et al. 2004). However, given that climate and land cover often co‐vary (Titeux et al. 2016), some of the variation attributed to climate may actually be explained by land cover, even at broad scales. In this case, neglecting changes in land cover during forecasting exercises at large scales could potentially lead to erroneous predictions, particularly if the current covariation between climate and land cover changes. This can also constrain effective management strategies (Mantyka‐Pringle et al. 2014; Titeux et al. 2016).

In this study, we aimed to discern the specific contributions of climate and land cover on bird species with a northern distribution in the Iberian Peninsula. Iberia is considered a biodiversity hotspot within the European continent (Gaston and David 1994), and it hosts a high number of bird species (Equipa Atlas 2008; Martí and Moral 2004). Unfortunately, as in many other European regions, habitat suitability for Iberian birds has decreased over time due to human‐induced environmental shifts (Arenas‐Castro and Sillero 2021), and there has been a declining trend in several bird species populations (Lehikoinen et al. 2019). Notably, bird species native to the northern region of the Peninsula are especially vulnerable to climate change, as this region marks the southern edge of their range, already near the limits of their tolerance (Triviño et al. 2013). Whether land cover changes may constrain the anticipated climate‐driven range shifts of northern species, as occurs with other Iberian species such as farmland birds (Reino et al. 2018), has been largely unexplored. Here, we hypothesise that both land cover and climate are relevant in shaping bird species distributions. We thus explored how future distributions of suitable areas for these species will be affected by: (1) predictions considering only climate or both climate and land cover changes, and (2) different climate change scenarios based on socioeconomic considerations.

## 2 Materials and Methods

### 2.1 Occurrence Data

Our study area is located in the Iberian Peninsula, with a specific focus on species that predominantly inhabit northern regions. These species are particularly vulnerable to the impacts of climate change (Triviño et al. 2013). Northern Iberian bird species are characterised by their presence across a wide portion of Central and Western Europe, reaching their southern limit in the Eurosiberian region of the Iberian Peninsula (Ramírez and Tellería 2003). Some of them also extend into the interior of the Peninsula, but only in mountainous terrain (Ramírez and Tellería 2003). In total, we identified 32 bird species in Iberia that fit these range characteristics, based on BirdLife International and NatureServe (2014) and on Spanish and Portuguese national bird atlases (Figure S1). To estimate the range of conditions suitable for each of these species in Iberia, we compiled occurrence data at a global scale (i.e., considering the global breeding distribution of each species). We did not use spatially restricted data on species distributions because it constrains the range of environmental conditions over which models are trained, and thus their applicability for predictive purposes (Thuiller, Brotons, et al. 2004; Titeux et al. 2017). We compiled global species occurrence data for model training from the citizen science platform ‘eBird Observation Dataset’ in the Global Biodiversity Information Facility (GBIF.org 2017; http://www.gbif.org, see Table S1 for a complete list of GBIF occurrence downloads), and selected data on the native breeding range according to BirdLife International and NatureServe (2014) range maps. We aggregated occurrence data at 5 arcminute resolution (~70 km^2^ at the study area). This resolution is considered appropriate to capture the main responses to both climate and land cover (Luoto et al. 2007) with acceptable computing time. We removed from the analyses samples with reported geospatial issues or known location uncertainty above 5‐arcminute resolution. A species is considered present at a 5‐arcminute pixel if at least one occurrence record exists. The mean number of 5‐arcminute occurrences at a global scale was 4814 per species (SD = 4089, range = 508–15,623). Additionally, to validate models conducted at a global scale in the Iberian Peninsula, we compiled information publicly available from the Inventario Español de Especies Terrestres from the Spanish Government (corresponding to the breeding bird atlas from Martí and Moral (2004)) and from the breeding bird atlas from Portugal (Equipa Atlas 2008) (Figure S1). We used the central grid coordinates as occurrences. The mean number of 5‐arcminute resolution occurrences was 470 (SD = 529, range = 13–1696, with 90% of species having > 50 occurrences available).

### 2.2 Environmental Layers

As descriptors of climatic variability, we considered four variables known to affect bird distributions (e.g., Cardador and Blackburn 2019): maximum temperature of the warmest month (*T*_max_), minimum temperature of the coldest month (*T*_min_), annual precipitation (Pan) and precipitation seasonality (coefficient of variation of monthly values, Pcv). The current climatic data were derived from monthly precipitation and temperature data from WORLDCLIM 2 (‘near current’ data, 1970–2000, Fick and Hijmans 2017) at 5‐arcminute resolution. To describe current land cover variability, we considered six land cover variables obtained from Chen et al. (2020), representing the percentage of needleleaf evergreen forests (including all subcategories), broadleaf deciduous forests (including all subcategories), grasslands (including ‘C3 Arctic’, ‘C3 Grass’ and ‘C4 Grass’ categories), shrubs (including ‘broadleaf evergreen shrub’ and ‘broadleaf deciduous shrub’ and their subcategories), crops (including ‘corn’, ‘wheat’, ‘soybean’, ‘cotton’, ‘rice’, ‘sugar crop’, ‘other crop’, ‘bioenergy crop’ and all their subcategories) and urban habitats. Land cover variables were available at https://doi.org/10.25584/data.2020‐07.1357/1644253 (Chen et al. 2020) at 3‐arcminute spatial resolution for the year 2015. We resampled them to a 5‐arcminute spatial resolution using the ‘bilinear’ method with the package ‘raster’ in R software. Prior to the analyses, a correlation matrix among all variables at a global scale was created. Variables were moderately correlated (Pearson correlation coefficient, absolute value, mean ± SD: 0.16 ± 0.14, range: 0.015–0.75) and thus all of them were considered for modelling.

The future climate and land cover variables were obtained from the same sources and using the same methodology as for current data, and they corresponded to two different shared socio‐economic and representative concentration pathways (SSP‐RCP), SSP2‐4.5 and SSP1‐2.6, for the year 2055. The year 2055 or similar decadal approaches was chosen as a mid‐century benchmark, commonly used in ecological forecasting to assess medium‐term impacts of land cover and climate change (e.g., Newbold et al. 2015; Titeux et al. 2017). It also aligns with the standard time horizon in climate scenarios (e.g., CMIP5/6), enabling comparability across studies and policy relevance for planning and mitigation. Future land cover was projected accounting for varying climate conditions (Chen et al. 2020). The SSP2‐4.5 scenario assumes current socioeconomic and technological trends are maintained (Riahi et al. 2017) and greenhouse emissions are intermediate among those potential (Chen et al. 2020). The SSP1‐2.6 scenario describes a more optimistic view, where the world is expected to shift towards a more environmentally sustainable development and greenhouse gas emissions are the lowest among those plausible according to Chen et al. (2020).

Each scenario was obtained with two different general circulation models (GCM) representing different sensitivities to regional versus global climate processes (hereafter referred to as IPSL and MIROC, respectively). This was done to assess the robustness of scenario comparisons to GCM choice. The GCMs selected from climate and land cover were the most similar between them in relation to all the available options (IPSL‐CM6A‐LR and MIROC6 for climate and IPSL‐CM5A‐LR and MIROC5 for land cover). As the results from the two GCMs were broadly consistent, we present only the IPSL‐based scenarios in the main text for simplicity.

All scenarios considered lead to increases in minimum and maximum temperatures and decreases in annual precipitation in Iberia (Table 1 and Table S2). In general, scenario SSP2‐4.5 projected slightly higher increases in temperature than SSP1‐2.6 (Table 1 and Table S2). For precipitation, scenario SSP2‐4.5 projected slightly higher decreases than SSP1‐2.6. Regarding land cover, the SSP1‐2.6 scenario projected an increase in needleleaf and broadleaf forests and crops and a reduction in natural open habitats (grasslands and shrubs) (Table 1 and Table S2). The SSP2‐4.5 scenario projected more similar land cover to the current conditions.

**TABLE 1:** Climate and land cover characteristics of socio‐economic scenarios (SSP1‐2.6, SSP2‐4.5) based on general circulation model IPSL and current conditions in Iberia.

Variables	Current	SSP1‐2.6	SSP2‐4.5
**Climate**			
*T*_min_ (°C)	1.6 ± 2.9 (−10.0–9.3)	3.0 ± 2.7 (−8.1–10.5)	3.8 ± 2.7 (−7.2–11.1)
*T*_max_ (°C)	29.5 ± 3.6 (17.7–36.5)	32.7 ± 3.7 (21.5–40.2)	34.2 ± 3.7 (23.2–41.3)
Pan (mm)	674 ± 321 (224–1925)	622 ± 304 (194–1831)	593 ± 292 (193–1753)
Pcv	0.43 ± 0.14 (0.15–0.79)	0.44 ± 0.13 (0.18–0.79)	0.44 ± 0.13 (0.18–0.78)
**Land cover types**			
Needleleaf (%)	7.9 ± 17.7 (0–100)	13.7 ± 25.2 (0–100)	8.6 ± 18.8 (0–100)
Broadleaf (%)	2.5 ± 6.1 (0–75)	4.3 ± 10.6 (0–100)	2.7 ± 6.6 (0–84)
Grasslands (%)	40.9 ± 33.3 (0–100)	33.0 ± 33.9 (0–100)	38.3 ± 33.1 (0–100)
Shrubs (%)	21.2 ± 28.2 (0–100)	16.3 ± 27.0 (0–100)	19.6 ± 27.3 (0–100)
Crops (%)	25.5 ± 27.2 (0–100)	30.7 ± 31.5 (0–100)	28.8 ± 29.0 (0–100)
Urban (%)	1.2 ± 3.4 (0–78)	1.2 ± 3.4 (0–78)	1.2 ± 3.4 (0–78)

*Note:* Mean ± SD and range (in parenthesis) of, minimum temperature of the coldest month (*T*_min_), maximum temperature of the warmest month (*T*_max_), annual precipitation (Pan), precipitation seasonality (coefficient of variation of monthly values, Pcv) and percentage of different land cover types at 5‐arcminute resolution are shown.

### 2.3 Sampling Bias

To account for spatial sampling biases in the data due to potential variations in birding effort, a sampling bias variable was created to be included as a predictor in all models. This variable was computed by retrieving occurrence data at the family level for each species from GBIF and generated as a kernel density map at a 5‐arcminute resolution (Elith et al. 2010; Gallardo and Aldridge 2013), using ArcMap 10.5. Species occurrence data from the same taxonomic family are expected to suffer from the same detection limitations, so this controls for the effect of spatial sampling biases and detection limitations in observed distribution patterns.

### 2.4 Factors Affecting Current Species Distributions

We conducted deviance partitioning analyses to estimate the independent contribution of climate and land cover variables on current global species distributions of the 32 species, as well as joint effects that cannot be unambiguously attributed to one variable set or another due to spatial collinearity (Heikkinen et al. 2005). Deviance partitioning entails the calculation of incremental improvement in model fit due to the inclusion of a variable set in every possible model incorporating that variable set. For these calculations, we conducted partial generalised linear models (GLM) for each species, with binomial error distribution and logit‐link function, using all combinations of the two variable sets (i.e., climate, land cover, and climate + land cover) as predictors, and global occurrences of each species as the response variable. Both linear and quadratic effects of the variables were considered. Sampling bias was included as a control variable in all models. We ran models with a single set of a maximum of 10,000 pseudo‐absences randomly drawn from all biomes occupied by each species across its native breeding range (Cardador et al. 2017; Strubbe et al. 2015). Biomes occupied by each species were defined using maps available from Olson et al. (2001). We applied a stepwise approach based on Akaike's information criterion to avoid model overfitting. We built null GLMs with species occurrence as the response variable and sampling bias as the only predictor for comparison during deviance partitioning.

### 2.5 Ecological Niche Models

#### 2.5.1 Model Training

We fitted ENMs on the global occurrences of each species to generate future predictions of species' potential suitable areas in the Iberian Peninsula. Models were derived from an ensemble of three modelling techniques—GLM, MAXENT and Random Forest—using the R package biomod2 (Araújo et al. 2005; Thuiller et al. 2012; Zhu and Peterson 2017) to address variation related to particular modelling approaches (Araújo et al. 2005; Zhu and Peterson 2017). Pseudoabsences were selected using the same methodology as for GLM in deviance partitioning analyses (Section 2.4). ENMs were conducted considering both climate and land cover variables in both its linear and quadratic forms (CLIMLAND). To reduce the potential effect of sampling biases, we also included the sampling bias variable as a predictor in ENM models. Presences and pseudoabsences were weighted to ensure a neutral (0.5) prevalence. For each species, ensemble models were generated by averaging the predictions of all models computed.

#### 2.5.2 Model Accuracy

We evaluated model accuracy at both the global scale (with global occurrence data) and in Iberia (with the occurrence data from Portugal and Spain), using the Boyce index, a calibration metric which ranges from −1 to 1, with higher positive values indicating a stronger match between predictions and observations, and negative values indicating counter‐predictions (Boyce et al. 2002), implemented in the package ‘ecospat’ in R (Broenniman et al. 2014). We also calculated the area under the receiver operating characteristic curve (ROC), or AUC, a discrimination metric which ranges from 0 to 1, with values of 0.5 representing random predictions, values smaller than 0.5 representing counter predictions, and values closer to 1 representing better predictions (Phillips et al. 2006), using the package ‘pROC’ in R (Robin et al. 2011). Finally, we computed the true skill statistic (TSS), a discrimination metric which equals sensitivity+specificity‐1 (Allouche et al. 2006), using a conservative threshold where all pixels with predicted suitability above the 5% of occurrence values were considered suitable. We also computed relative variable importance in the final CLIMLAND models in the Iberian Peninsula, using the function ‘variables_importance’ in the ‘biomod2’ package.

#### 2.5.3 Model Predictions

We used the CLIMLAND models to estimate environmental suitability under future climate and land cover scenarios in Iberia at a 5‐arcminute resolution. Future scenarios corresponded to SSP2‐4.5 and SSP1‐2.6 for 2055 (see Section 2.2). Sampling bias was set to its maximum value for model predictions. We made three types of predictions to separate the role of climate and land cover changes: (a) CLIMLAND_both_ predictions consider both future climate and land cover values according to future scenarios; (b) CLIMLAND_clim_ considers only climate values according to the same scenarios, while keeping land cover values fixed at present; and (c) CLIMLAND_land_ considers only future land cover according to the future scenarios, while keeping climate variables fixed at present.

### 2.6 Area of Habitat and Range Centre According to ENM Predictions

We calculated the area of habitat (also known as extent of suitable habitat) in km^2^ for each species according to the binary map projections of different models computed, using the package ‘raster’ in R (Hijmans 2020). The threshold for the binary map projections was the predicted suitability above the 5% occurrence values in northern Iberia. We also estimated the range centroids for the distributions of suitable habitats, as mean values of the longitude and latitude of grid cells in Iberia weighted by predicted suitability. The predicted suitability refers to the continuous values obtained from ENM predictions. For each species, we calculated the distance between current and future range centroids as a measure of potential shift in the distribution of suitable areas. We evaluated differences in the area of habitat and the centroids' longitude, latitude and distance to current range centroid between different model predictions and scenarios using paired Wilcoxon signed‐rank tests. Since species distributions may be constrained to current locations due to smaller‐scale factors such as dispersal limitations, biotic interactions or microhabitat conditions, we repeated all calculations restricting the analysis to grid cells currently occupied by the species.

## 3 Results

### 3.1 Factors Affecting Current Species Distributions

The total explained deviance in the global distribution patterns of the study species by the two variable sets (i.e., climate + land cover) was 34% ± 9% (range: 17%–58%, *N* = 32). Deviance partitioning analyses showed that climate accounted for a large fraction of the total explained deviance (mean ± SD, 47% ± 11%, *N* = 32), closely followed by the joint effect of climate and land cover (42% ± 14%). The independent contribution of land cover was low (11% ± 8%). Results were highly consistent across species (Figure S2).

### 3.2 Ecological Niche Model Accuracy

Global model predictions showed a good agreement with species occurrences at a global scale (CLIMLAND: Boyce = 0.97 ± 0.04, AUC = 0.99 ± 0.01, TSS = 0.88 ± 0.06, *N* = 32, see individual values for each species in Table S3). Local model predictions also showed a good agreement with species occurrences derived from atlases in Iberia (CLIMLAND: Boyce = 0.94 ± 0.09, AUC = 0.94 ± 0.05, TSS = 0.74 ± 0.13, *N* = 32, Table S3). According to CLIMLAND, maximum temperature was the environmental variable with the highest relevance in Iberia, followed by minimum temperature and precipitation (Figure 1 and Figure S3, see also partial response curves for each species in Figures S4–S35).

**FIGURE 1:**
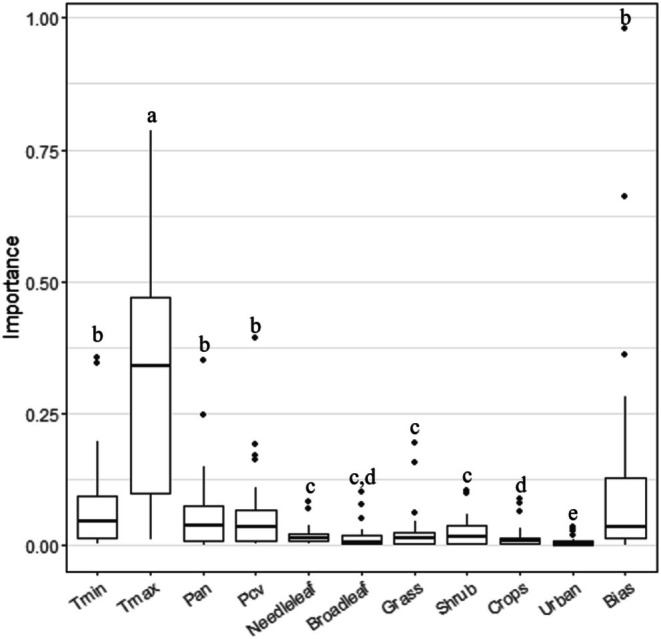
Importance of climate, land cover variables and bias in species distribution models of the study species within Iberia. Different letters indicate variables among which significant differences in importance were found. *T*_max_, maximum temperature of the warmest month; *T*_min_, minimum temperature of the coldest month; Pan, annual precipitation; Pcv, precipitation seasonality; needleleaf, needleleaf evergreen forests; broadleaf, broadleaf deciduous forests; bias, sampling bias variable.

### 3.3 Area of Habitat According to ENM Predictions

**FIGURE 2:**
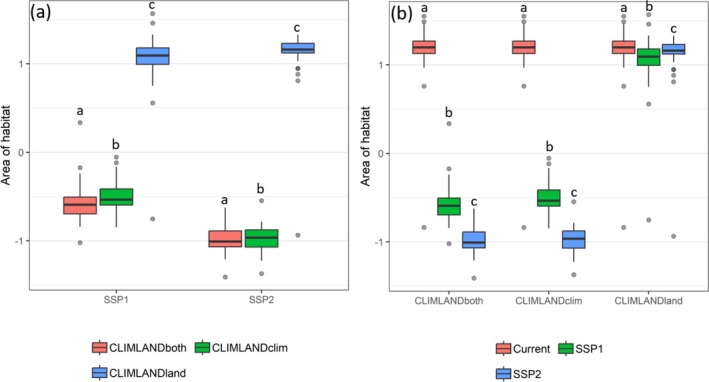
Comparison of the area of habitat according to (a) different types of model predictions (CLIMLAND_both_, CLIMLAND_clim_, CLIMLAND_land_) within a given scenario and (b) scenarios (Current, SSP1‐2.6 and SSP2‐4.5 with IPSL) within a given type of prediction. For graphical representation, standardised values of area of habitat for each species are used. Different letters indicate significant differences in pairwise comparisons of the area of suitable habitat. *N* = 32.

The predicted area of habitat was slightly higher when climate changes were not accompanied by land cover changes in future predictions (Wilcoxon signed‐rank test pair‐wise comparison between CLIMLAND_both_ and CLIMLAND_clim_ predictions, *P* range for different scenarios: < 0.001–0.04) and, particularly, when land cover changes were not accompanied by climate changes (Wilcoxon signed‐rank test pair‐wise comparison between CLIMLAND_both_ and CLIMLAND_land_, all *p* < 0.001, Figure 2a and Table S4).

**FIGURE 3:**
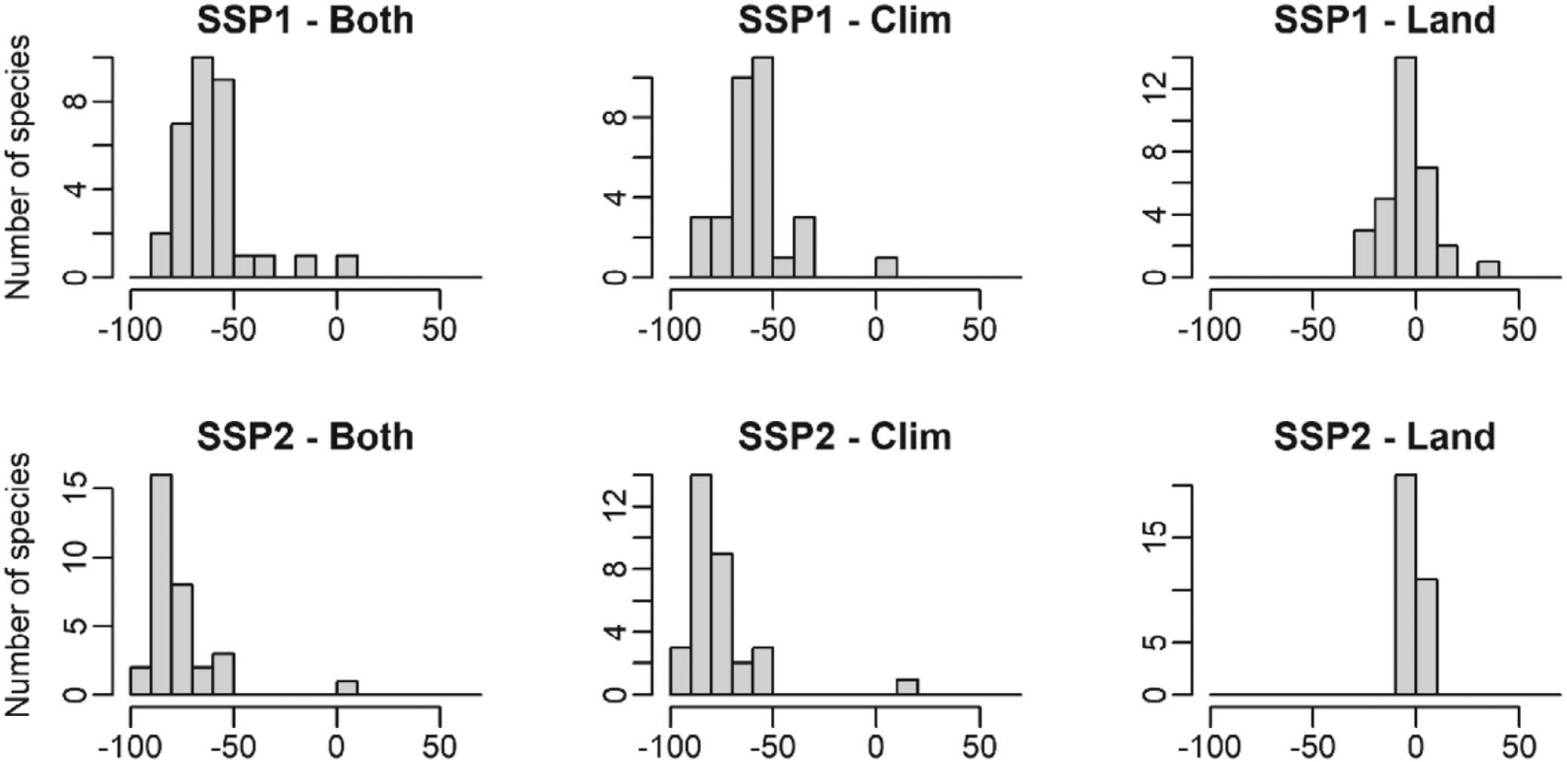
Frequency distribution of predicted shifts in the area of habitat for the studied species under different scenarios when both climate and land cover variables (Both), only climate (Clim) or only land cover (Land) are assumed to change within CLIMLAND model predictions. Shifts are expressed as the percentage change. *N* = 32.

When compared to current conditions, all CLIMLAND_both_, CLIMLAND_clim_ and CLIMLAND_land_ model predictions resulted in significant decreases in the area of habitat in Iberia for all scenarios (all *P* < 0.01, Figure 2b, Table S5). According to CLIMLAND_both_ and CLIMLAND_clim_, most species experienced reductions of 50%–100% in the area of habitat with respect to current conditions (Figure 3). Although significant, the differences in the area of habitat were smaller and more variable across species according to CLIMLAND_land_ predictions (Figure 3). For CLIMLAND_land_, the area of habitat decreased for between 62.5% and 81% of species across scenarios, with reductions > 5% observed for between 18% and 40% of species. Meanwhile, between 19% and 37.5% of species showed positive changes, with increases > 5% observed for between 9% and 13% of species (Figure 3 and Figure S36).

Pairwise comparisons of predicted area of habitat according to CLIMLAND_both_, CLIMLAND_clim_ or CLIMLAND_land_ model predictions across different future scenarios resulted in significant differences in most cases (Figure 2b and Table S5, but no significant differences according to the CLIMLAND_land_ predictions using MIROC was detected, *p* = 0.068, Table S5). Similar results arose when computation was restricted to grid cells currently occupied by the species (Table S5). Scenario SSP2‐4.5 produced higher decreases than SSP1‐2.6 according to CLIMLAND_both_ and CLIMLAND_clim_ models (Figure 2b).

### 3.4 Range Center According to ENM Predictions

**FIGURE 4:**
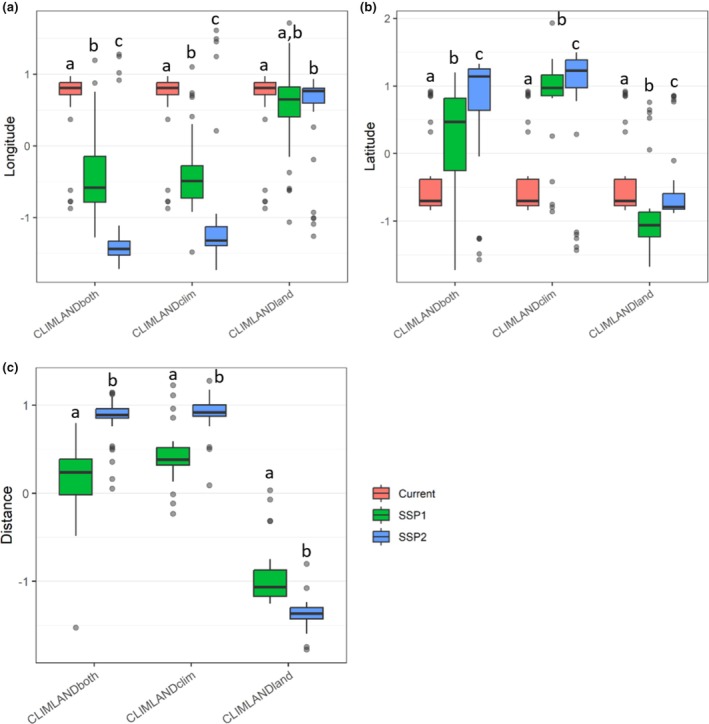
Comparison of the predicted centroid position of suitable habitat according to (a) longitude, (b) latitude and (c) distance to centroid of current suitable conditions among scenarios (Current, SSP1‐2.6 and SSP2‐4.5 with IPSL). For graphical representation, standardised longitude, latitude and distance values for each species are used. Results for different model predictions are shown. Different letters indicate significant differences in pairwise comparisons between different scenarios within a given type of model prediction. *N* = 32.

When compared to current conditions, all CLIMLAND_both_, CLIMLAND_clim_ and CLIMLAND_land_ model predictions resulted in significant shifts in latitudinal centroid position for all scenarios (Wilcoxon signed‐rank test pair‐wise comparisons, *p*‐range: < 0.001–0.03, Figure 4 and Table S6). Significant shifts in longitudinal centroid position with respect to present were also observed for all scenarios according to CLIMLAND_both_ and CLIMLAND_clim_ predictions (all *p* < 0.001, Table S6), as well as for scenario SSP2‐4.5 for CLIMLAND_land_ predictions (*p* < 0.001, Figure 4 and Table S6). Specifically, species would experience a centroid shift to the northeast according to CLIMLAND_both_ and CLIMLAND_clim_ predictions (Figure 4 and Figure S37). According to CLIMLAND_land_ predictions, shifts were southward under the SSP1‐2.6 scenario and south‐eastward according to SSP2‐4.5 (Figure 4 and Figure S37).

Differences in the distance between current and future range centroids were also observed across different model predictions. Under all scenarios, CLIMLAND_land_ projected a shorter distance between current and future range centroids compared to CLIMLAND_both_ and CLIMLAND_clim_ (all *p* < 0.001, Figure 5 and Table S7). Additionally, under the SSP1‐2.6 scenario, CLIMLAND_both_ predicted a shorter distance than CLIMLAND_clim_ (Figure 5). The distance of predicted position shifts by 2055 significantly differed among scenarios, regardless of the variable set expected to change (all *p* < 0.001, Table S6, Figure 4 and Figures S38–S70, similar results were obtained when area was restricted to current presences, Table S8). According to CLIMLAND_both_ and CLIMLAND_clim_ predictions, distances of shifts were longer under SSP2‐4.5 compared to SSP1‐2.6 (Figure 4).

**FIGURE 5:**
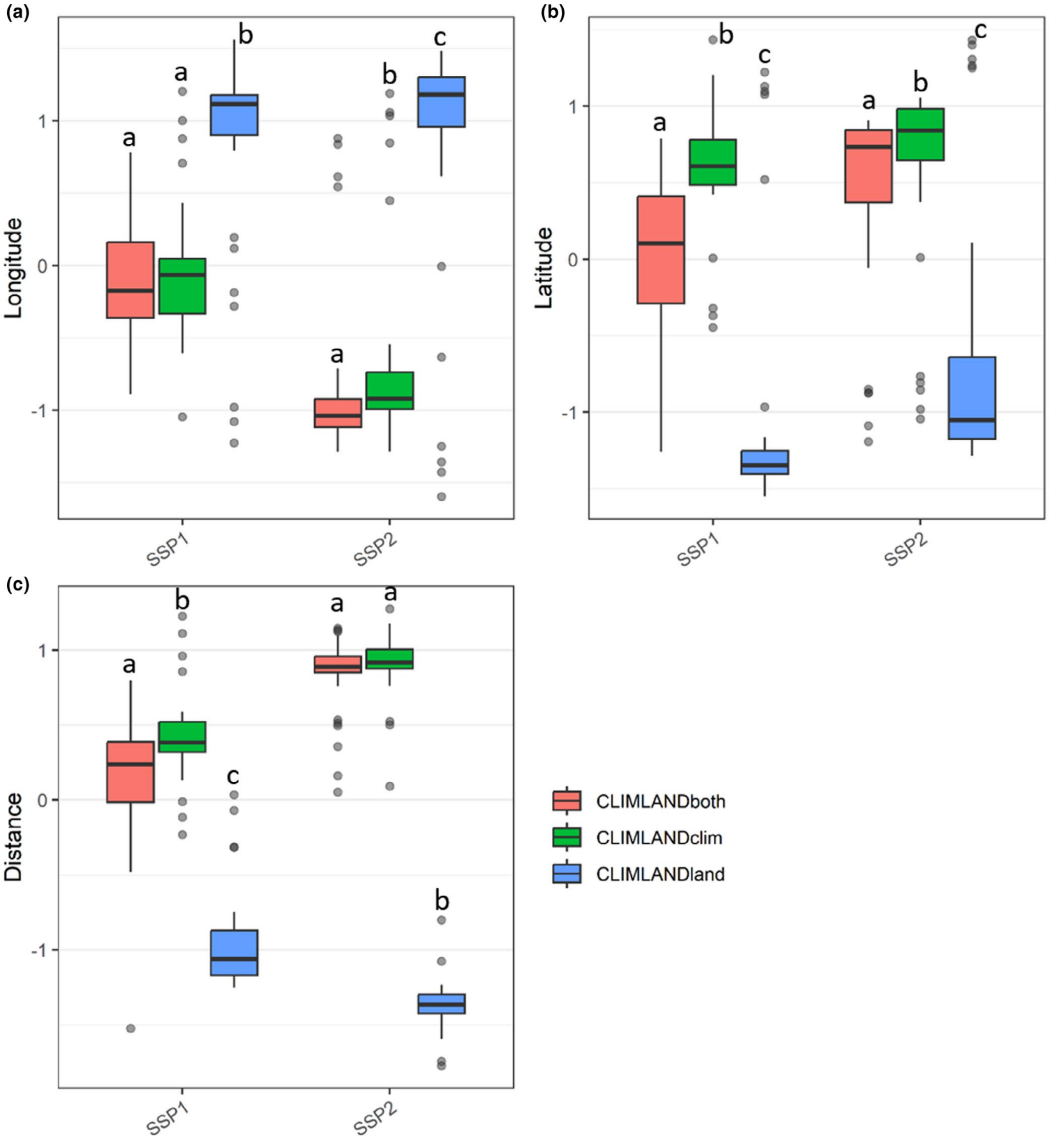
Comparison of the predicted centroid position of suitable habitat according to (a) longitude, (b) latitude, and (c) distance to centroid of current suitable conditions among model predictions (CLIMLAND_both_, CLIMLAND_clim_, CLIMLAND_land_). For graphical representation, standardised longitude, latitude and distance values for each species are used. Results for different future scenarios are shown. Different letters indicate significant differences in pairwise comparisons between different models within a given scenario. *N* = 32.

## 4 Discussion

This study underpins the relevance of both the independent effect of climate and its joint effect with land cover in shaping the distributions of northern Iberian birds. These results are in partial agreement with previous studies highlighting the role of climate as a predictor of species ranges (Thuiller, Araújo, and Lavorel 2004; Kuussaari et al. 2007), particularly at broad spatial scales (Luoto et al. 2007). However, the high influence of the joint effect of climate and land cover on the current distribution of the studied species showed that an important part of the explained variation in shaping current species distributions (42% on average) can be indistinguishably attributed to climate or land cover.

The fact that, for most species, only the joint effect of land cover and climate is relevant, and not the independent effect of land cover, could be linked to the coarse spatial resolution of our work, as the effects of climate and land cover drivers can be scale‐dependent (Regos et al. 2018). The 5‐arcminute resolution may be, for example, too coarse to capture critical microhabitat conditions, such as forest understories, rock crevices, alpine meadows or tree hollows to which species are sensitive. Additionally, our focus on species that tend to occupy fairly natural environments that are little altered by humans, such as alpine and subalpine landscapes or low‐deteriorated forests, might also play a role. The distribution of such habitats could indeed be mainly driven by climate (Thuiller, Araújo, and Lavorel 2004) or by other environmental variables directly linked to climate, such as altitude (Montgomery 2006).

Even so, when changes in climate and land cover were decoupled by allowing only one of them to change and their effects analysed separately, we found evidence that land cover changes significantly influence range size predictions. Specifically, predicted range decreases were lower when changes in climate occurred without the corresponding land cover changes, highlighting the crucial role of habitat availability in modulating species' ability to respond to climate shifts. Our approach is conservative, as land cover projections are implicitly linked to climate (Chen et al. 2020). We expect that modelling land cover change entirely independently of climate would likely reveal even stronger or more divergent outcomes between model predictions that consider only climate versus those that incorporate both climate and land cover changes.

There was also variation in the size of areas predicted as suitable under the different considered scenarios, particularly in relation to climate (CLIMLAND_both_ and CLIMLAND_clim_ models). In general, scenarios that lead to higher maximum temperatures (SSP2‐4.5, Table 1) produced predictions with higher decreases in the area of habitat. Indeed, maximum temperatures were identified as a key limiting factor for the study species' distributions (Figure 1). Increased temperatures may threaten bird species through various mechanisms, including physiological stress, shifts in local resources, and altered biotic interactions (Şekercioğlu et al. 2012). In several taxonomic groups, a tendency has been observed to move towards higher altitudes and latitudes in response to increases in temperature (Sekercioglu et al. 2008; Devictor et al. 2008). In our study, changes in the sizes of suitable areas were also accompanied by a small north‐eastward shift in their distributions when climate changes were considered. This shift is likely driven by changing climatic conditions leading to major losses in suitable areas at southern latitudes, which already represent more extreme conditions limiting their distributions. Some of the study species generally live in alpine and subalpine flats of higher‐latitude regions in northern Iberia, and they can thus not move to much more northern Iberian areas, limiting possible range shifts. In contrast, when only changes in land cover were considered, predicted shifts in suitable areas for most species were relatively small and presented a southern component, particularly for species whose suitable areas were predicted to expand. These findings reinforce the notion that climate and land cover changes, although often occurring in tandem, can have different and sometimes contrasting impacts on biodiversity.

As for the most affected species, the most drastic reductions in the area of potential suitable habitat as a result of changes in both climate and land cover conditions were predicted for the boreal owl ( *Aegolius funereus* ) and the Eurasian treecreeper ( *Certhia familiaris* ), with losses of ≥ 79% of suitable habitat in the study area under all scenarios considered. Other species that are expected to be severely affected by climate and land cover changes (more than 85% decreases under some scenarios) are the rock ptarmigan ( *Lagopus muta* ), the white‐winged snowfinch ( *Montifringilla nivalis* ), the Alpine accentor ( *Prunella collaris* ) and the ring ouzel ( *Turdus torquatus* ). All of these high‐altitude species are expected to be among those more negatively affected by changes in climate conditions, but also by land cover changes (Figure S36) (Fumy and Fartmann 2021). Most of these species already have very small and restricted populations in Iberia (Madroño et al. 2004) and would thus be very sensitive to shifts in suitable habitat availability.

Vulnerability assessment based on ENMs represents a primary approach to support and prioritise policies and actions aimed at preventing the effects of environmental change on biodiversity. However, multiple environmental threats, such as those arising from shifts in climate and land cover, often simultaneously affect species (Barbet‐Massin et al. 2012; Titeux et al. 2016; Zamora‐Gutierrez et al. 2018). In cases where land cover is strongly linked to climate patterns, climate‐based models may serve as sufficient risk assessment tools (Thuiller, Araújo, and Lavorel 2004). However, land cover patterns are frequently influenced not only by climate but also by other processes and human actions, such as preferential destruction or promotion of particular habitat types or management practices (Cardador et al. 2015; Cadieux et al. 2020; Labadie et al. 2024). Therefore, considering both climate and land cover together may provide a more comprehensive understanding of future conservation challenges (Zamora‐Gutierrez et al. 2018). While our broad‐scale species occurrence data have certain limitations—such as potential survey biases in opportunistic data and the inclusion of occurrences of some non‐breeding individuals, these may have introduced noise rather than directional bias. Therefore, they do not undermine our finding that incorporating land cover variables can influence range predictions, even at small rates, particularly when they are not coupled with climate changes.

From a conservation perspective, our findings underscore the urgency of promoting more sustainable development pathways—not only because they lead to lower losses of suitable habitat compared to other scenarios (e.g., SSP1–2.6 vs. SSP2–4.5), but also because, even under these more favourable conditions, significant shifts in species distributions are still expected. This reinforces the need for proactive sustainability and conservation policies, as even the least damaging scenarios do not fully eliminate biodiversity risks. Moreover, because land cover can be more readily influenced through local and regional actions, targeted habitat‐focused interventions offer practical and effective means to enhance biodiversity resilience—even if only modestly—in the face of ongoing environmental change.

## Author Contributions

**Laura Cardador:** conceptualization (lead), data curation (equal), formal analysis (lead), writing – original draft (lead), writing – review and editing (equal). **Irene Batlle:** data curation (equal), formal analysis (lead), writing – original draft (lead), writing – review and editing (equal). **A. Márcia Barbosa:** data curation (equal), formal analysis (equal), writing – review and editing (equal). **Neftalí Sillero:** data curation (equal), formal analysis (equal), writing – review and editing (equal). **Luís Reino:** conceptualization (lead), data curation (equal), formal analysis (equal), writing – review and editing (equal).

## Conflicts of Interest

The authors declare no conflicts of interest.

## Supporting information


Data S1.

## Data Availability

Data and code used in this article can be accessed at https://doi.org/10.5061/dryad.dv41ns26c.
