# Supplementary material for: Land Cover Constrains Range Shifts in Northern Iberian Bird Species Under Climate Change Scenarios
Source: Ecol Evol. 2025 Jul 30;15(8):e71863. doi: 10.1002/ece3.71863 (PMC12310289; doi:10.1002/ece3.71863)
Supplement: Supplementary file 1 — Data S1. [file ECE3-15-e71863-s001.docx]

Supplementary Information

Content

**Figure S1.** Occurrence maps of study species in the Iberian Peninsula.

**Figure S2.** Deviance partitioning analyses per species.

**Figure S3.** Importance of climate and land cover variables in Iberia per species.

**Figures S4 – S35.** Partial response curves for predictors used in CLIMLAND ensemble models for each species.

**Figure S36.** Predicted shifts in the extent of species ranges under different scenarios when both climate and land cover variables, only climate or only land cover are assumed to change for CLIMLAND model predictions.

**Figure S37.** Predicted shifts in species range centroids under different scenarios when both habitat and land cover variables, only climate or only land cover are assumed to change.

**Figure S38.** Cumulative map predictions of suitable habitat for different species under different scenarios when both climate and land cover variables, only climate or only land cover are assumed to change.

**Figures S39-S70.** Map predictions of suitable habitat for each species under different scenarios when both climate and land cover variables, only climate or only land cover are assumed to change.

**Table S1.** GBIF occurrence downloads.

**Table S2.** Climate and land cover characteristics of socio-economic scenarios based on general circulation model MIROC.

**Table S3.** Model accuracy metrics.

**Table S4.** Wilcoxon pairwise comparisons of the area of habitat between model predictions.

**Table S5.** Wilcoxon pairwise comparisons of the area of habitat across future scenarios.

**Table S6.** Wilcoxon pairwise comparisons of the centroid position (longitude and latitude) of suitable habitat **in Iberia** and their distance to the centroid of current suitable habitat across scenarios.

**Table S7.** Wilcoxon pairwise comparisons of the centroid position (longitude and latitude) of suitable habitat and their distance to the centroid of current suitable habitat across models.

**Table S8.** Wilcoxon pairwise comparisons of the centroid position (longitude and latitude) of suitable habitat within species **current presences** and their distance to the centroid of current suitable habitat across scenarios.

**Figure S1**. Occurrence maps of study species in the Iberian Peninsula according to 10×10 km Spanish and Portuguese bird atlas.


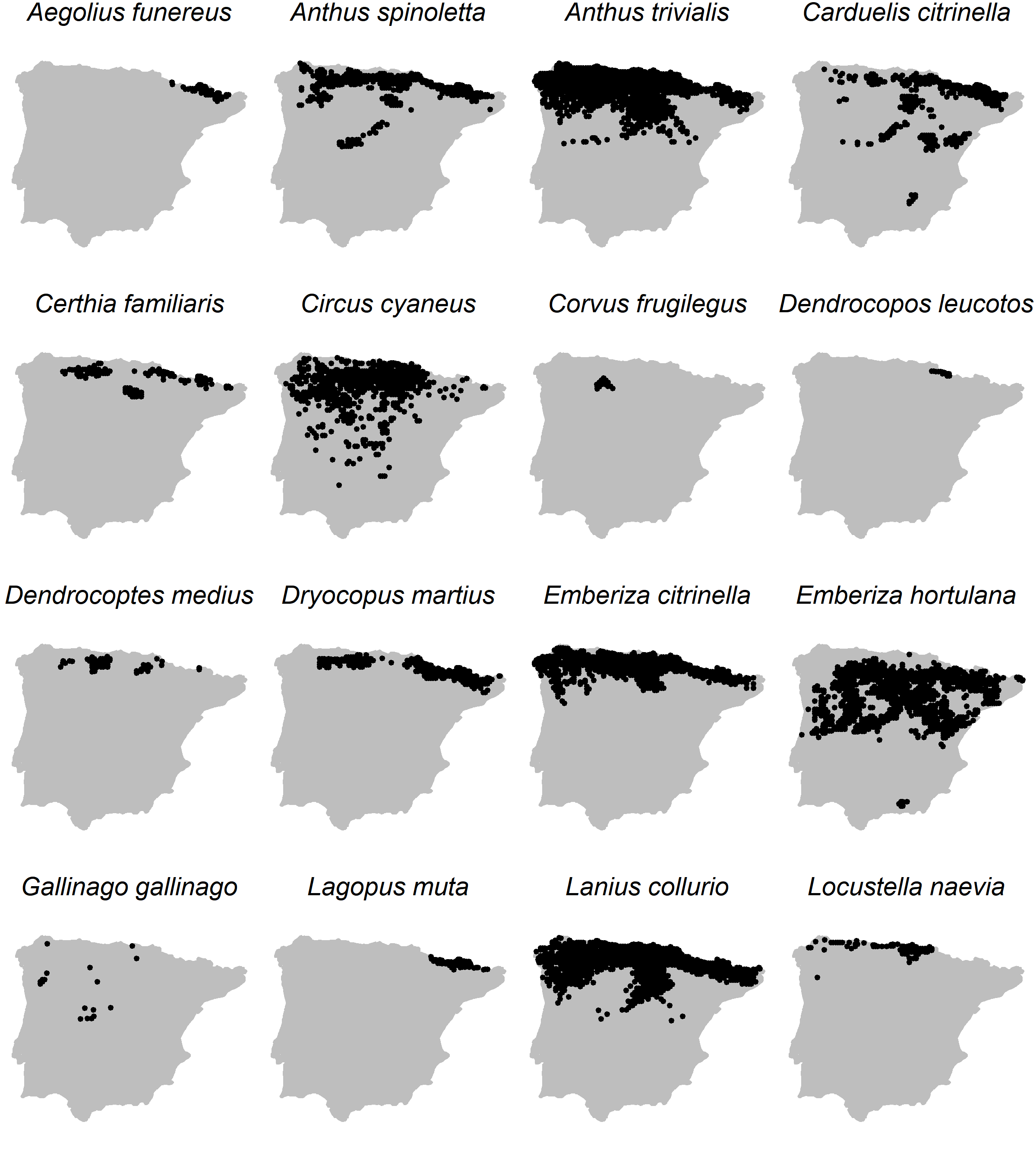


**Figure S1.** Continued.


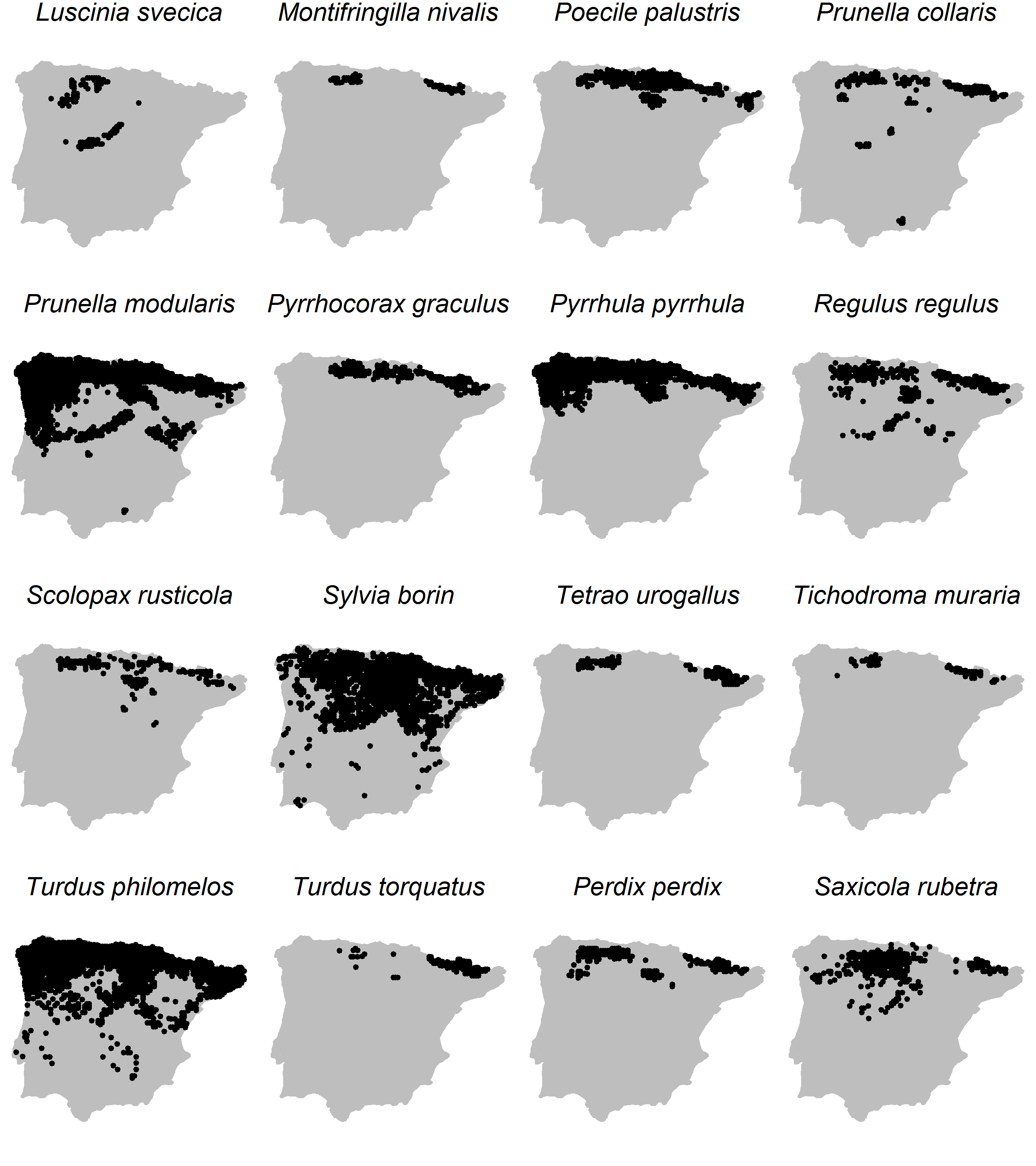


**Figure S2.** Results of deviance partitioning analyses for the probability of occurrence of 32 study species within their global breeding ranges. Deviance is explained by the pure effects of climate (clim) and land cover (land) and their joint effect (joint).


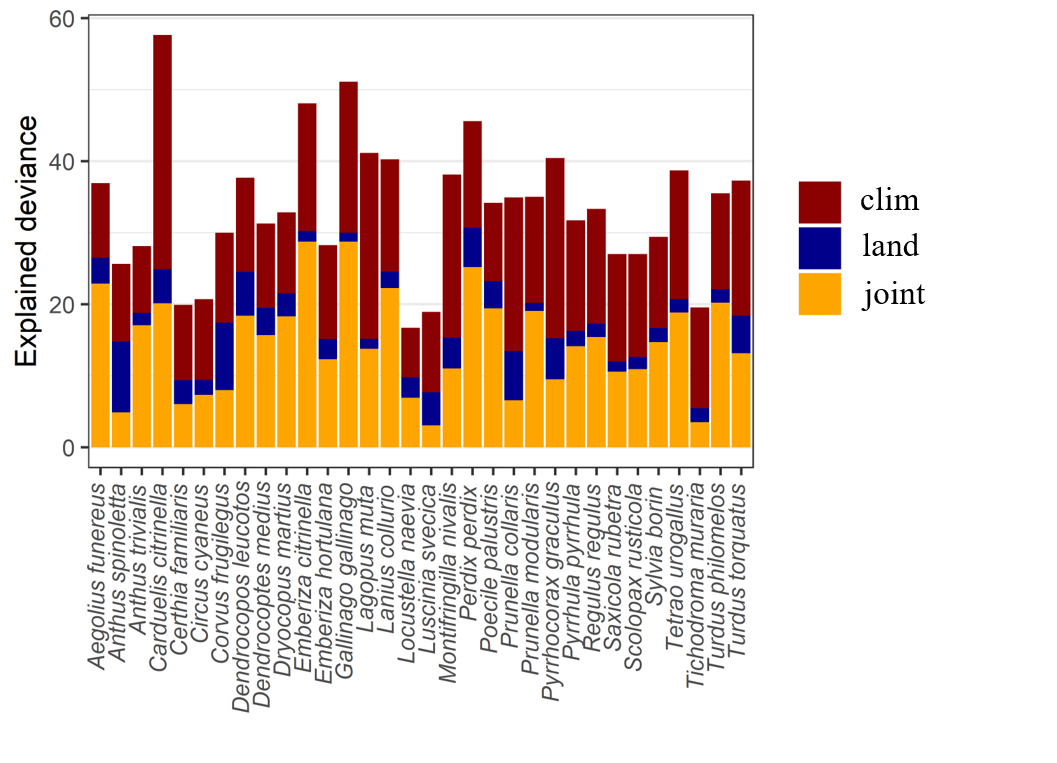


**Figure S3**. Importance of climate, land cover and bias variables in environmental niche models of study bird species in Iberia. Tmin = minimum temperature of the coldest month, Tmax = maximum temperature of the warmest month, Pan = annual precipitation: Pcv = precipitation seasonality: Needleleaf = needleleaf evergreen forests, Broadleaf = broadleaf deciduous forests, Bias = sampling bias variable. Note that the variables for each species appear in the same order as listed in the legend.

**
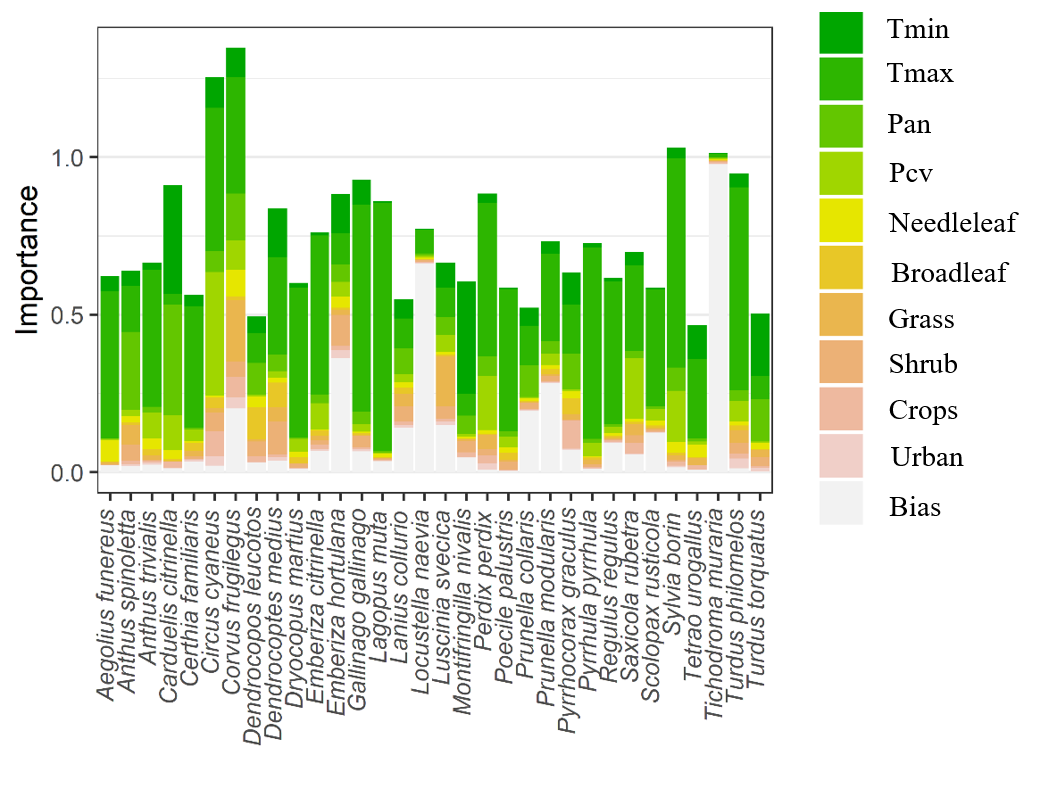
**

**Figure S4.** Partial response curves for different predictors used in CLIMLAND ensemble models for *Aegolius funereus*. Tmax = maximum temperature of the warmest month (ºC); tmin = minimum temperature of the coldest month (ºC), prec = annual precipitation (mm), precsd = precipitation seasonality: ne = needleleaf evergreen forests (%), bd = broadleaf deciduous forests (%), gr = grassland (%), sh = shrubs (%), cr = cops (%), ur = urban (%), bias = sampling bias variable.


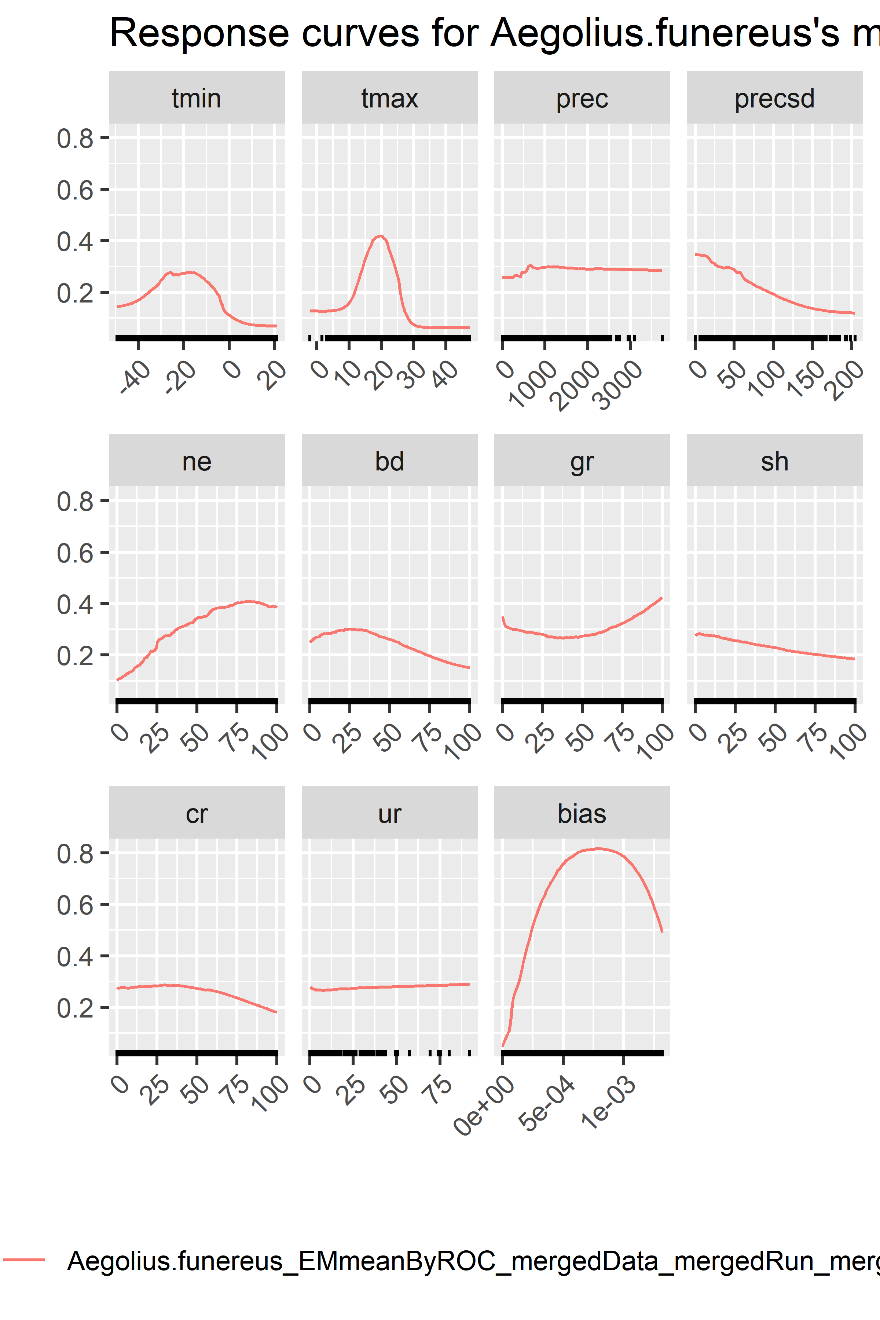


**Figure S5.** Partial response curves for different predictors used in CLIMLAND ensemble models for *Anthus spilonetta*. Tmax = maximum temperature of the warmest month (ºC); tmin = minimum temperature of the coldest month (ºC), prec = annual precipitation (mm), precsd = precipitation seasonality: ne = needleleaf evergreen forests (%), bd = broadleaf deciduous forests (%), gr = grassland (%), sh = shrubs (%), cr = cops (%), ur = urban (%), bias = sampling bias variable.


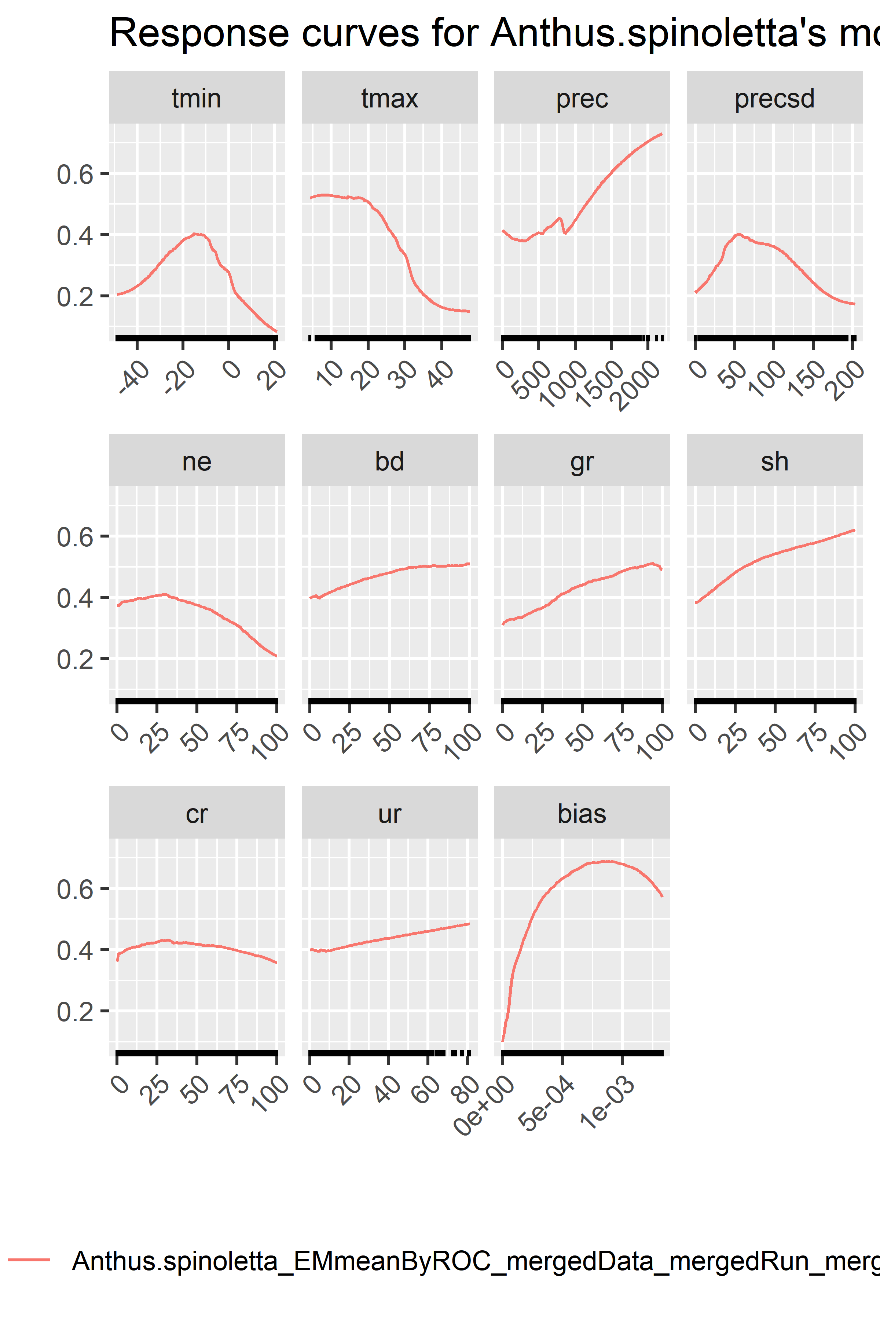


**Figure S6.** Partial response curves for different predictors used in CLIMLAND ensemble models for *Anthus trivialis*. Tmax = maximum temperature of the warmest month (ºC); tmin = minimum temperature of the coldest month (ºC), prec = annual precipitation (mm), precsd = precipitation seasonality: ne = needleleaf evergreen forests (%), bd = broadleaf deciduous forests (%), gr = grassland (%), sh = shrubs (%), cr = cops (%), ur = urban (%), bias = sampling bias variable.


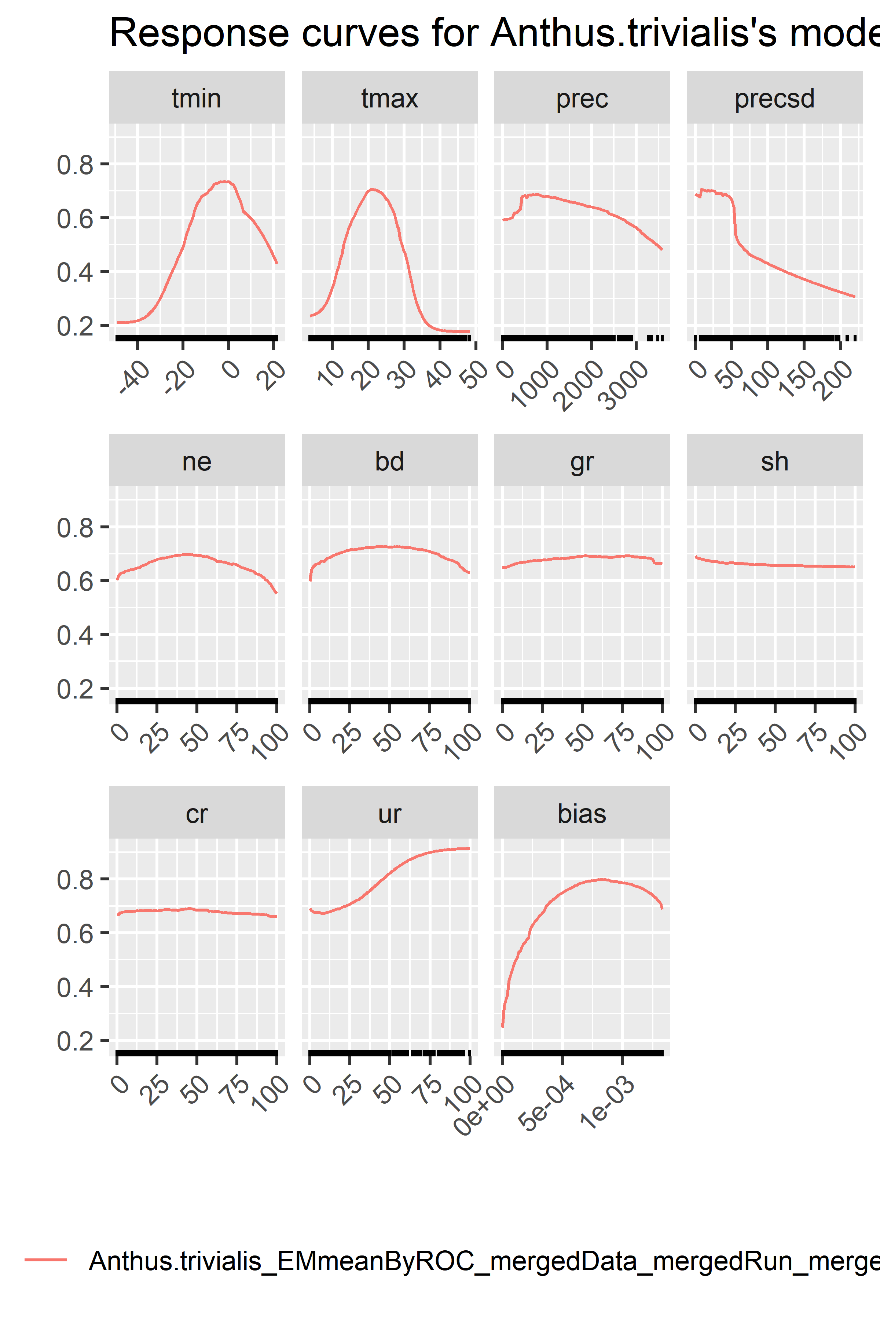


**Figure S7.** Partial response curves for different predictors used in CLIMLAND ensemble models for *Carduellis citrinella*. Tmax = maximum temperature of the warmest month (ºC); tmin = minimum temperature of the coldest month (ºC), prec = annual precipitation (mm), precsd = precipitation seasonality: ne = needleleaf evergreen forests (%), bd = broadleaf deciduous forests (%), gr = grassland (%), sh = shrubs (%), cr = cops (%), ur = urban (%), bias = sampling bias variable.


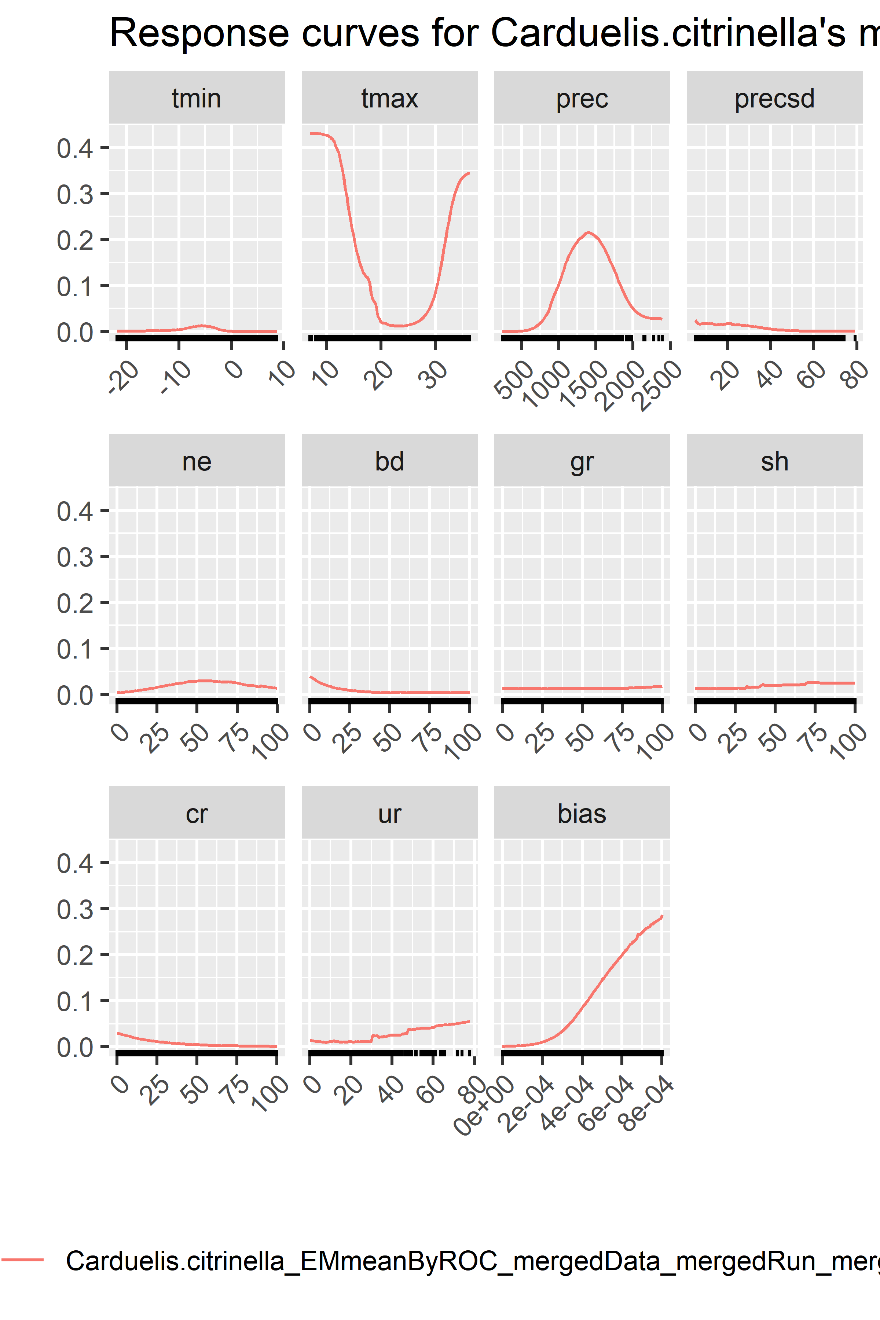


**Figure S8.** Partial response curves for different predictors used in CLIMLAND ensemble models for *Certhia familiaris*. Tmax = maximum temperature of the warmest month (ºC); tmin = minimum temperature of the coldest month (ºC), prec = annual precipitation (mm), precsd = precipitation seasonality: ne = needleleaf evergreen forests (%), bd = broadleaf deciduous forests (%), gr = grassland (%), sh = shrubs (%), cr = cops (%), ur = urban (%), bias = sampling bias variable.


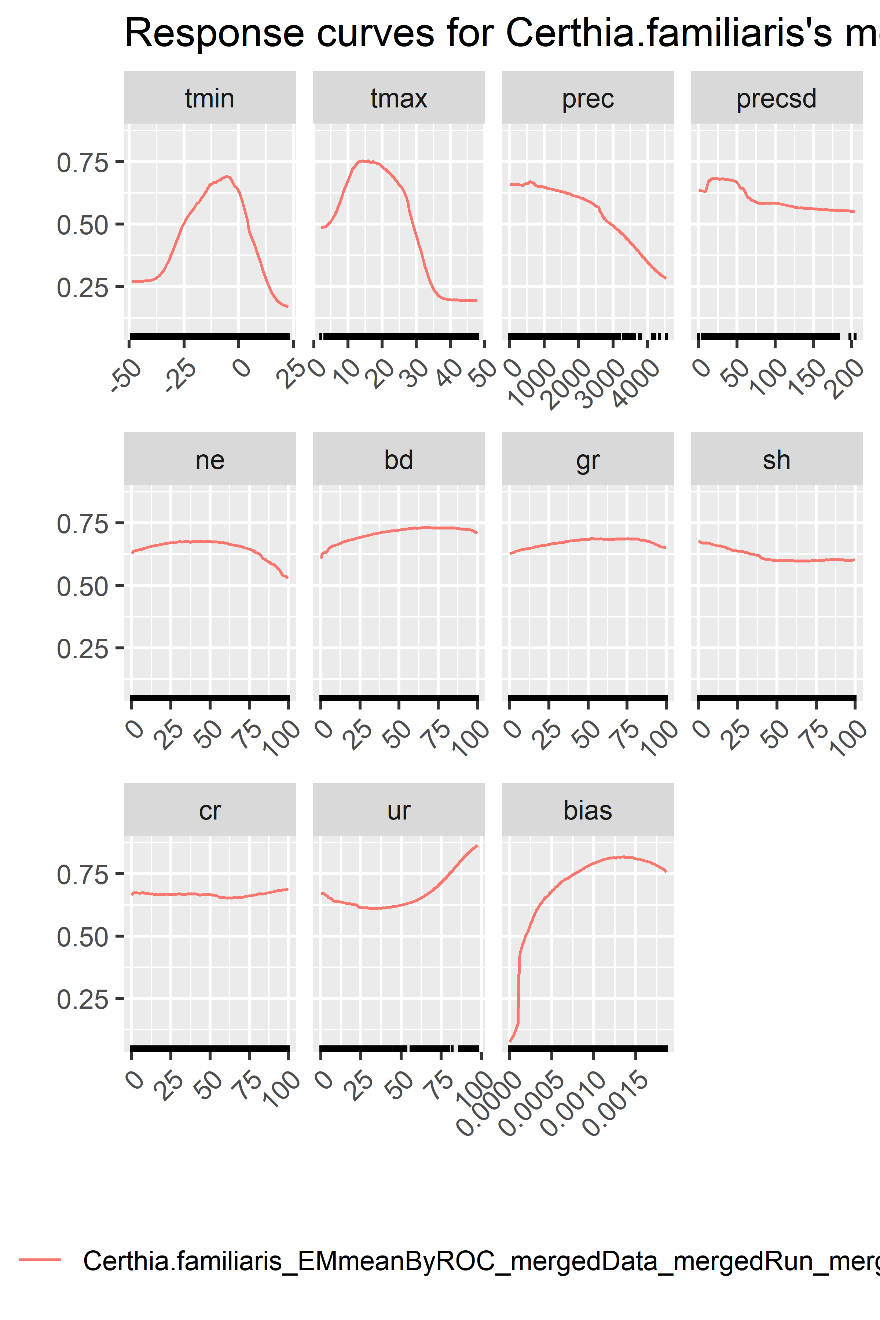


**Figure S9.** Partial response curves for different predictors used in CLIMLAND ensemble models for *Circus cyaneus*. Tmax = maximum temperature of the warmest month (ºC); tmin = minimum temperature of the coldest month (ºC), prec = annual precipitation (mm), precsd = precipitation seasonality: ne = needleleaf evergreen forests (%), bd = broadleaf deciduous forests (%), gr = grassland (%), sh = shrubs (%), cr = cops (%), ur = urban (%), bias = sampling bias variable.


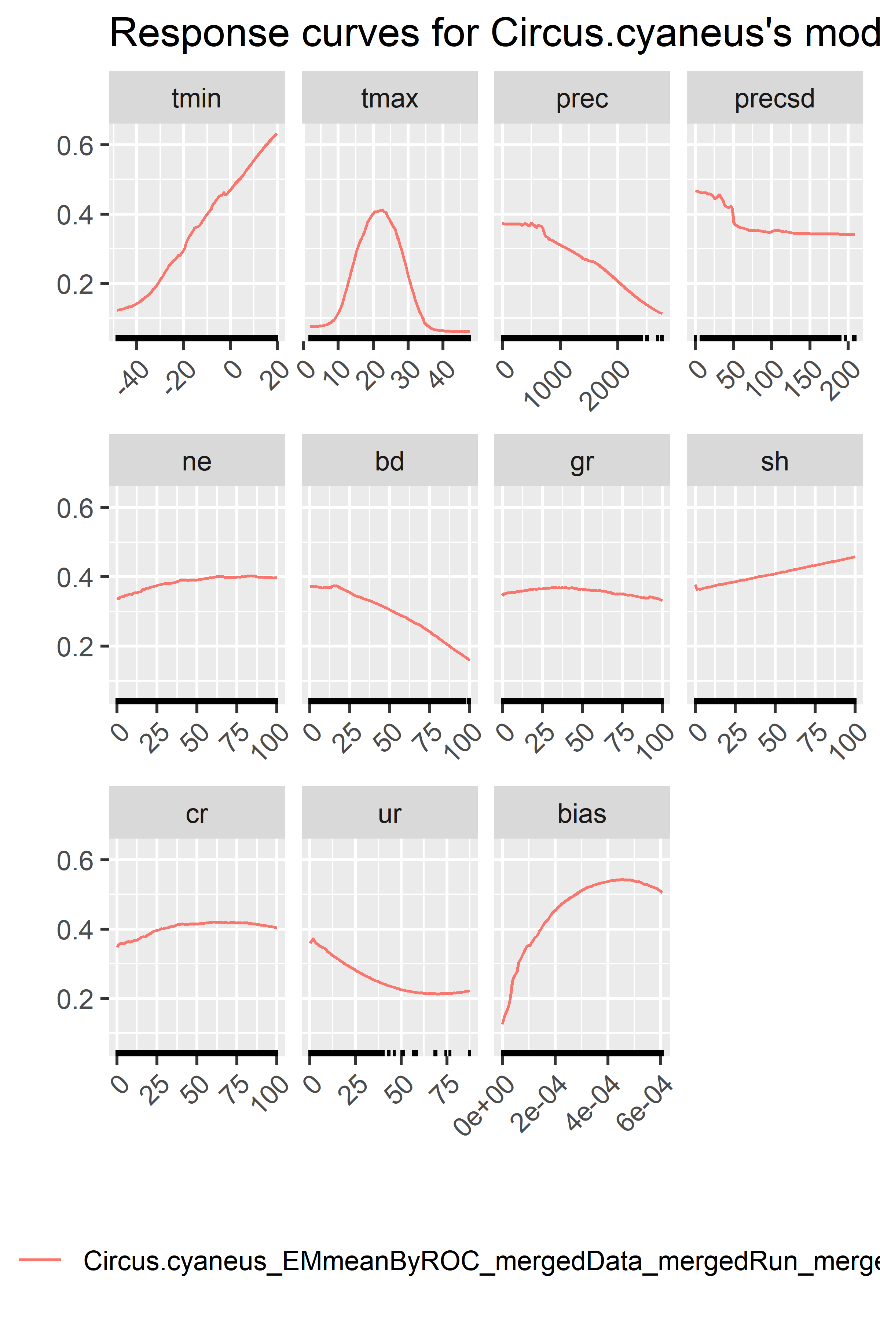


**Figure S10.** Partial response curves for different predictors used in CLIMLAND ensemble models for C*orvus frugilegus*. Tmax = maximum temperature of the warmest month (ºC); tmin = minimum temperature of the coldest month (ºC), prec = annual precipitation (mm), precsd = precipitation seasonality: ne = needleleaf evergreen forests (%), bd = broadleaf deciduous forests (%), gr = grassland (%), sh = shrubs (%), cr = cops (%), ur = urban (%), bias = sampling bias variable.


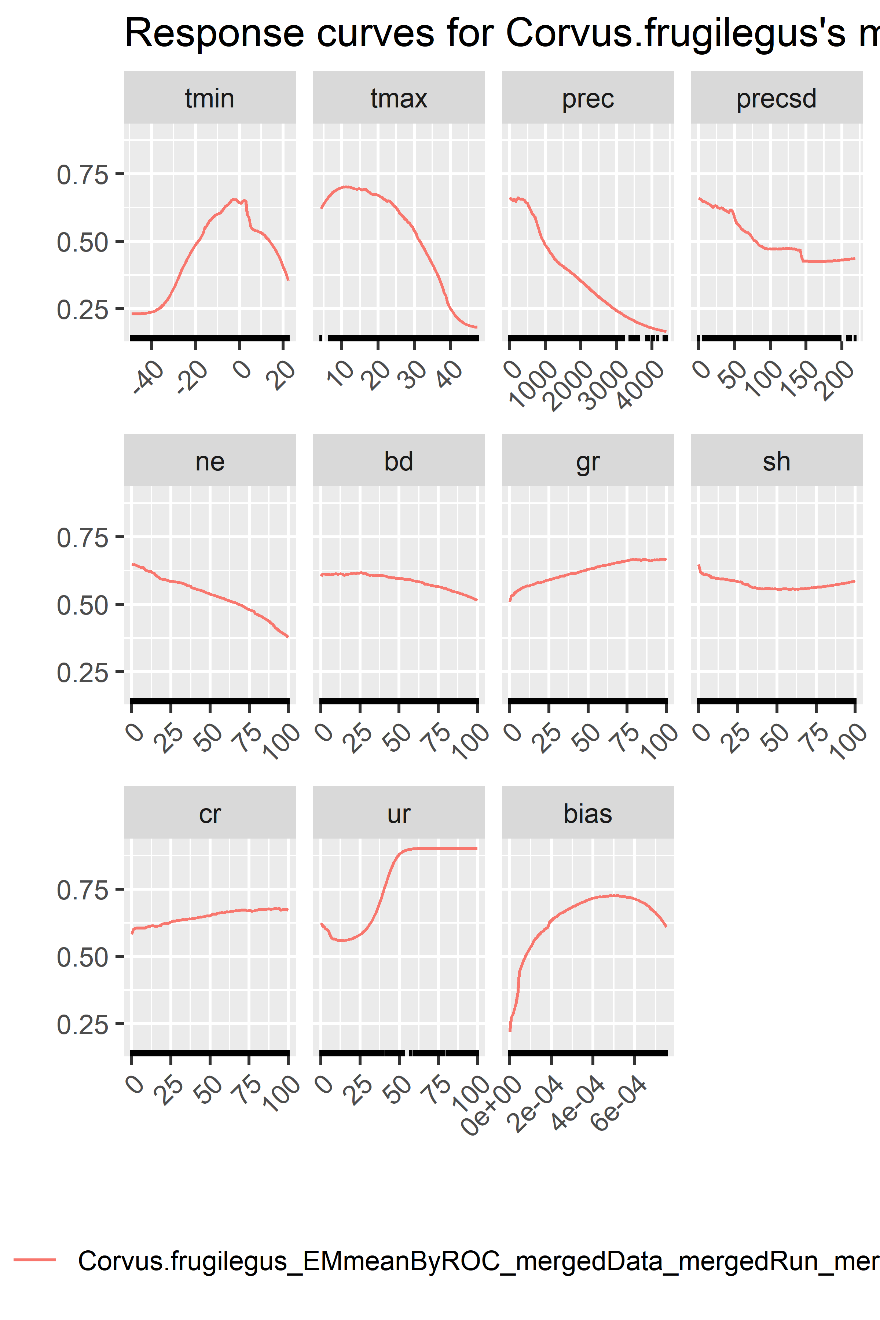


**Figure S11.** Partial response curves for different predictors used in CLIMLAND ensemble models for *Dendrocopos leucotos*. Tmax = maximum temperature of the warmest month (ºC); tmin = minimum temperature of the coldest month (ºC), prec = annual precipitation (mm), precsd = precipitation seasonality: ne = needleleaf evergreen forests (%), bd = broadleaf deciduous forests (%), gr = grassland (%), sh = shrubs (%), cr = cops (%), ur = urban (%), bias = sampling bias variable.


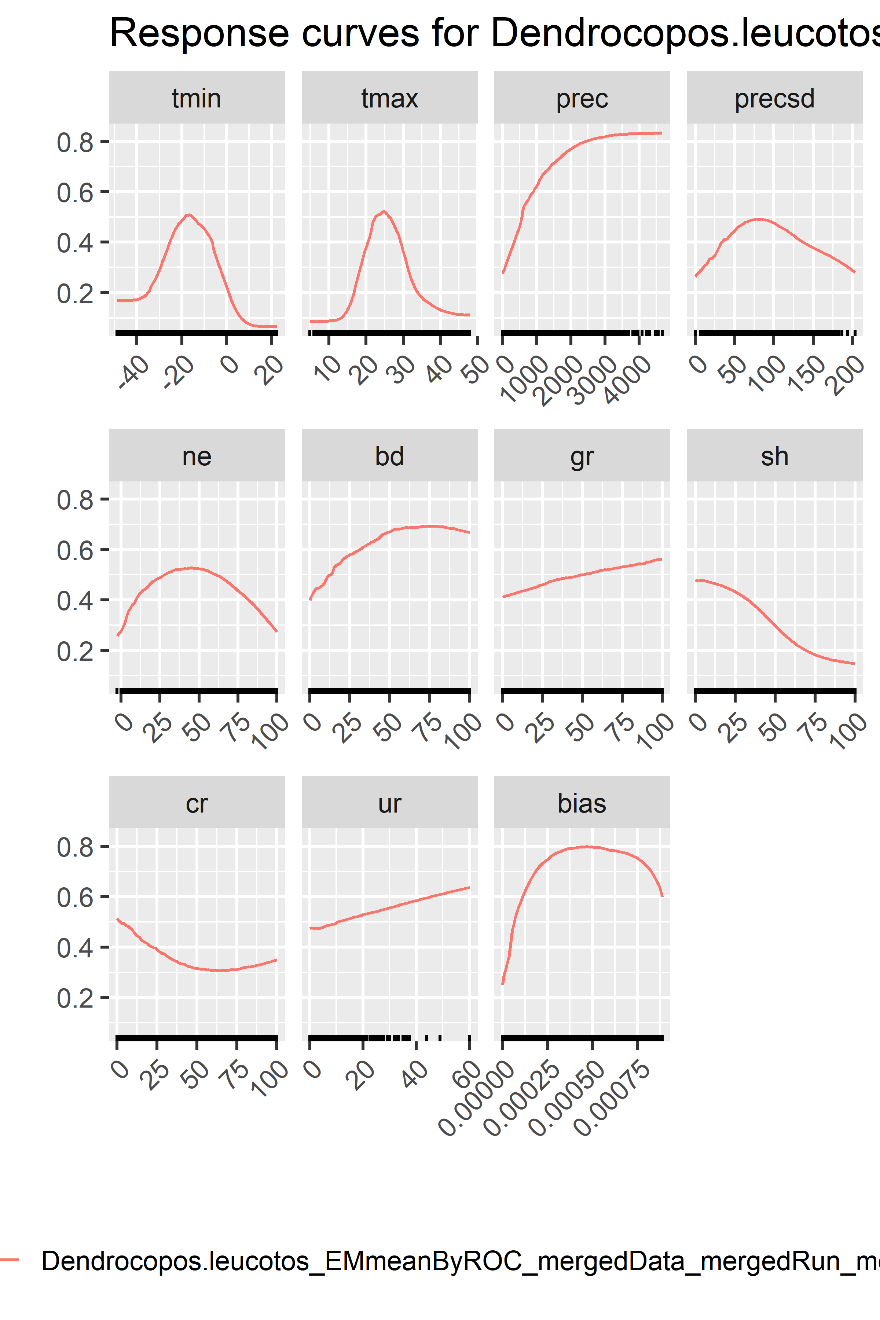


**Figure S12.** Partial response curves for different predictors used in CLIMLAND ensemble models for *Dendrocoptes medius*. Tmax = maximum temperature of the warmest month (ºC); tmin = minimum temperature of the coldest month (ºC), prec = annual precipitation (mm), precsd = precipitation seasonality: ne = needleleaf evergreen forests (%), bd = broadleaf deciduous forests (%), gr = grassland (%), sh = shrubs (%), cr = cops (%), ur = urban (%), bias = sampling bias variable.

**
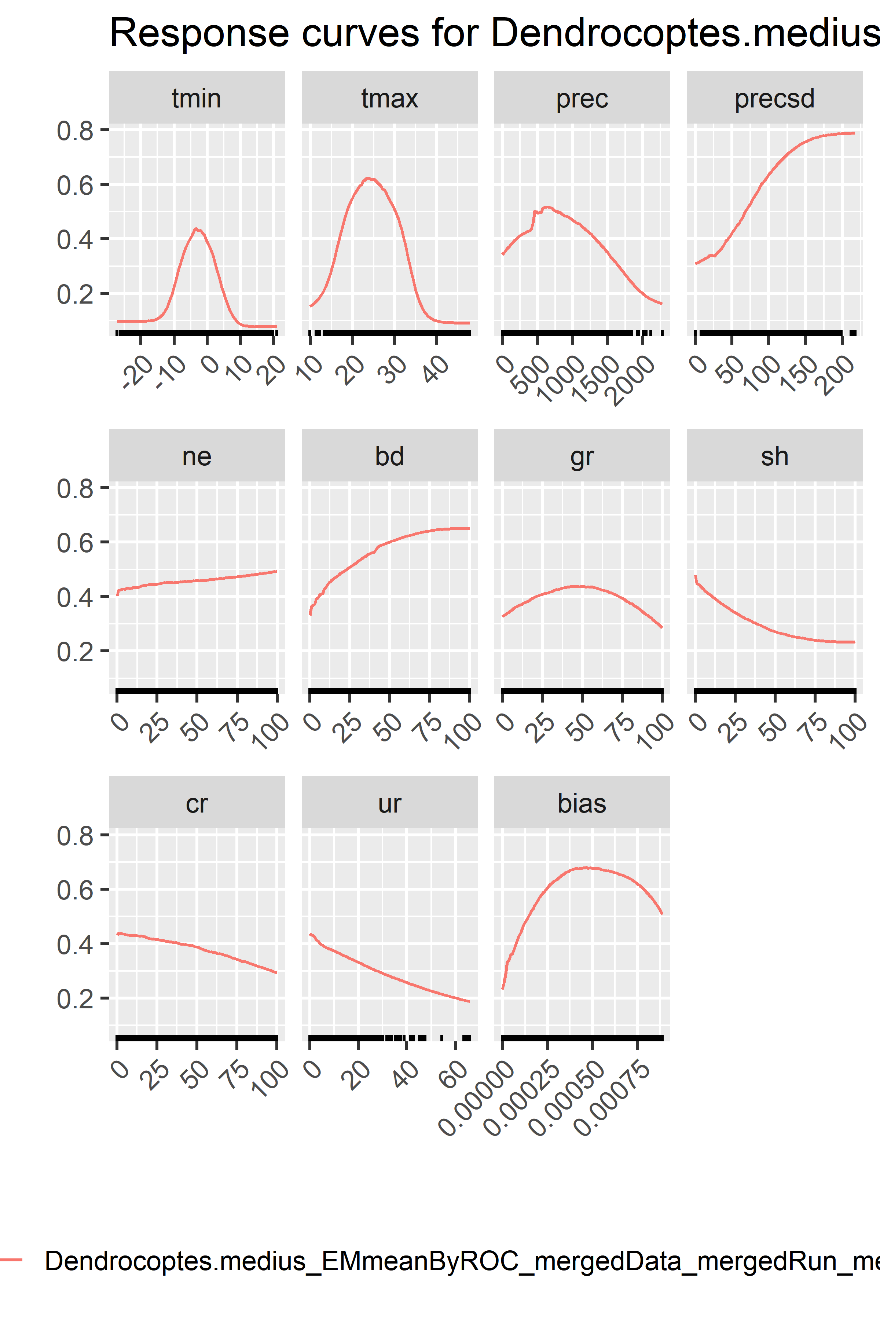
**

**Figure S13.** Partial response curves for different predictors used in CLIMLAND ensemble models for *Dryocopus martius*. Tmax = maximum temperature of the warmest month (ºC); tmin = minimum temperature of the coldest month (ºC), prec = annual precipitation (mm), precsd = precipitation seasonality: ne = needleleaf evergreen forests (%), bd = broadleaf deciduous forests (%), gr = grassland (%), sh = shrubs (%), cr = cops (%), ur = urban (%), bias = sampling bias variable.


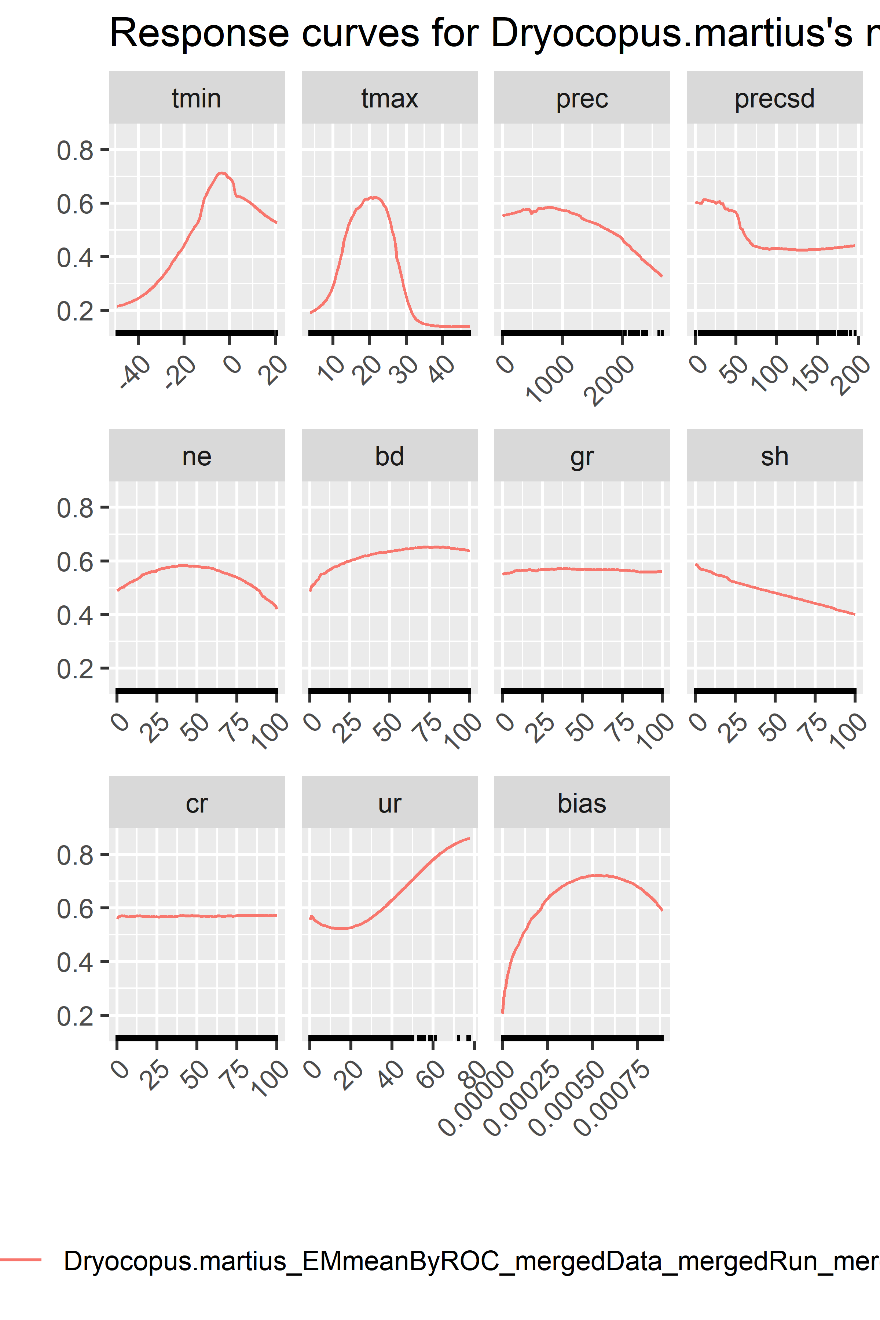


**Figure S14.** Partial response curves for different predictors used in CLIMLAND ensemble models for *Emberiza citrinella*. Tmax = maximum temperature of the warmest month (ºC); tmin = minimum temperature of the coldest month (ºC), prec = annual precipitation (mm), precsd = precipitation seasonality: ne = needleleaf evergreen forests (%), bd = broadleaf deciduous forests (%), gr = grassland (%), sh = shrubs (%), cr = cops (%), ur = urban (%), bias = sampling bias variable.


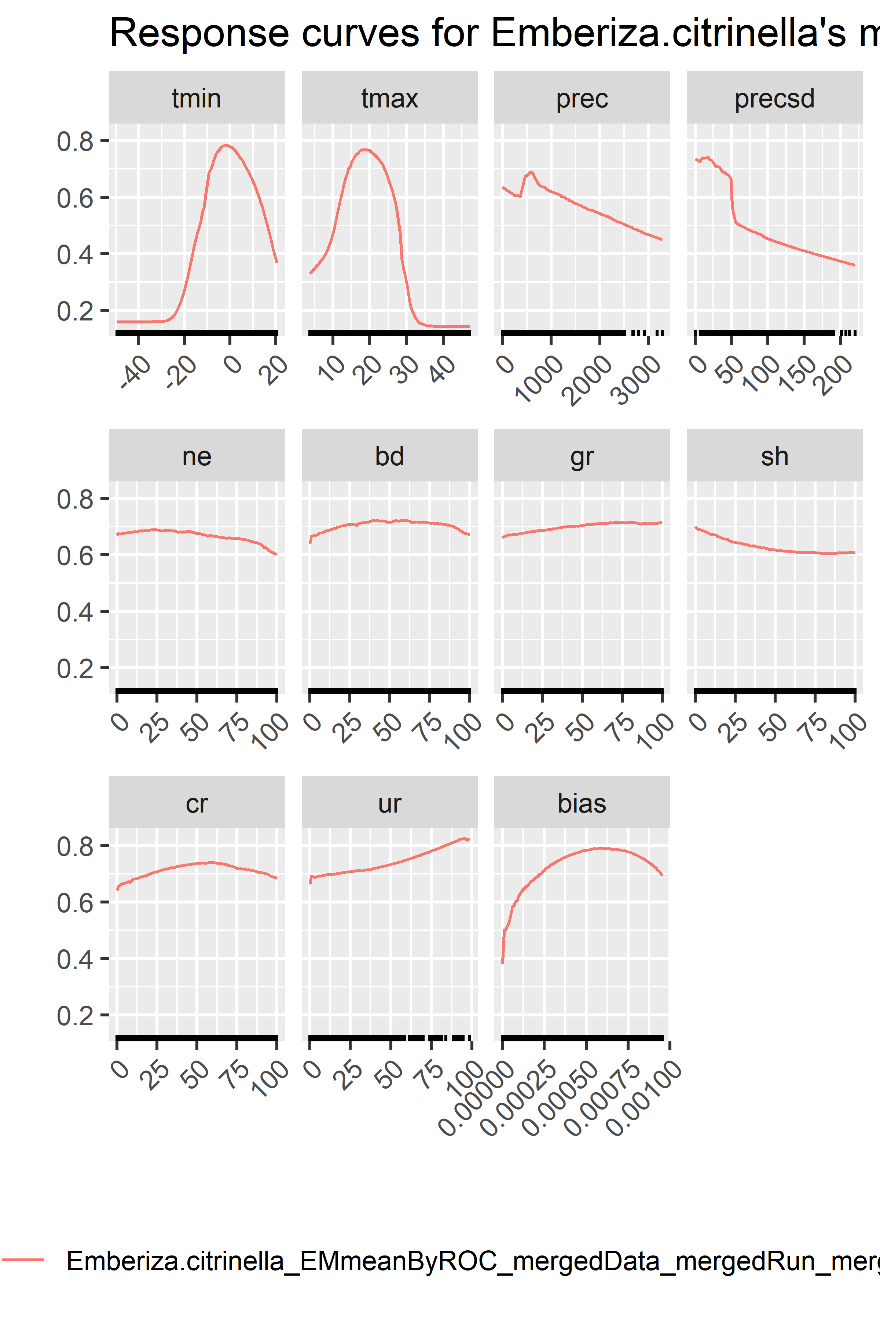


**Figure S15.** Partial response curves for different predictors used in CLIMLAND ensemble models for *Emberiza hortulana*. Tmax = maximum temperature of the warmest month (ºC); tmin = minimum temperature of the coldest month (ºC), prec = annual precipitation (mm), precsd = precipitation seasonality: ne = needleleaf evergreen forests (%), bd = broadleaf deciduous forests (%), gr = grassland (%), sh = shrubs (%), cr = cops (%), ur = urban (%), bias = sampling bias variable.


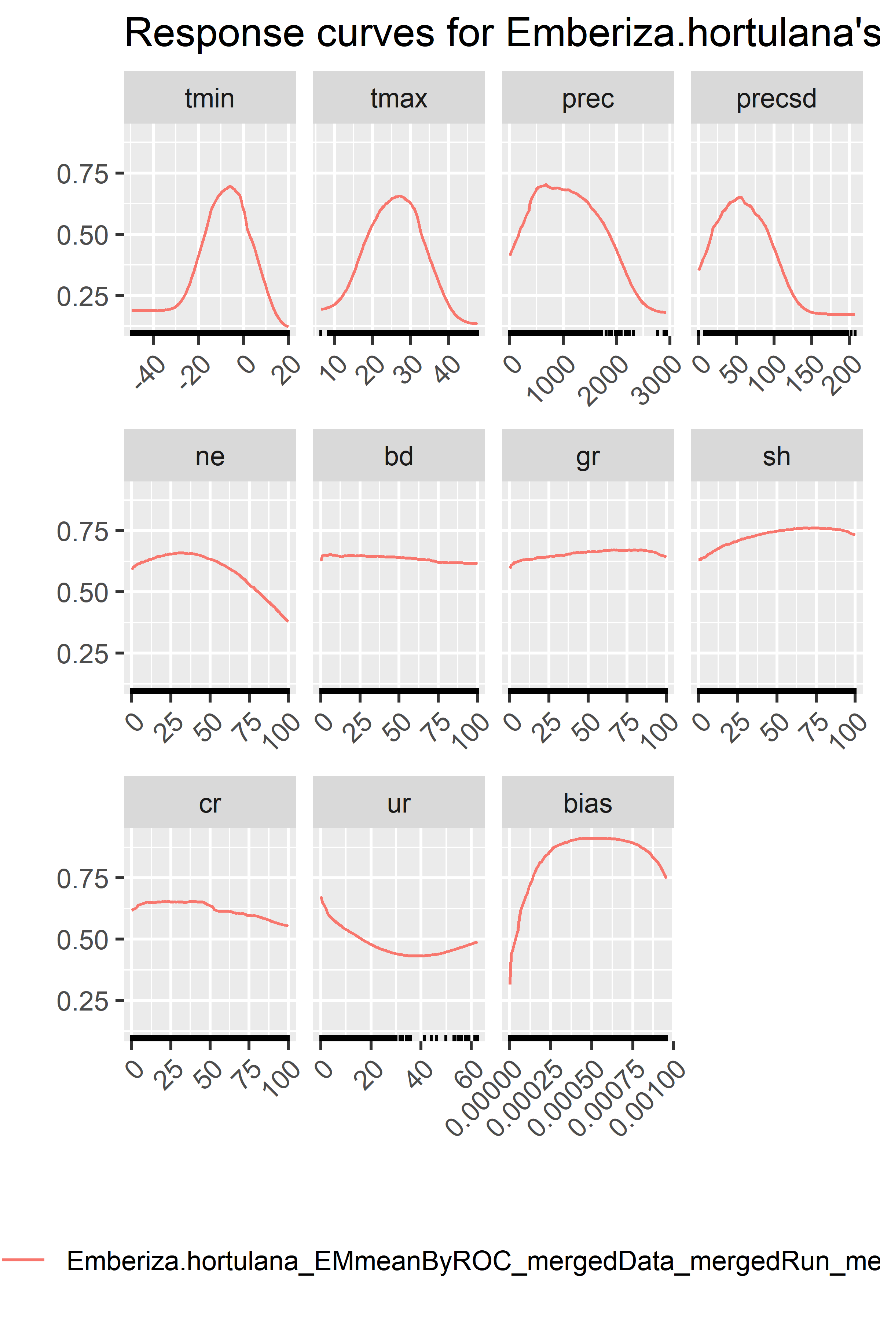


**Figure S16.** Partial response curves for different predictors used in CLIMLAND ensemble models for *Gallinago gallinago*. Tmax = maximum temperature of the warmest month (ºC); tmin = minimum temperature of the coldest month (ºC), prec = annual precipitation (mm), precsd = precipitation seasonality: ne = needleleaf evergreen forests (%), bd = broadleaf deciduous forests (%), gr = grassland (%), sh = shrubs (%), cr = cops (%), ur = urban (%), bias = sampling bias variable.

**
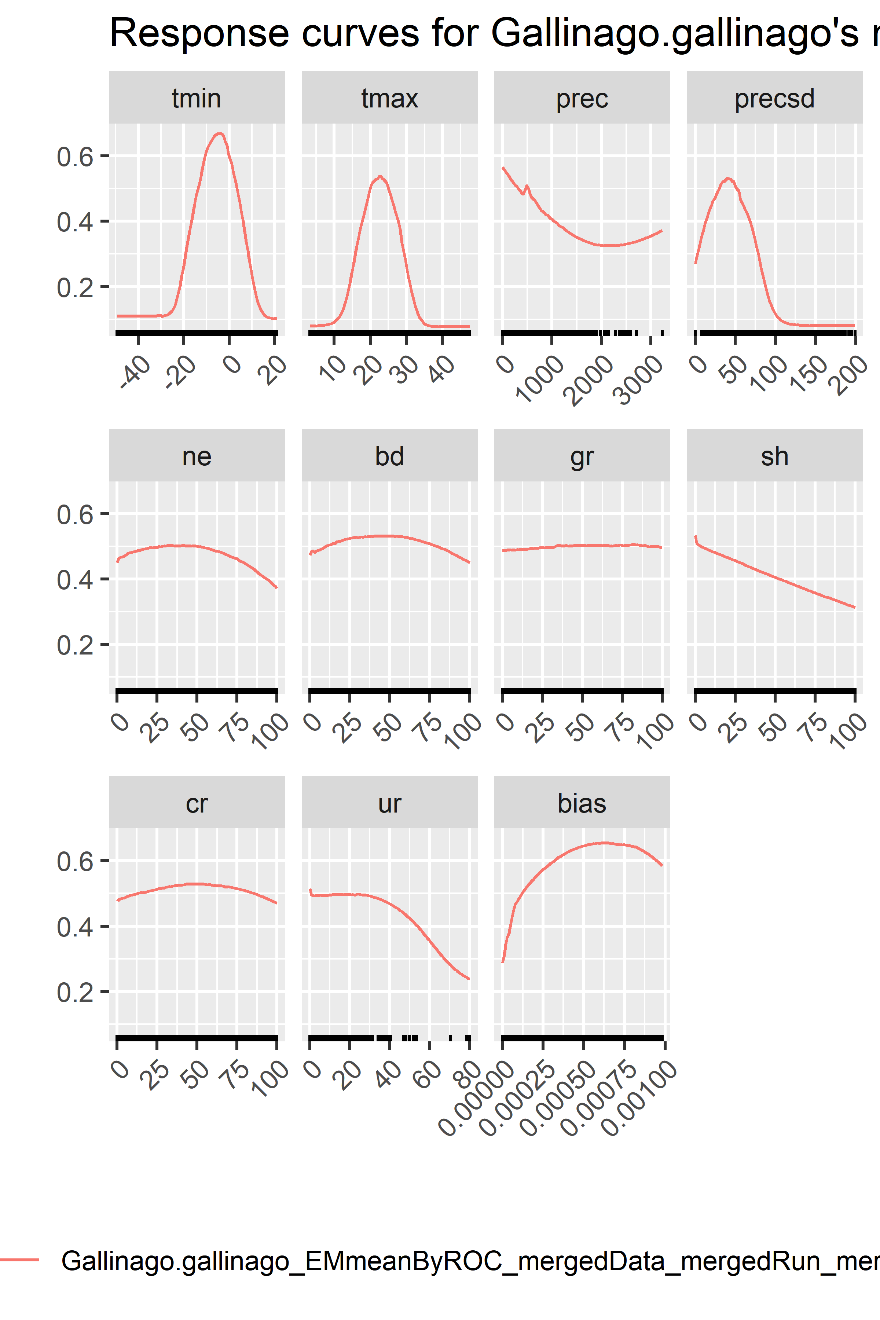
**

**Figure S17.** Partial response curves for different predictors used in CLIMLAND ensemble models for *Lagopus muta*. Tmax = maximum temperature of the warmest month (ºC); tmin = minimum temperature of the coldest month (ºC), prec = annual precipitation (mm), precsd = precipitation seasonality: ne = needleleaf evergreen forests (%), bd = broadleaf deciduous forests (%), gr = grassland (%), sh = shrubs (%), cr = cops (%), ur = urban (%), bias = sampling bias variable.


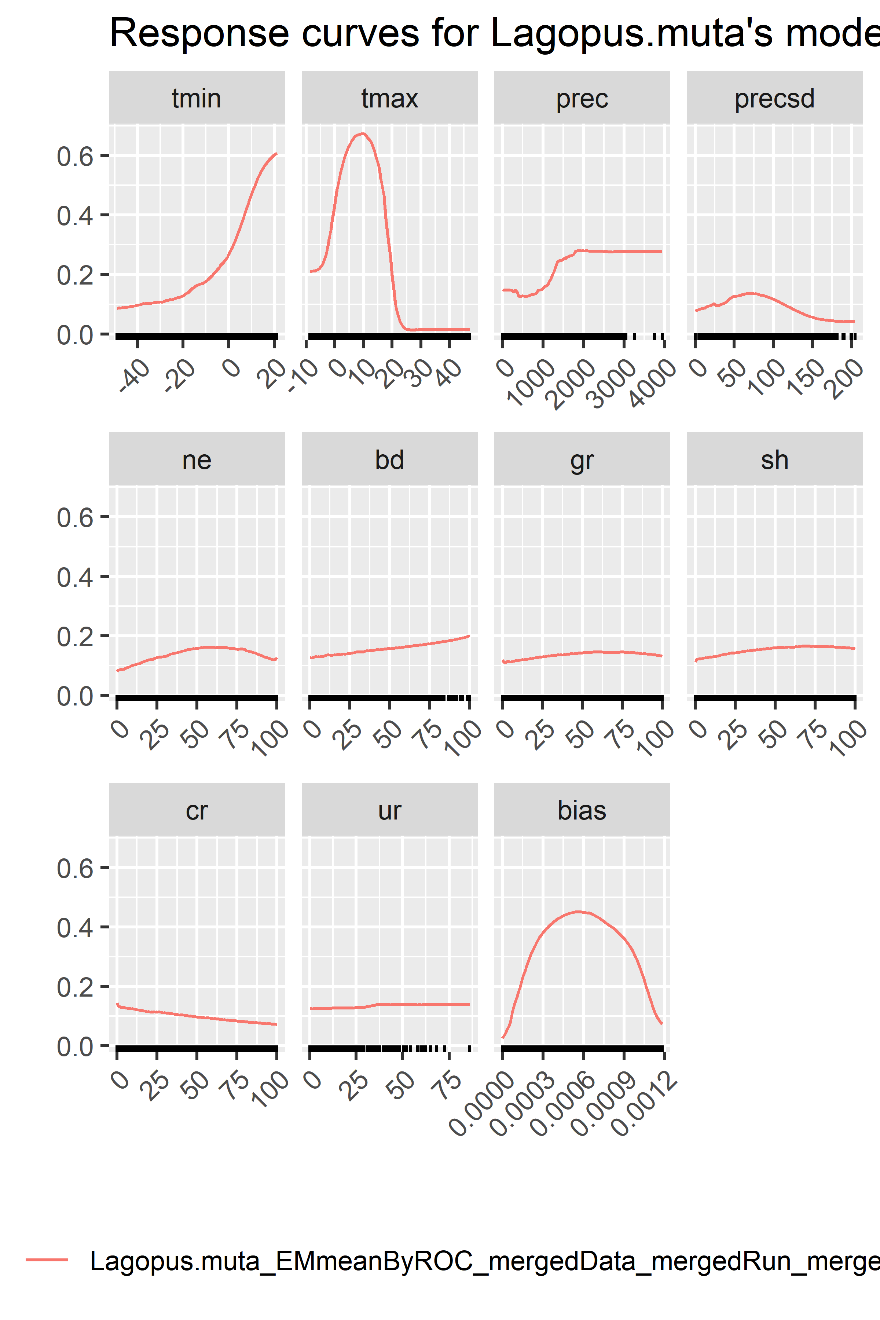


**Figure S18.** Partial response curves for different predictors used in CLIMLAND ensemble models for *Lanius collurio*. Tmax = maximum temperature of the warmest month (ºC); tmin = minimum temperature of the coldest month (ºC), prec = annual precipitation (mm), precsd = precipitation seasonality: ne = needleleaf evergreen forests (%), bd = broadleaf deciduous forests (%), gr = grassland (%), sh = shrubs (%), cr = cops (%), ur = urban (%), bias = sampling bias variable.


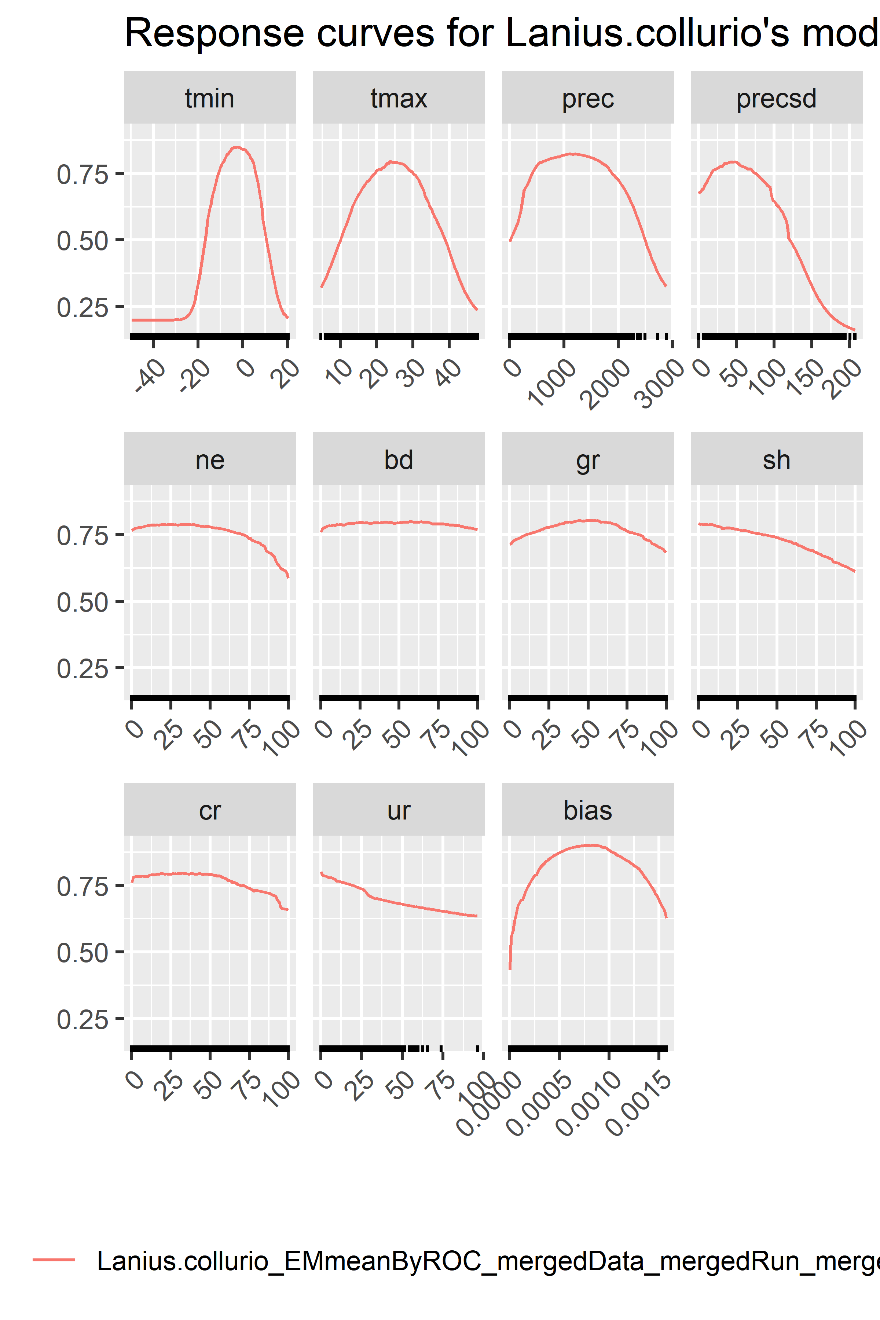


**Figure S19.** Partial response curves for different predictors used in CLIMLAND ensemble models for *Locustella naevia*. Tmax = maximum temperature of the warmest month (ºC); tmin = minimum temperature of the coldest month (ºC), prec = annual precipitation (mm), precsd = precipitation seasonality: ne = needleleaf evergreen forests (%), bd = broadleaf deciduous forests (%), gr = grassland (%), sh = shrubs (%), cr = cops (%), ur = urban (%), bias = sampling bias variable.


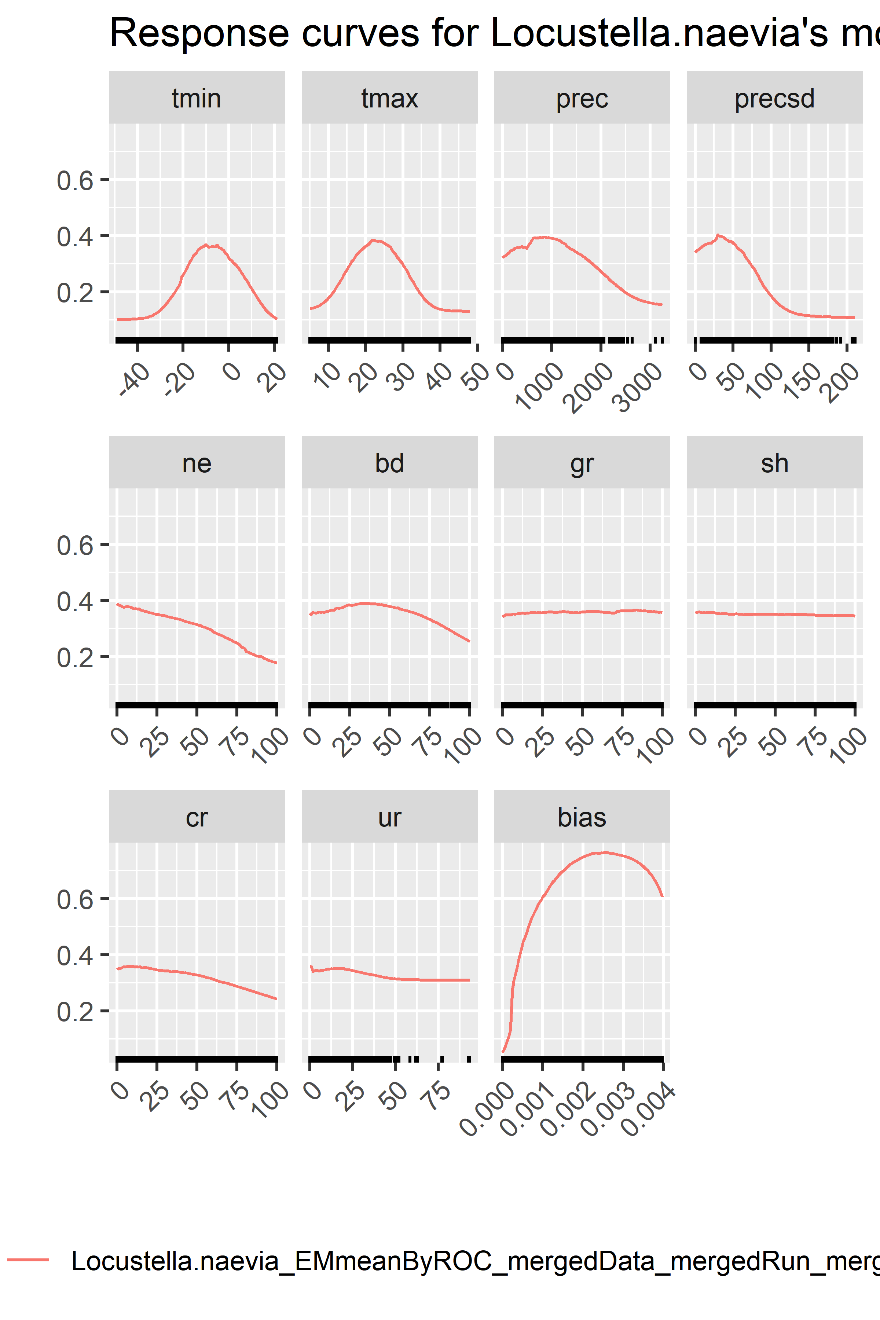


**Figure S20.** Partial response curves for different predictors used in CLIMLAND ensemble models for *Luscinia svecica*. Tmax = maximum temperature of the warmest month (ºC); tmin = minimum temperature of the coldest month (ºC), prec = annual precipitation (mm), precsd = precipitation seasonality: ne = needleleaf evergreen forests (%), bd = broadleaf deciduous forests (%), gr = grassland (%), sh = shrubs (%), cr = cops (%), ur = urban (%), bias = sampling bias variable.


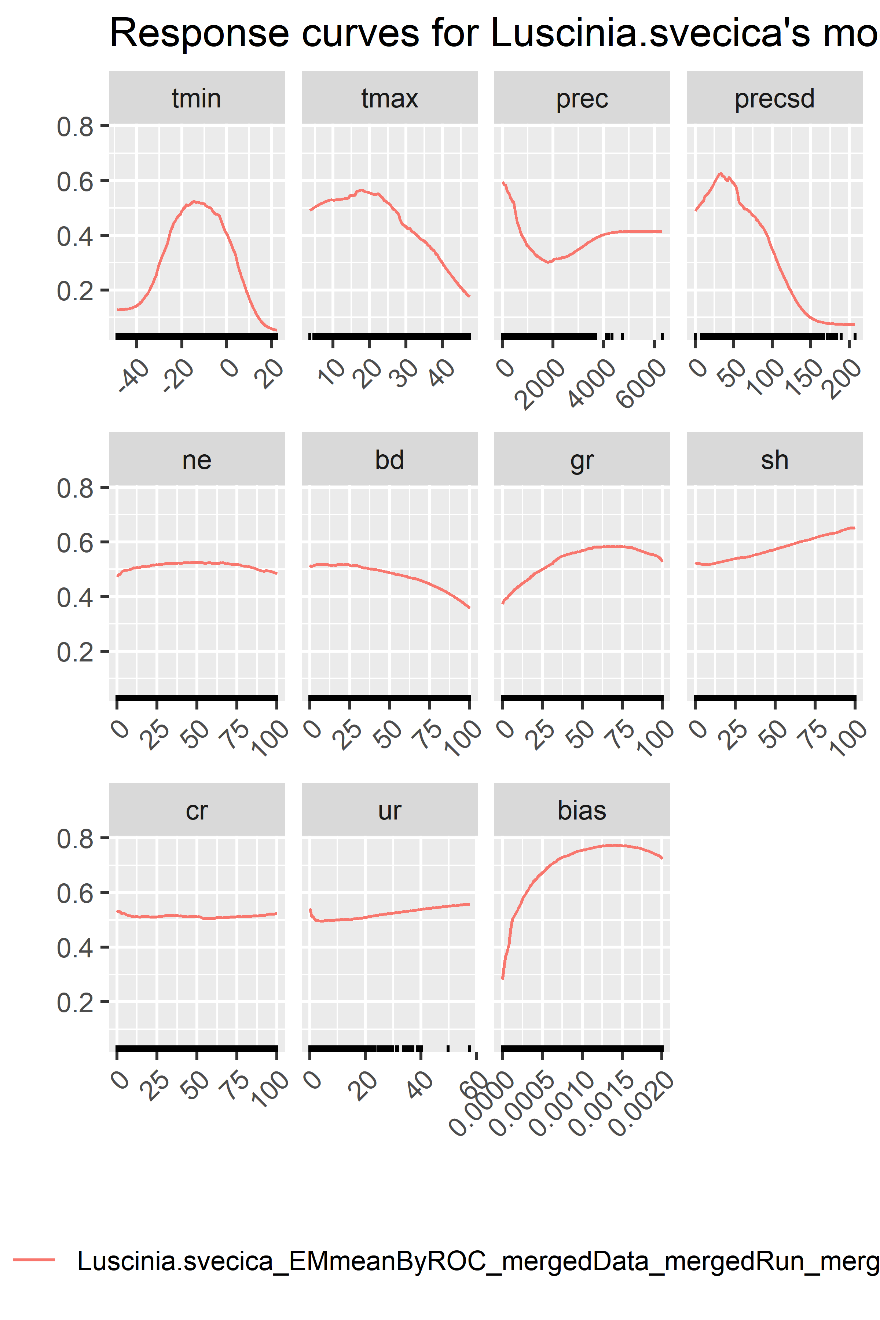


**Figure S21.** Partial response curves for different predictors used in CLIMLAND ensemble models for *Montifringilla nivalis*. Tmax = maximum temperature of the warmest month (ºC); tmin = minimum temperature of the coldest month (ºC), prec = annual precipitation (mm), precsd = precipitation seasonality: ne = needleleaf evergreen forests (%), bd = broadleaf deciduous forests (%), gr = grassland (%), sh = shrubs (%), cr = cops (%), ur = urban (%), bias = sampling bias variable.


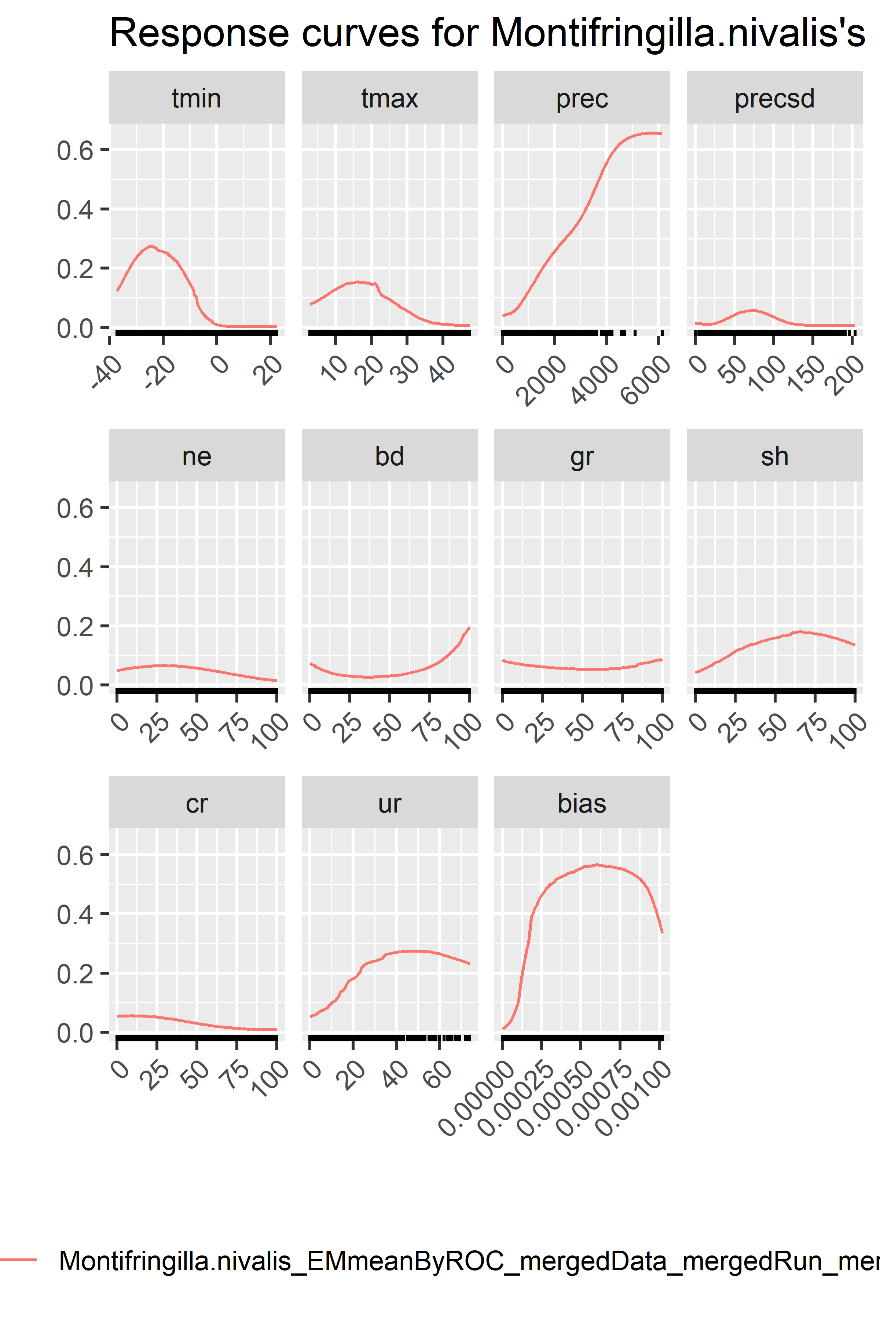


**Figure S22.** Partial response curves for different predictors used in CLIMLAND ensemble models for *Poecile palustris*. Tmax = maximum temperature of the warmest month (ºC); tmin = minimum temperature of the coldest month (ºC), prec = annual precipitation (mm), precsd = precipitation seasonality: ne = needleleaf evergreen forests (%), bd = broadleaf deciduous forests (%), gr = grassland (%), sh = shrubs (%), cr = cops (%), ur = urban (%), bias = sampling bias variable.


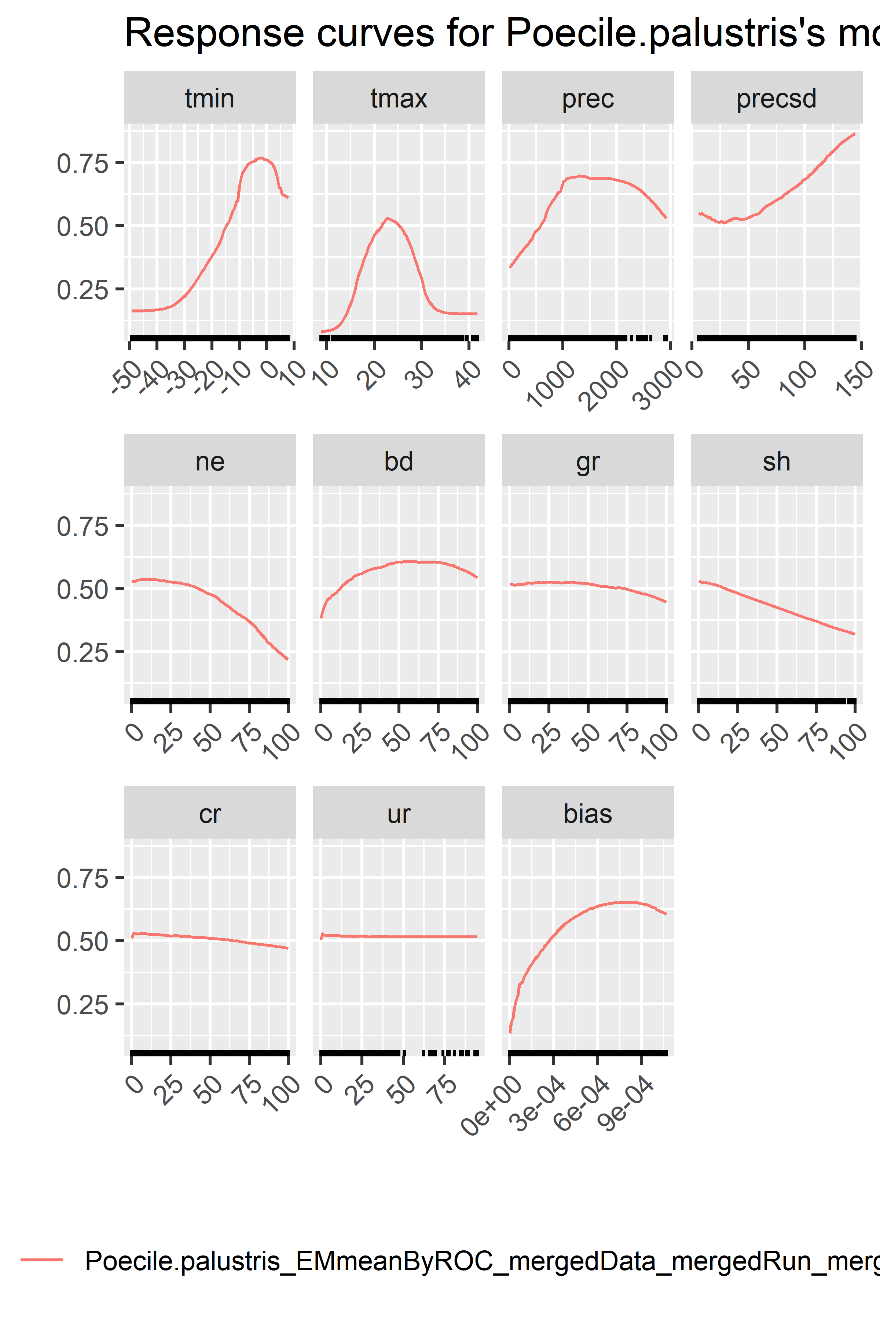


**Figure S23.** Partial response curves for different predictors used in CLIMLAND ensemble models for *Prunella collaris*. Tmax = maximum temperature of the warmest month (ºC); tmin = minimum temperature of the coldest month (ºC), prec = annual precipitation (mm), precsd = precipitation seasonality: ne = needleleaf evergreen forests (%), bd = broadleaf deciduous forests (%), gr = grassland (%), sh = shrubs (%), cr = cops (%), ur = urban (%), bias = sampling bias variable.

**
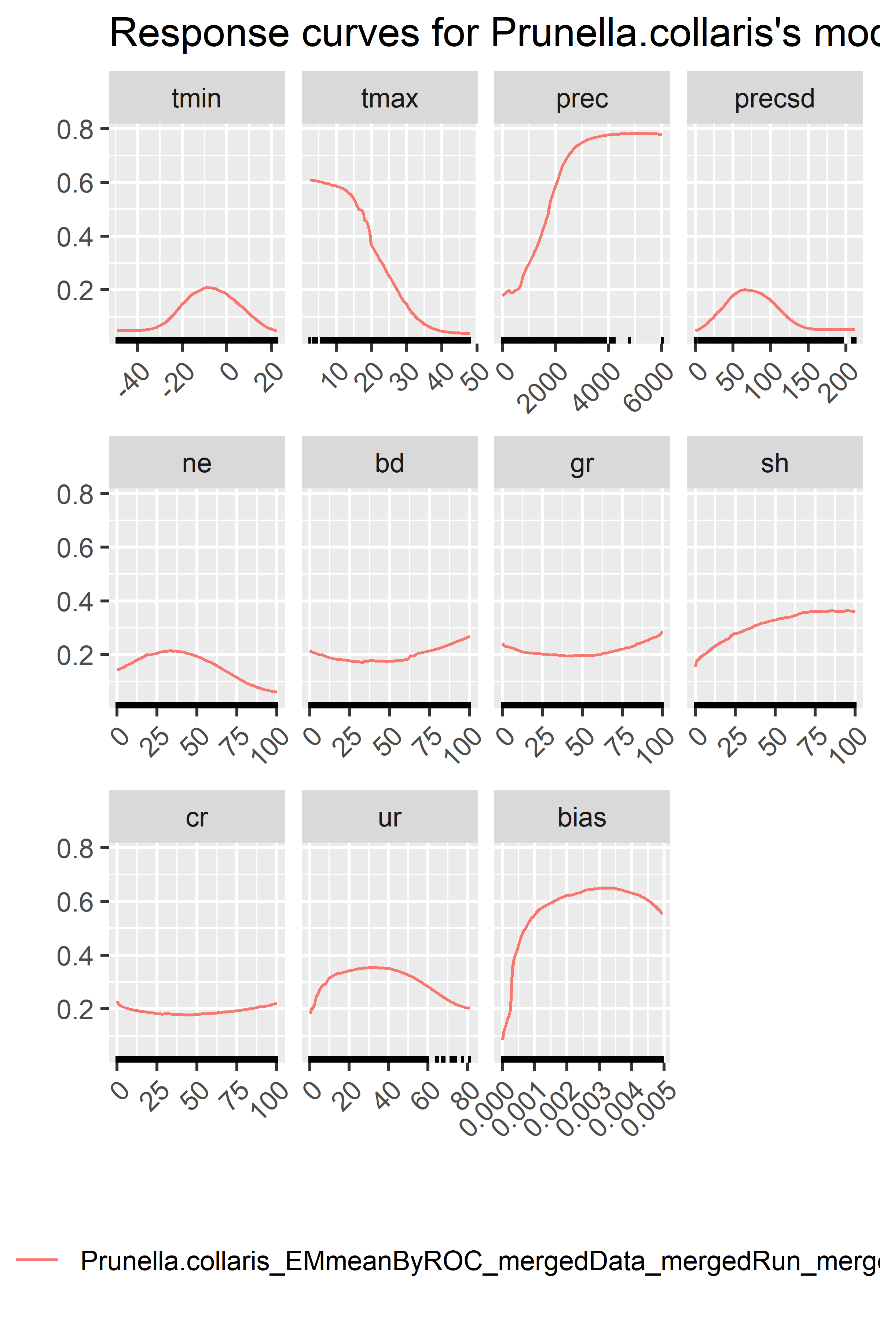
**

**Figure S24.** Partial response curves for different predictors used in CLIMLAND ensemble models for *Prunella modularis*. Tmax = maximum temperature of the warmest month (ºC); tmin = minimum temperature of the coldest month (ºC), prec = annual precipitation (mm), precsd = precipitation seasonality: ne = needleleaf evergreen forests (%), bd = broadleaf deciduous forests (%), gr = grassland (%), sh = shrubs (%), cr = cops (%), ur = urban (%), bias = sampling bias variable.


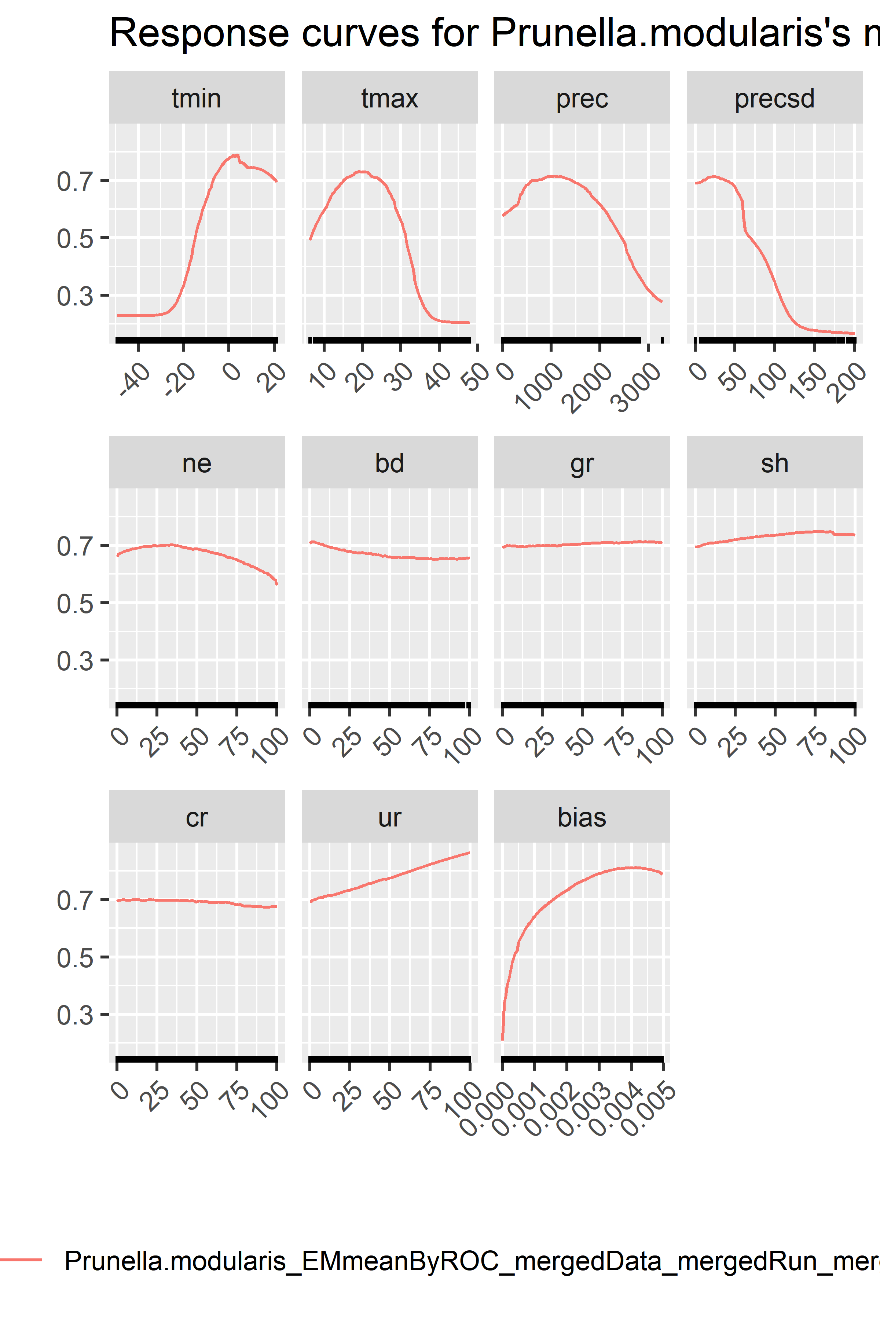


**Figure S25.** Partial response curves for different predictors used in CLIMLAND ensemble models for *Pyrrhocorax graculus*. Tmax = maximum temperature of the warmest month (ºC); tmin = minimum temperature of the coldest month (ºC), prec = annual precipitation (mm), precsd = precipitation seasonality: ne = needleleaf evergreen forests (%), bd = broadleaf deciduous forests (%), gr = grassland (%), sh = shrubs (%), cr = cops (%), ur = urban (%), bias = sampling bias variable.


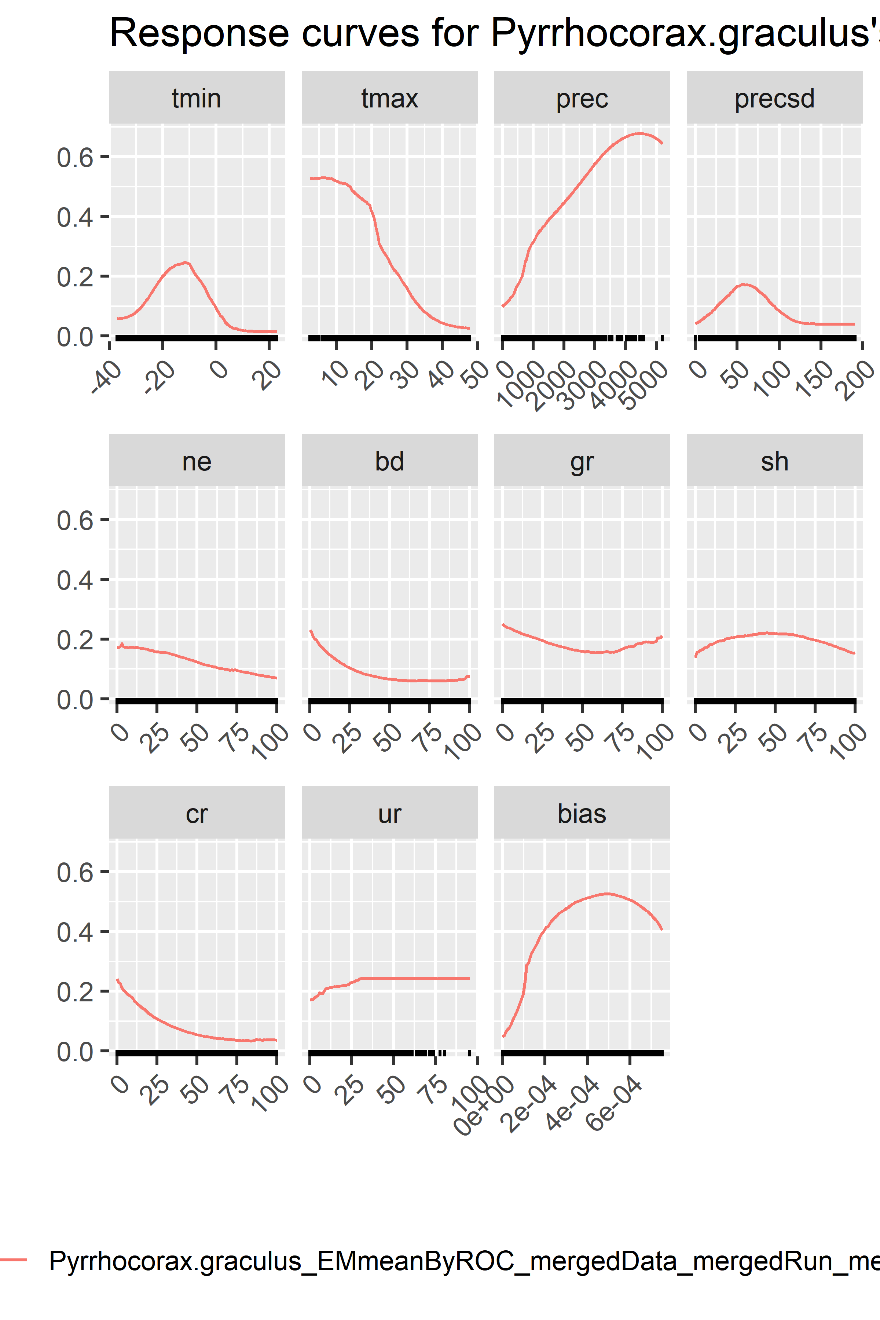


**Figure S26.** Partial response curves for different predictors used in CLIMLAND ensemble models for *Pyrrhula pyrrhula*. Tmax = maximum temperature of the warmest month (ºC); tmin = minimum temperature of the coldest month (ºC), prec = annual precipitation (mm), precsd = precipitation seasonality: ne = needleleaf evergreen forests (%), bd = broadleaf deciduous forests (%), gr = grassland (%), sh = shrubs (%), cr = cops (%), ur = urban (%), bias = sampling bias variable.


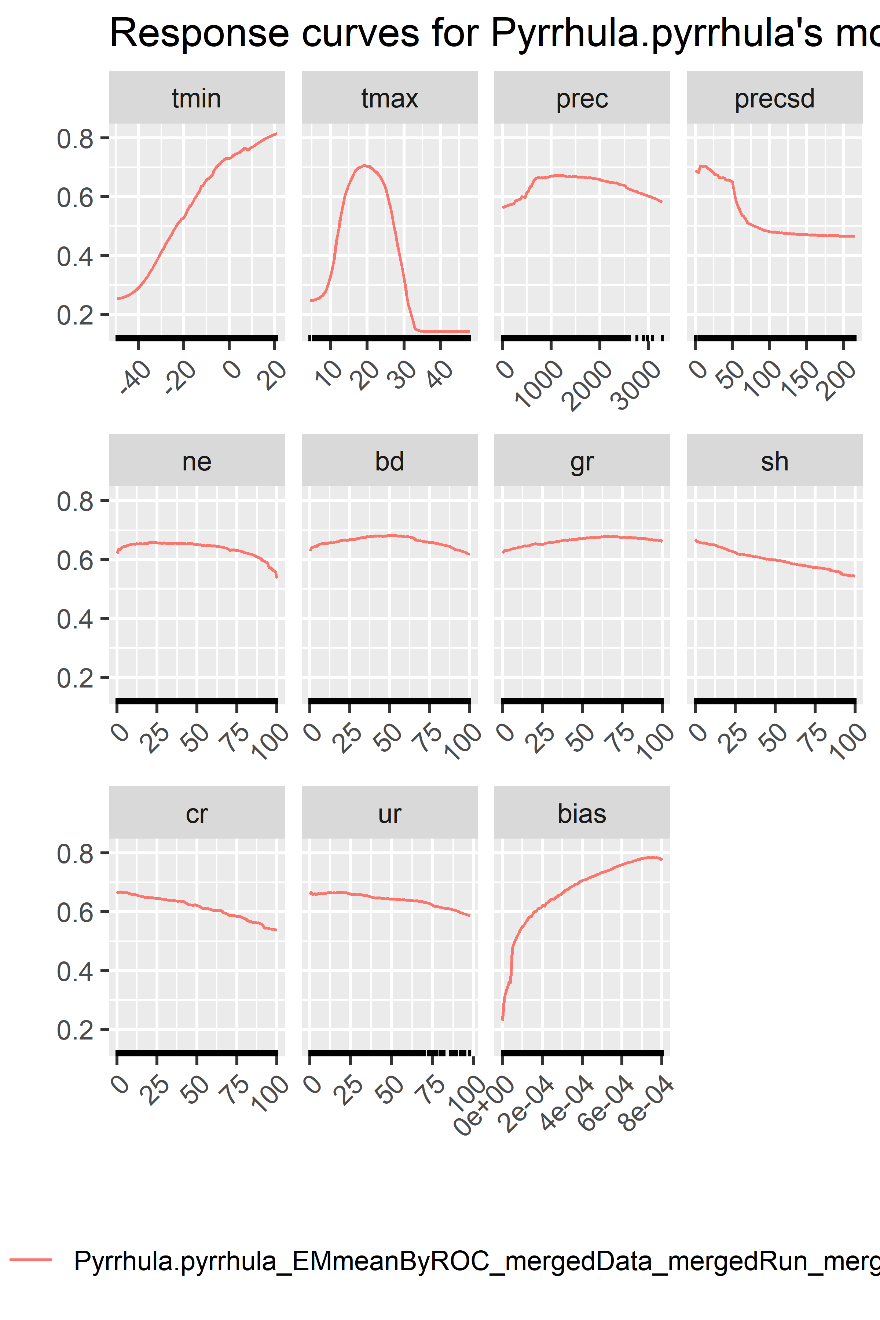


**Figure S27.** Partial response curves for different predictors used in CLIMLAND ensemble models for *Regulus regulus*. Tmax = maximum temperature of the warmest month (ºC); tmin = minimum temperature of the coldest month (ºC), prec = annual precipitation (mm), precsd = precipitation seasonality: ne = needleleaf evergreen forests (%), bd = broadleaf deciduous forests (%), gr = grassland (%), sh = shrubs (%), cr = cops (%), ur = urban (%), bias = sampling bias variable.


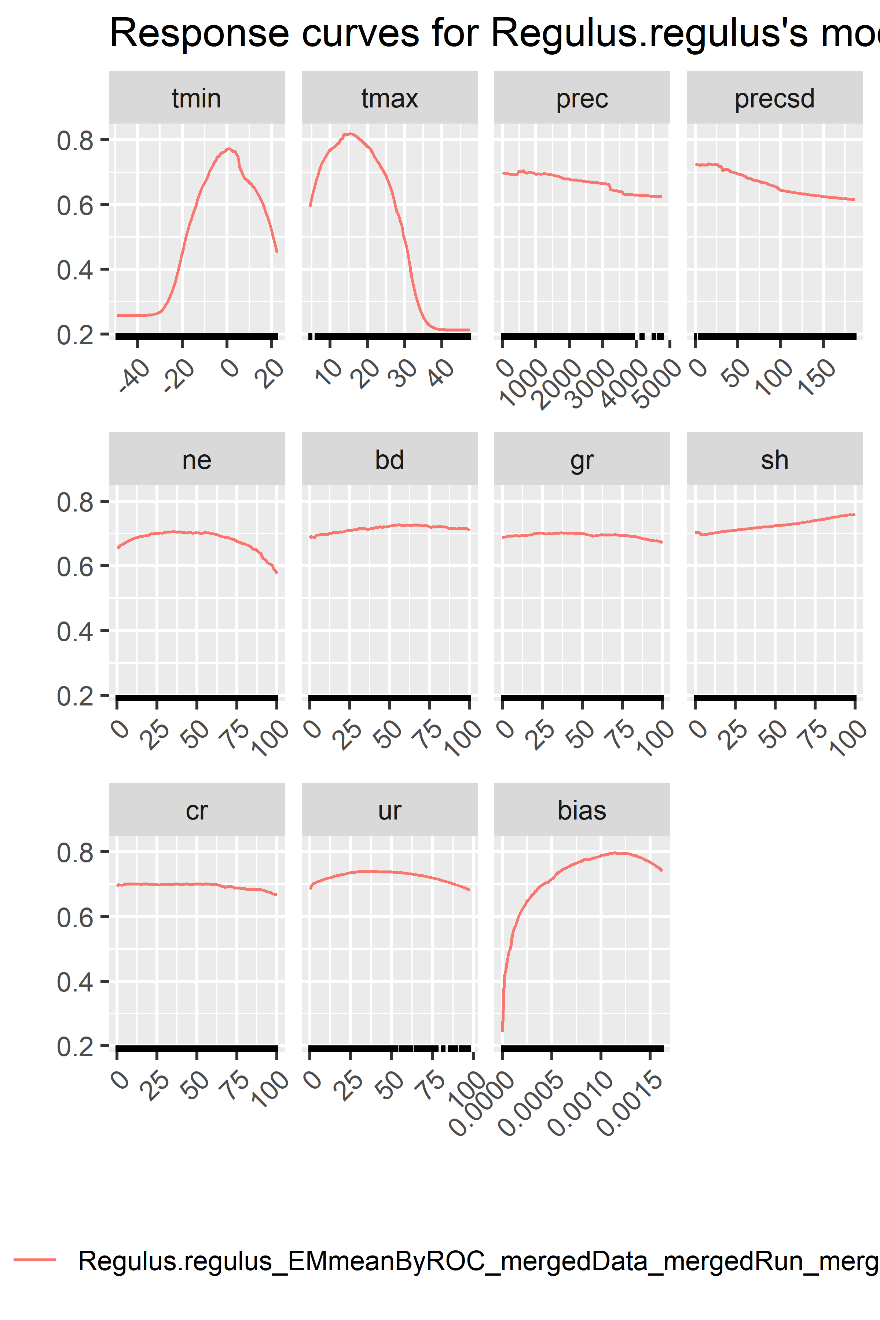


**Figure S28.** Partial response curves for different predictors used in CLIMLAND ensemble models for *Scolopax rusticola*. Tmax = maximum temperature of the warmest month (ºC); tmin = minimum temperature of the coldest month (ºC), prec = annual precipitation (mm), precsd = precipitation seasonality: ne = needleleaf evergreen forests (%), bd = broadleaf deciduous forests (%), gr = grassland (%), sh = shrubs (%), cr = cops (%), ur = urban (%), bias = sampling bias variable.


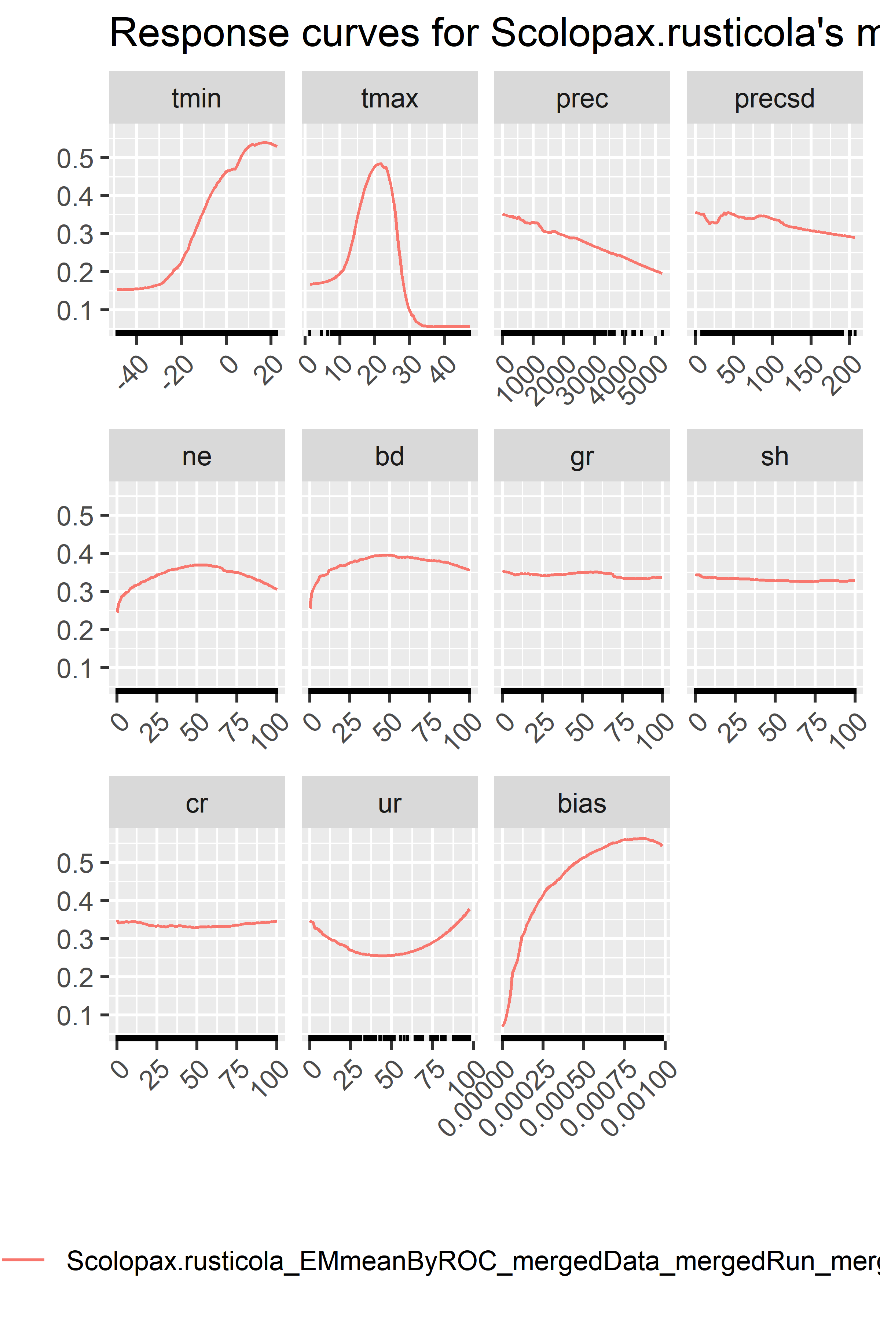


**Figure S29.** Partial response curves for different predictors used in CLIMLAND ensemble models for *Sylvia borin*. Tmax = maximum temperature of the warmest month (ºC); tmin = minimum temperature of the coldest month (ºC), prec = annual precipitation (mm), precsd = precipitation seasonality: ne = needleleaf evergreen forests (%), bd = broadleaf deciduous forests (%), gr = grassland (%), sh = shrubs (%), cr = cops (%), ur = urban (%), bias = sampling bias variable.


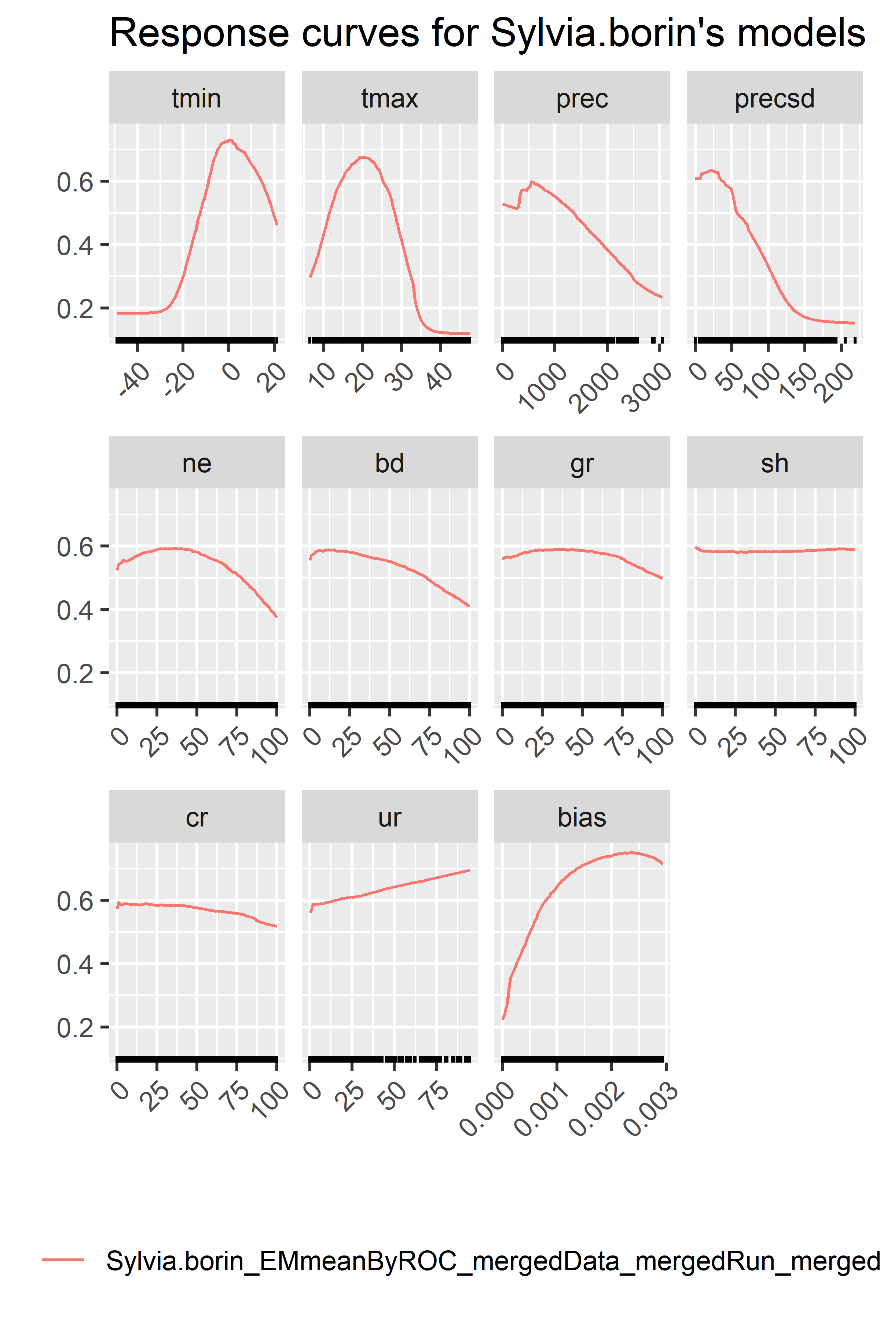


**Figure S30.** Partial response curves for different predictors used in CLIMLAND ensemble models for *Tetrao urogallus*. Tmax = maximum temperature of the warmest month (ºC); tmin = minimum temperature of the coldest month (ºC), prec = annual precipitation (mm), precsd = precipitation seasonality: ne = needleleaf evergreen forests (%), bd = broadleaf deciduous forests (%), gr = grassland (%), sh = shrubs (%), cr = cops (%), ur = urban (%), bias = sampling bias variable.


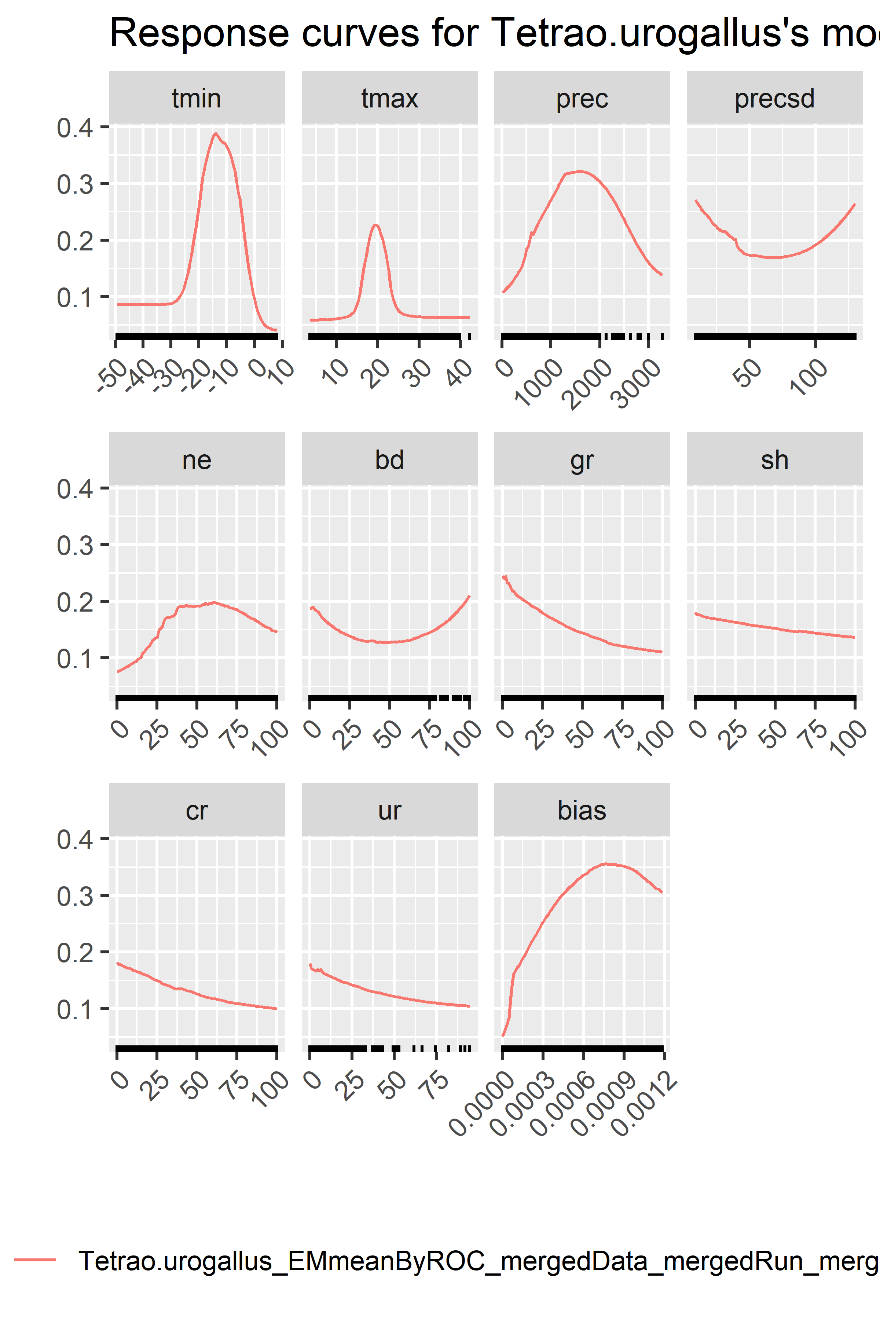


**Figure S31.** Partial response curves for different predictors used in CLIMLAND ensemble models for *Tichodroma muraria*. Tmax = maximum temperature of the warmest month (ºC); tmin = minimum temperature of the coldest month (ºC), prec = annual precipitation (mm), precsd = precipitation seasonality: ne = needleleaf evergreen forests (%), bd = broadleaf deciduous forests (%), gr = grassland (%), sh = shrubs (%), cr = cops (%), ur = urban (%), bias = sampling bias variable.

**
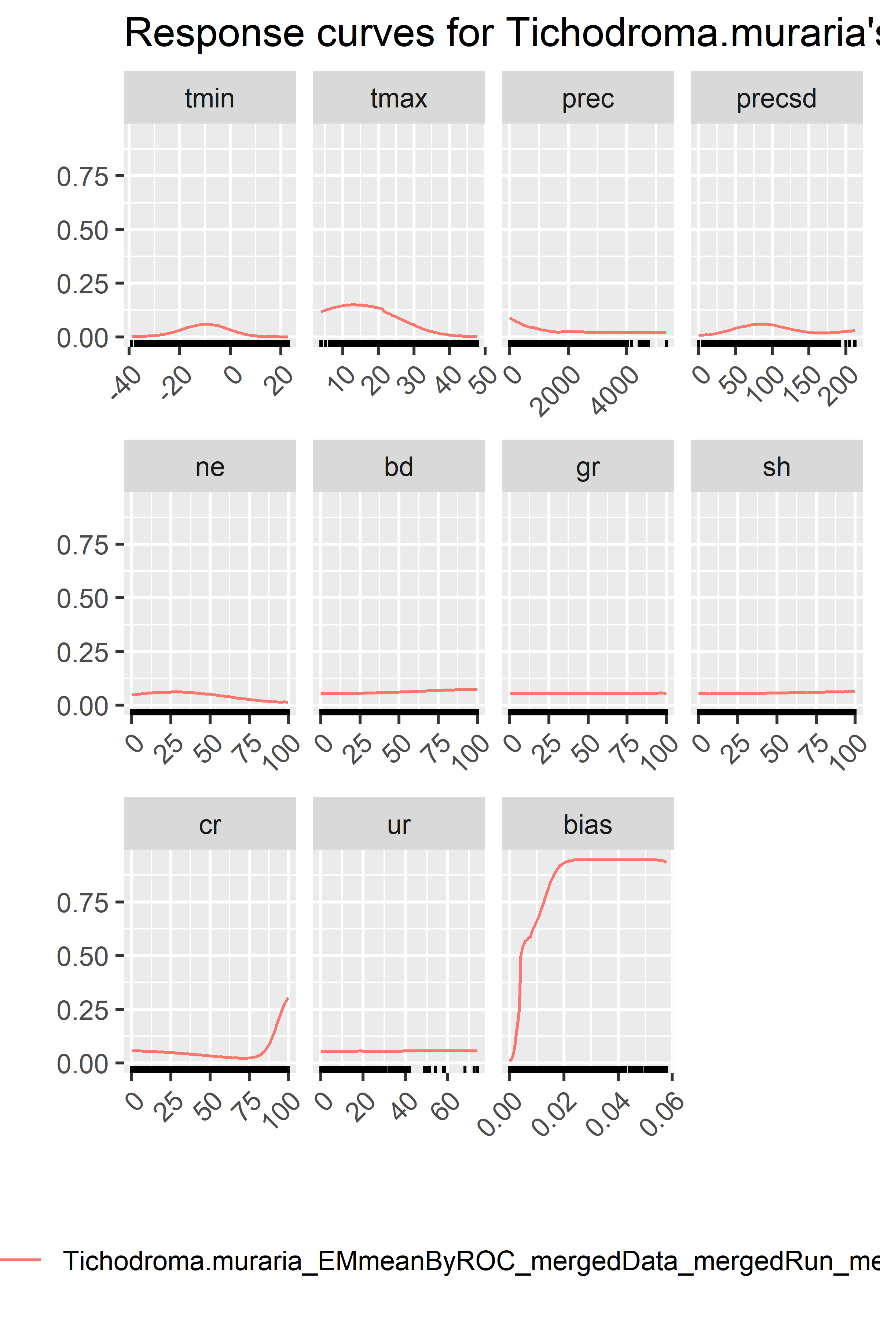
**

**Figure S32.** Partial response curves for different predictors used in CLIMLAND ensemble models for *Turdus philomelos*. Tmax = maximum temperature of the warmest month (ºC); tmin = minimum temperature of the coldest month (ºC), prec = annual precipitation (mm), precsd = precipitation seasonality: ne = needleleaf evergreen forests (%), bd = broadleaf deciduous forests (%), gr = grassland (%), sh = shrubs (%), cr = cops (%), ur = urban (%), bias = sampling bias variable.


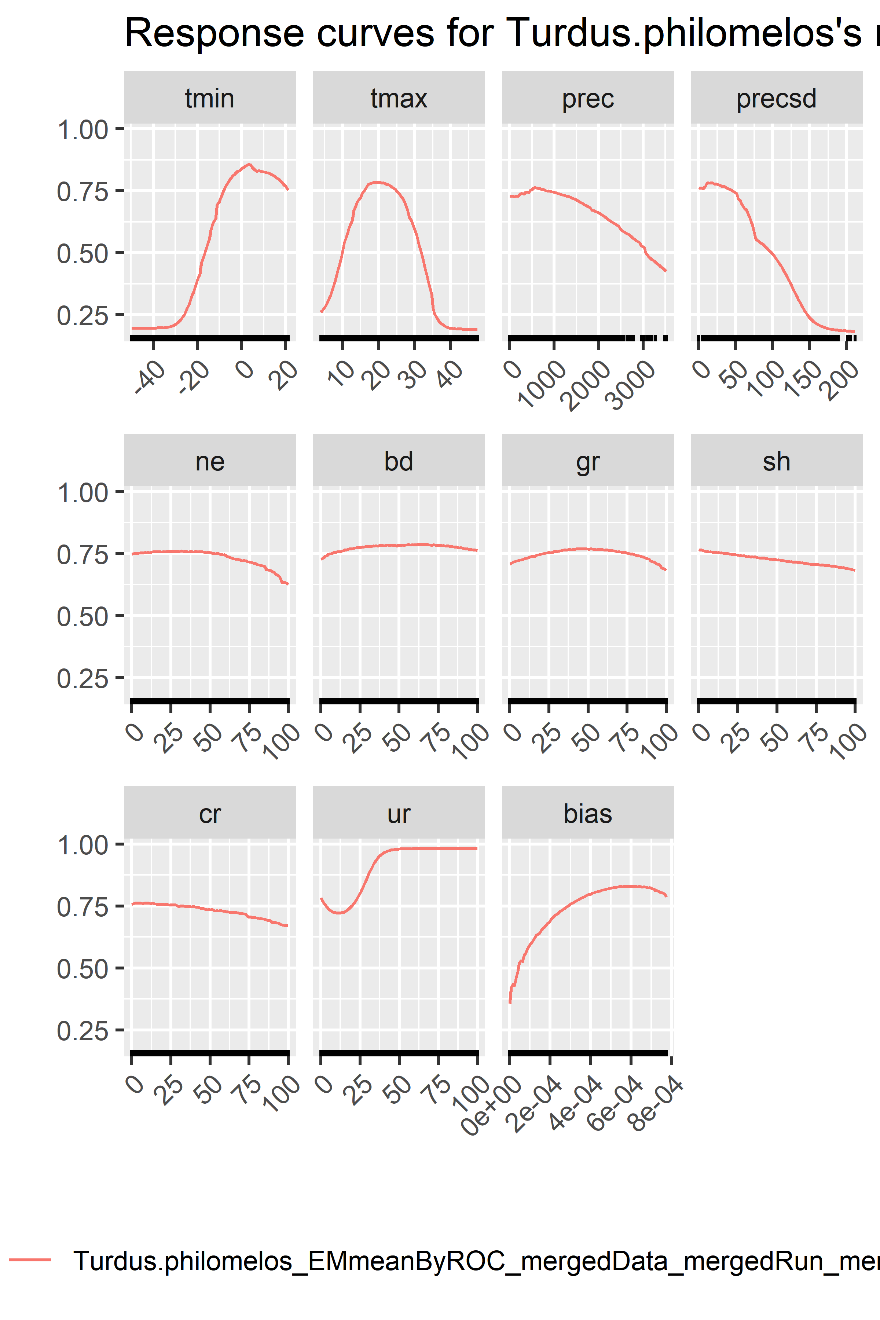


**Figure S33.** Partial response curves for different predictors used in CLIMLAND ensemble models for *Turdus torquatus*. Tmax = maximum temperature of the warmest month (ºC); tmin = minimum temperature of the coldest month (ºC), prec = annual precipitation (mm), precsd = precipitation seasonality: ne = needleleaf evergreen forests (%), bd = broadleaf deciduous forests (%), gr = grassland (%), sh = shrubs (%), cr = cops (%), ur = urban (%), bias = sampling bias variable.


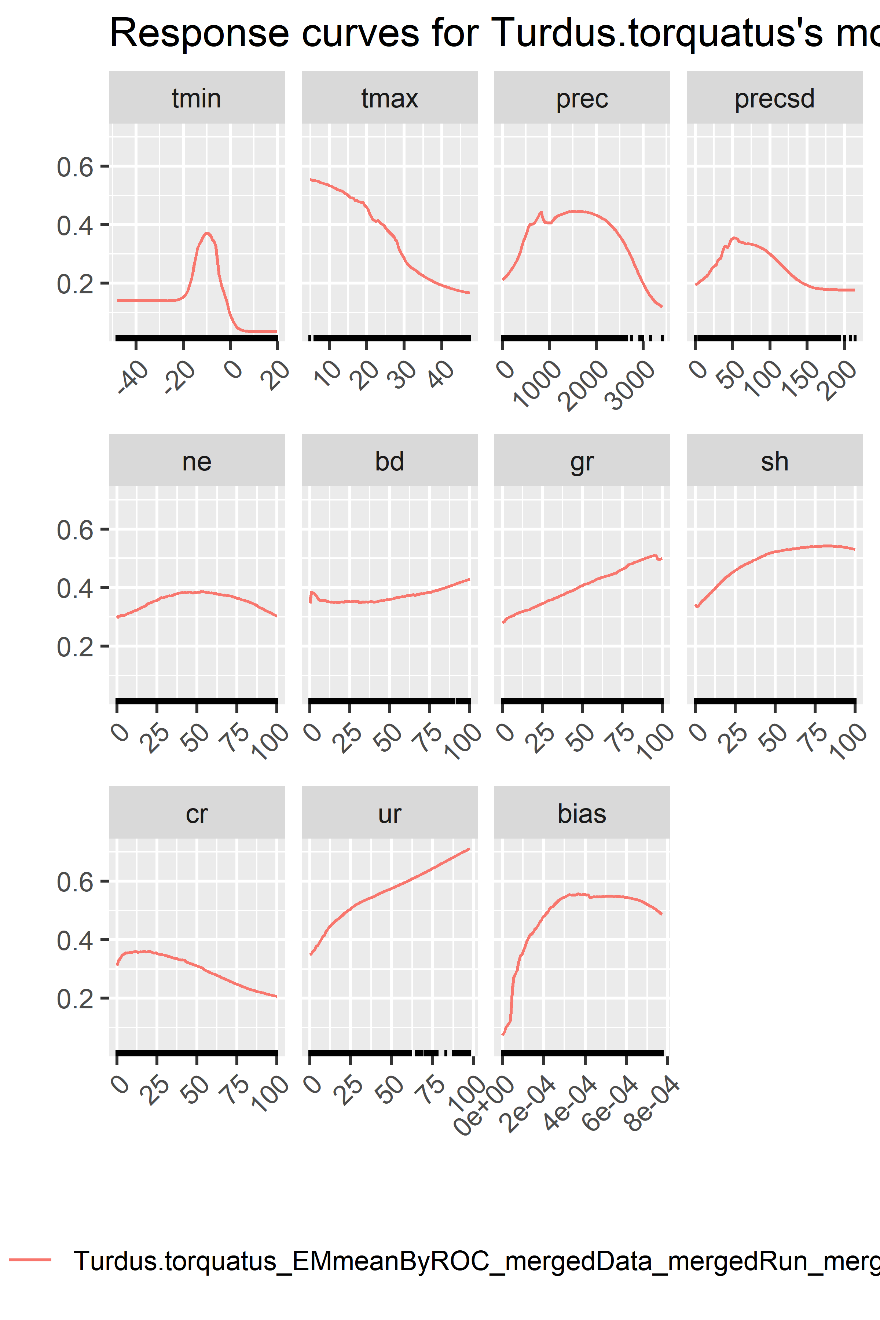


**Figure S34.** Partial response curves for different predictors used in CLIMLAND ensemble models for *Perdix perdix*. Tmax = maximum temperature of the warmest month (ºC); tmin = minimum temperature of the coldest month (ºC), prec = annual precipitation (mm), precsd = precipitation seasonality: ne = needleleaf evergreen forests (%), bd = broadleaf deciduous forests (%), gr = grassland (%), sh = shrubs (%), cr = cops (%), ur = urban (%), bias = sampling bias variable.


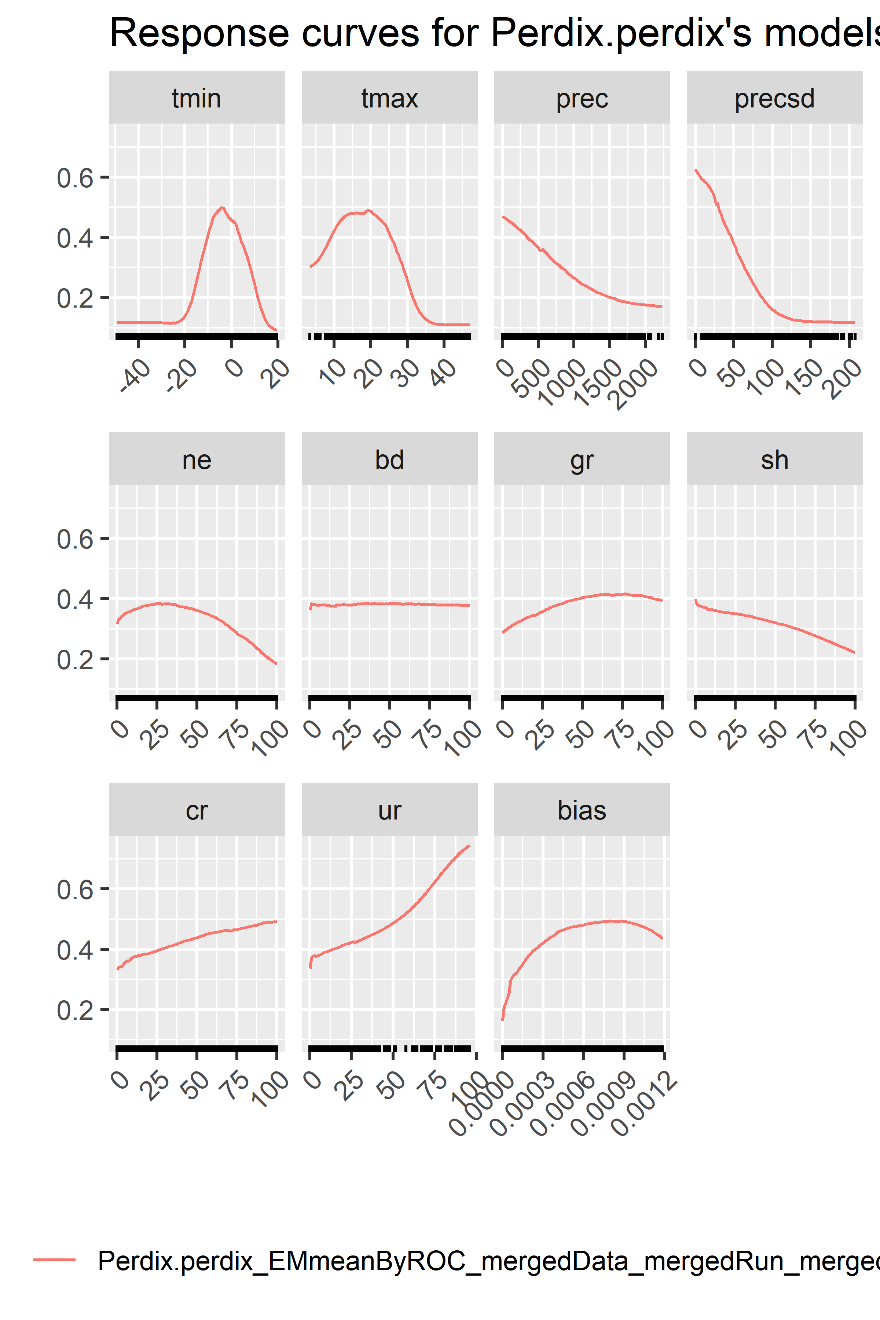


**Figure S35.** Partial response curves for different predictors used in CLIMLAND ensemble models for *Saxicola rubetra*. Tmax = maximum temperature of the warmest month (ºC); tmin = minimum temperature of the coldest month (ºC), prec = annual precipitation (mm), precsd = precipitation seasonality: ne = needleleaf evergreen forests (%), bd = broadleaf deciduous forests (%), gr = grassland (%), sh = shrubs (%), cr = cops (%), ur = urban (%), bias = sampling bias variable.

**
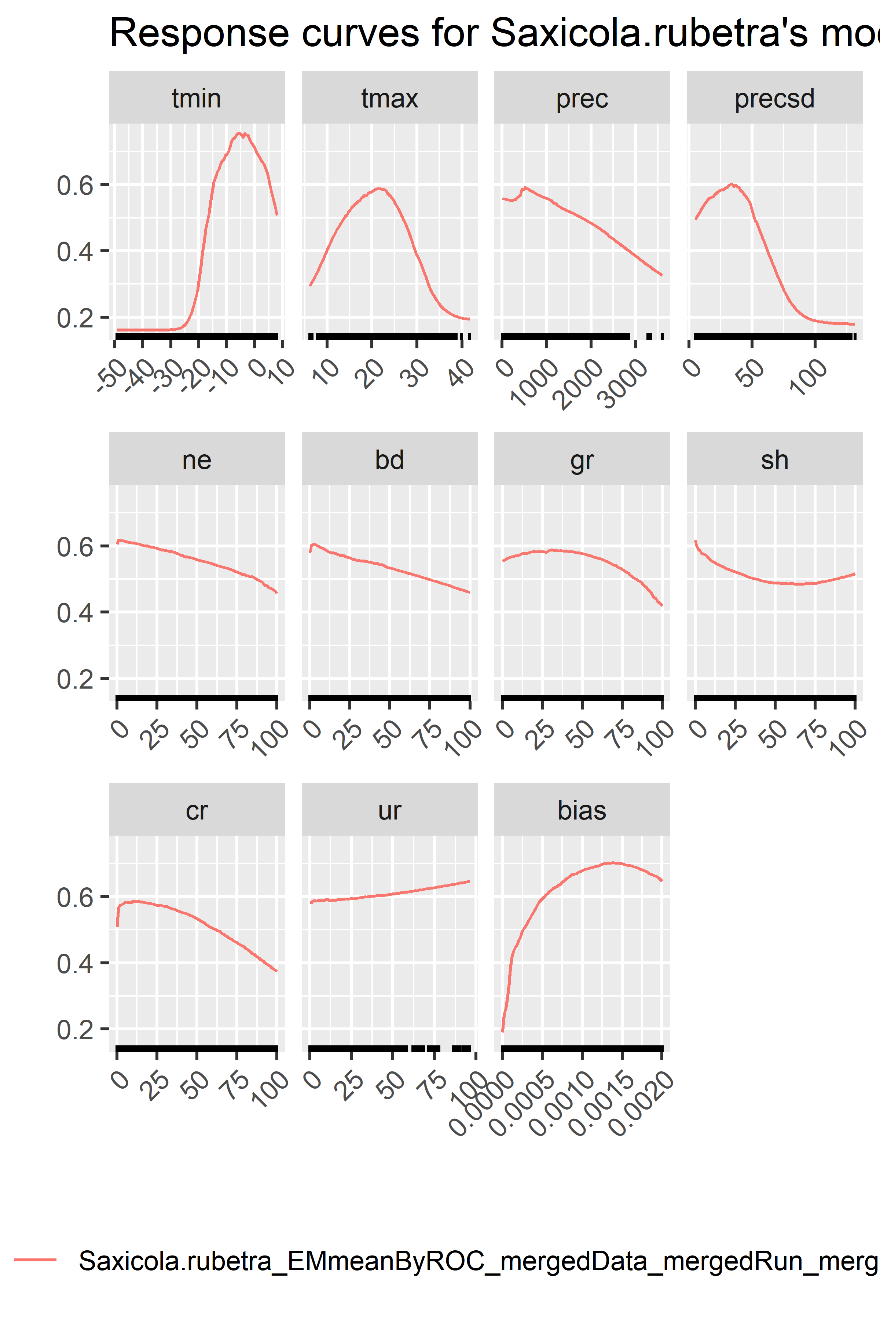
**


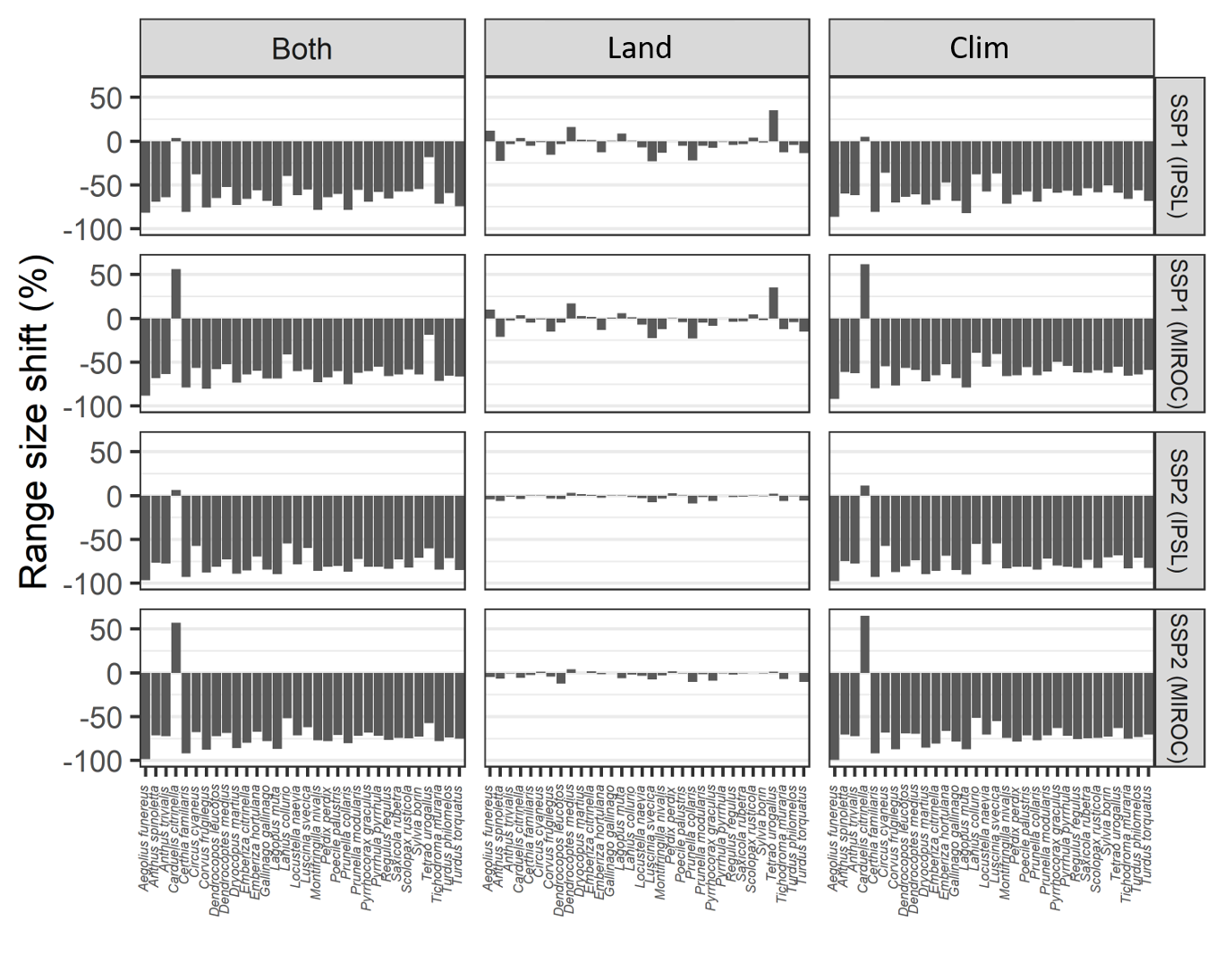
**Figure S36.** Predicted shifts in the extent of species ranges under different scenarios when both climate and land cover variables (both), only climate (clim) or only land cover (land) are assumed to change for CLIMLAND model predictions. Shifts in species range sizes are expressed as the percentage change with respect to the extent of current suitable habitat. N = 32.

**Figure S37.** Predicted range shifts under different scenarios when both climate and land cover (both), only climate (clim) or only land cover (land) are assumed to change in CLIMLAND model predictions. Arrows represent the displacement of the centroid of suitable habitat between the present and future scenarios. Shift of different species whose range is predicted to decrease (black) or increase (red) and average shift across all them (blue) are shown.


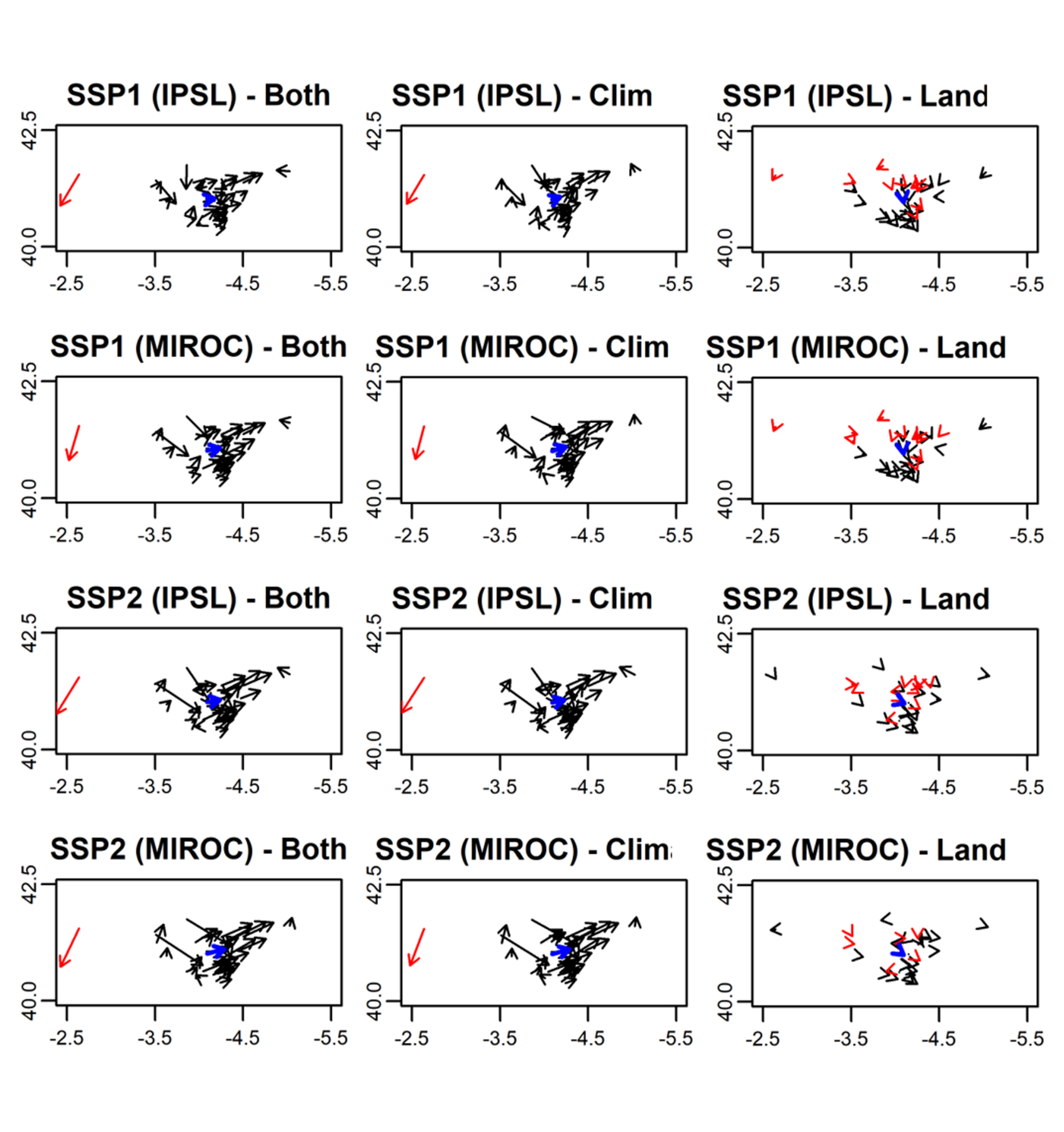


**Figure S38.** Cumulative map predictions of suitable habitat for different species under different scenarios when both climate and land cover (land), only climate (clim) or only land cover (land) are assumed to change in CLIMLAND model predictions.


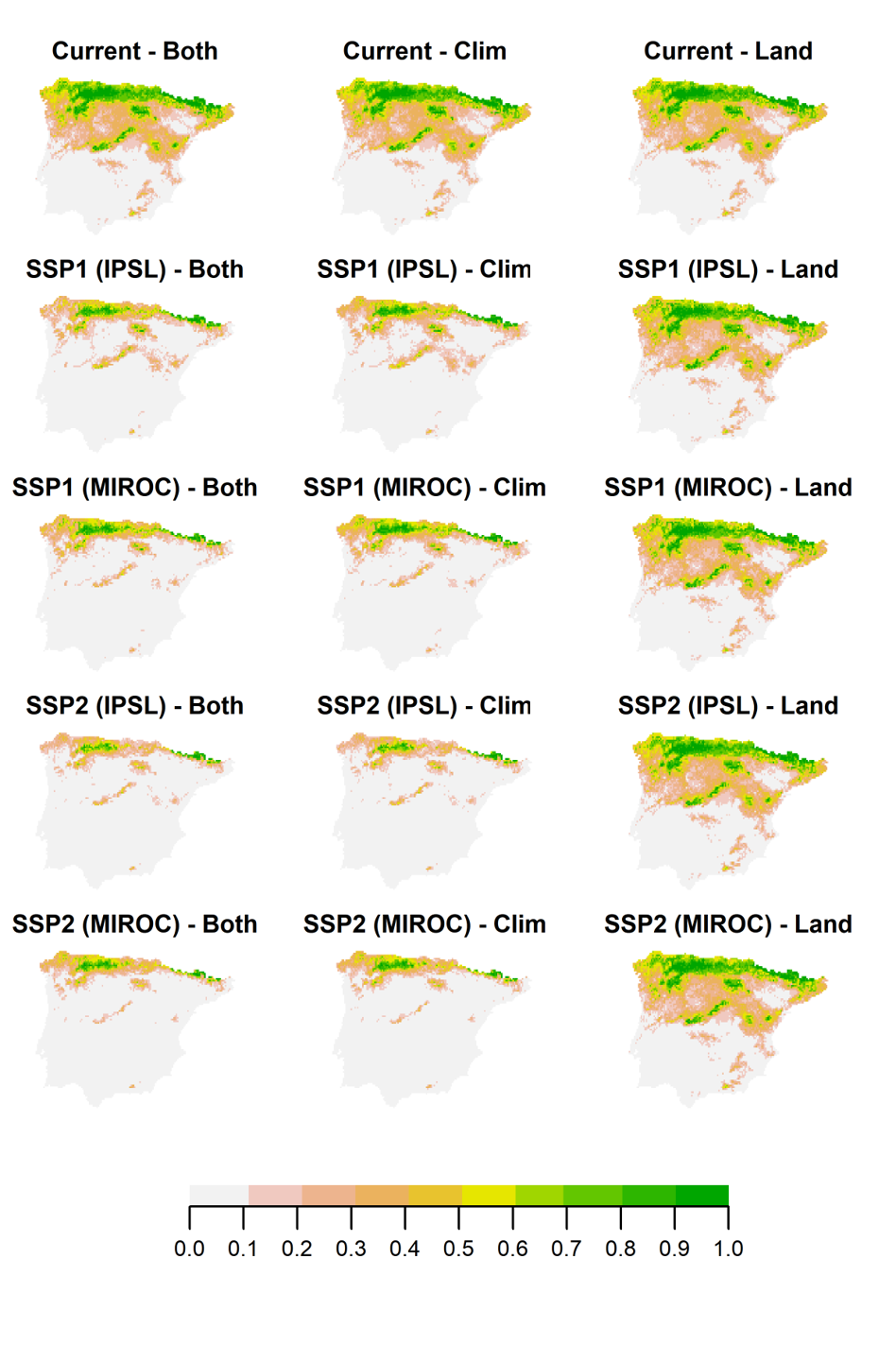


**Figure S39.** Map predictions of suitable habitat for *Aegolius funereus*. Continuous predictions (graduated colors) and binary map predictions (polygon) are shown.


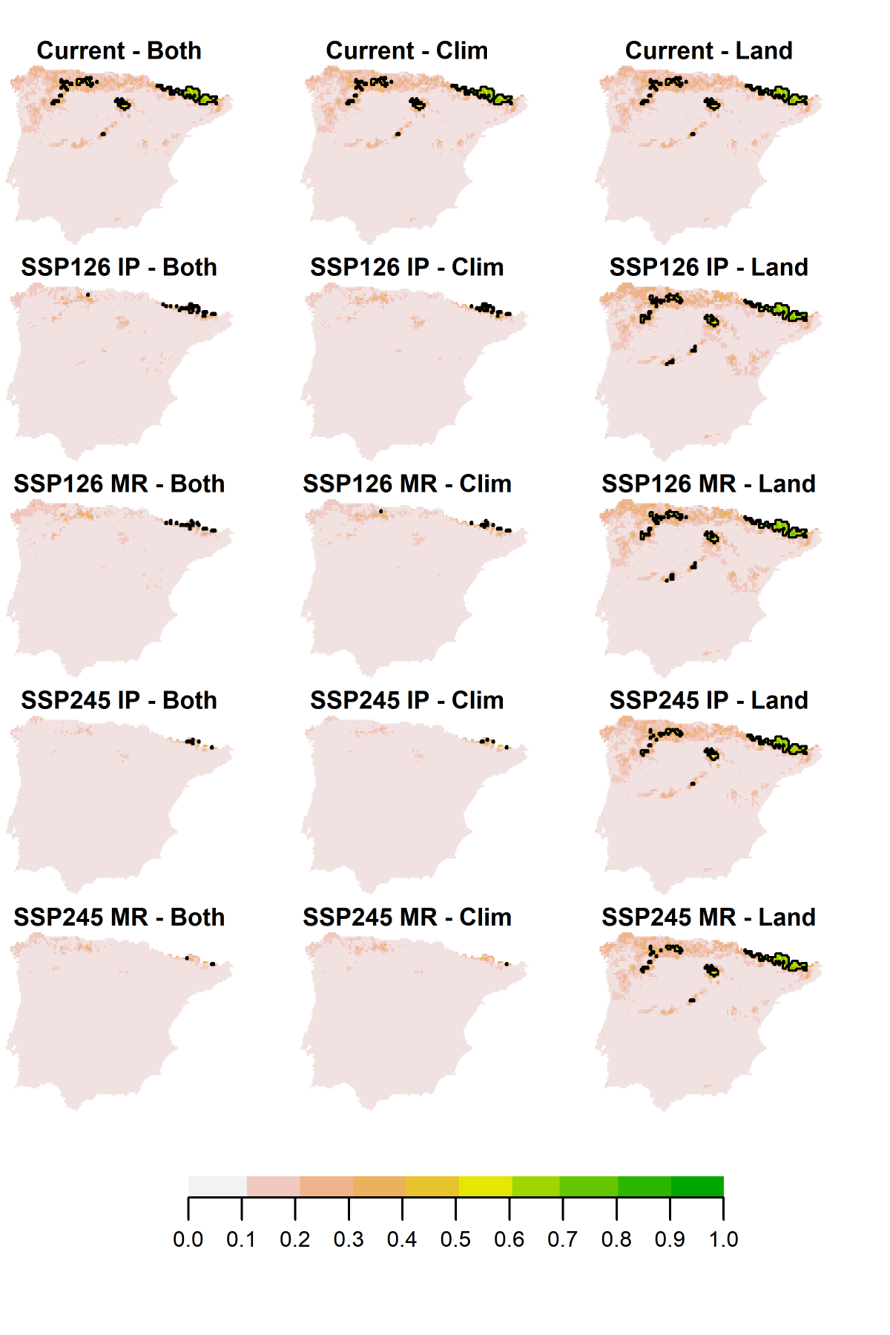


**Figure S40.** Map predictions of suitable habitat for *Anthus spinoletta*. Continuous predictions (graduated colors) and binary map predictions (polygon) are shown.


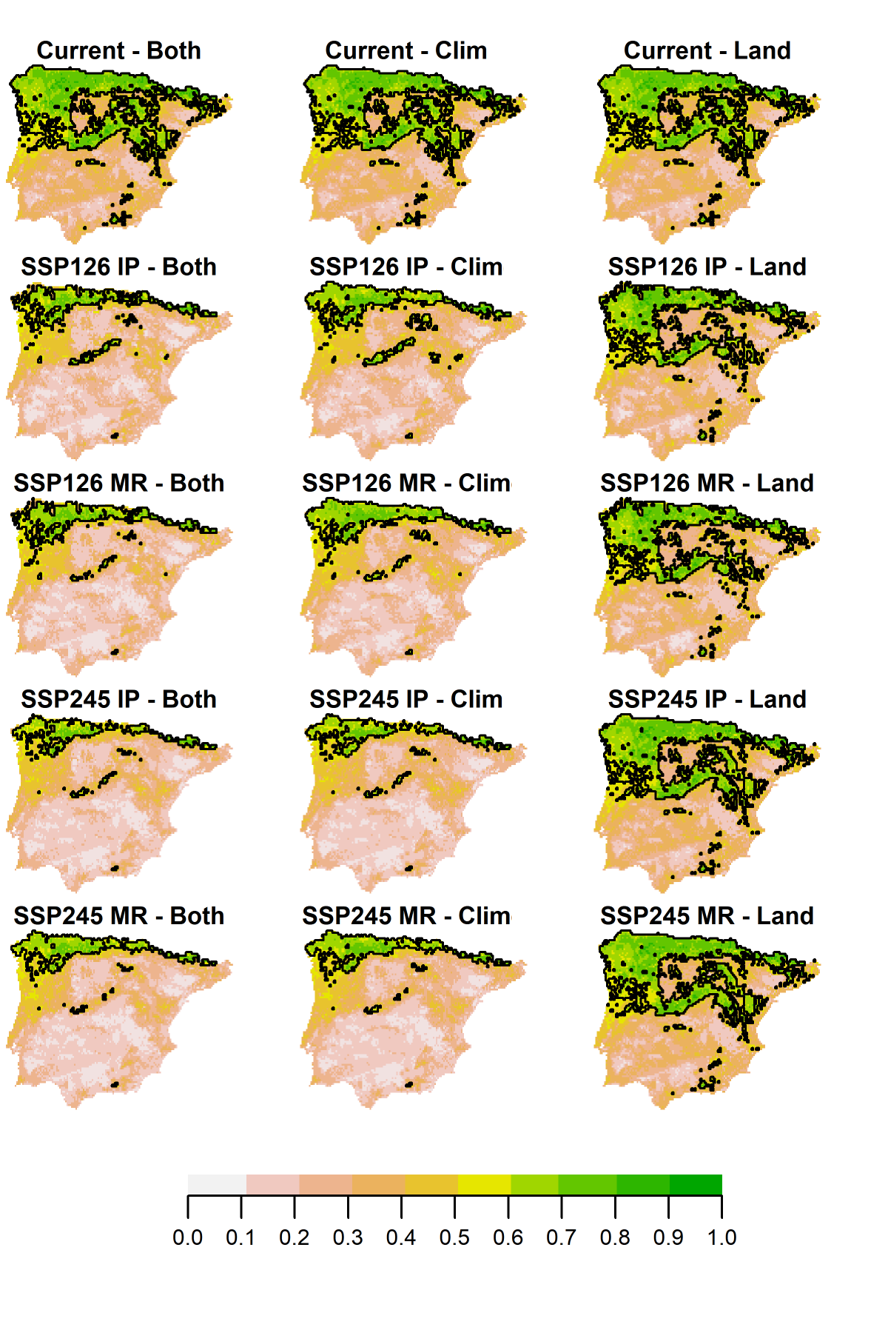


**Figure S41.** Map predictions of suitable habitat for *Anthus trivialis*. Continuous predictions (graduated colors) and binary map predictions (polygon) are shown.


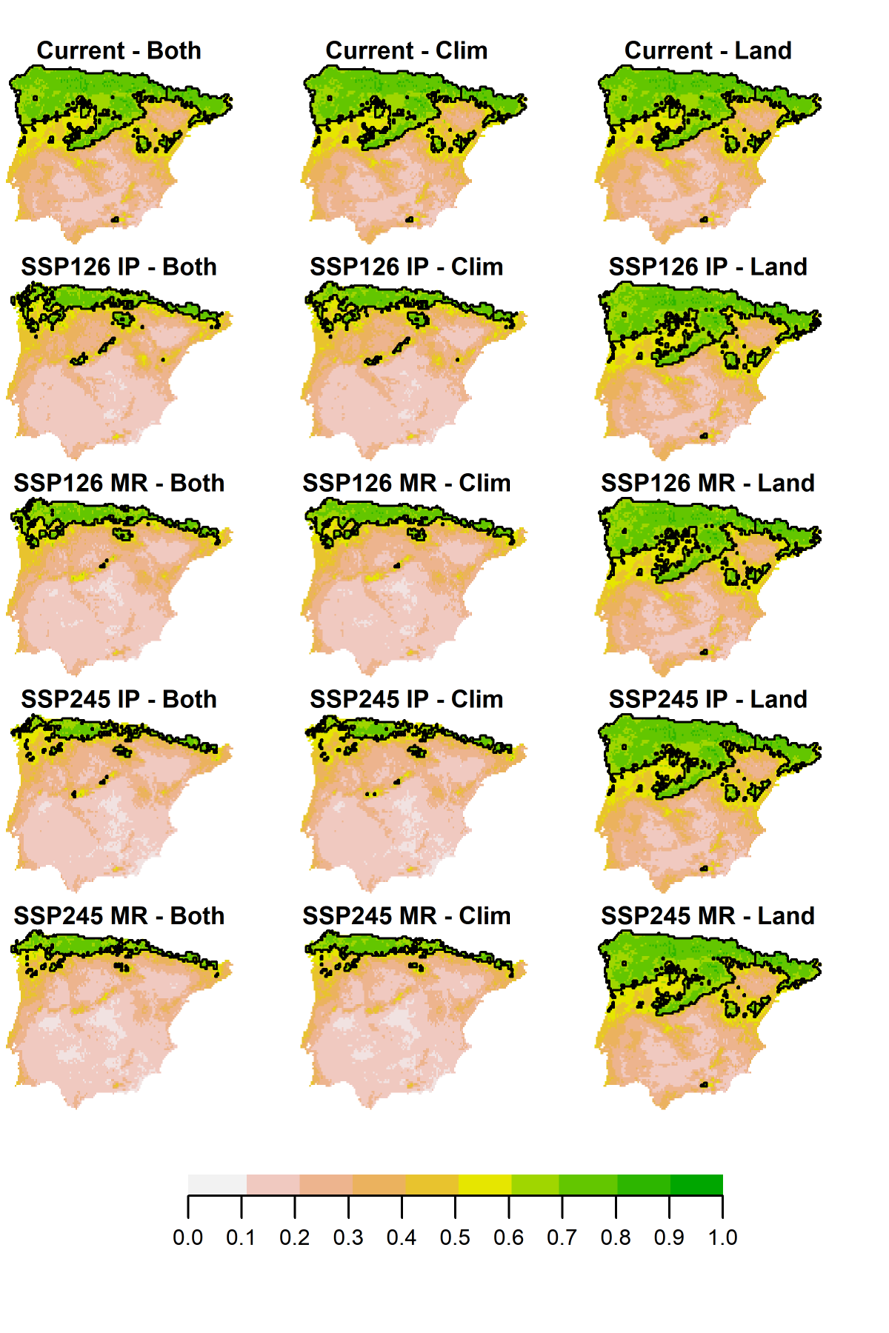


**Figure S42.** Map predictions of suitable habitat for *Carduelis citrinella.* Continuous predictions (graduated colors) and binary map predictions (polygon) are shown.

*
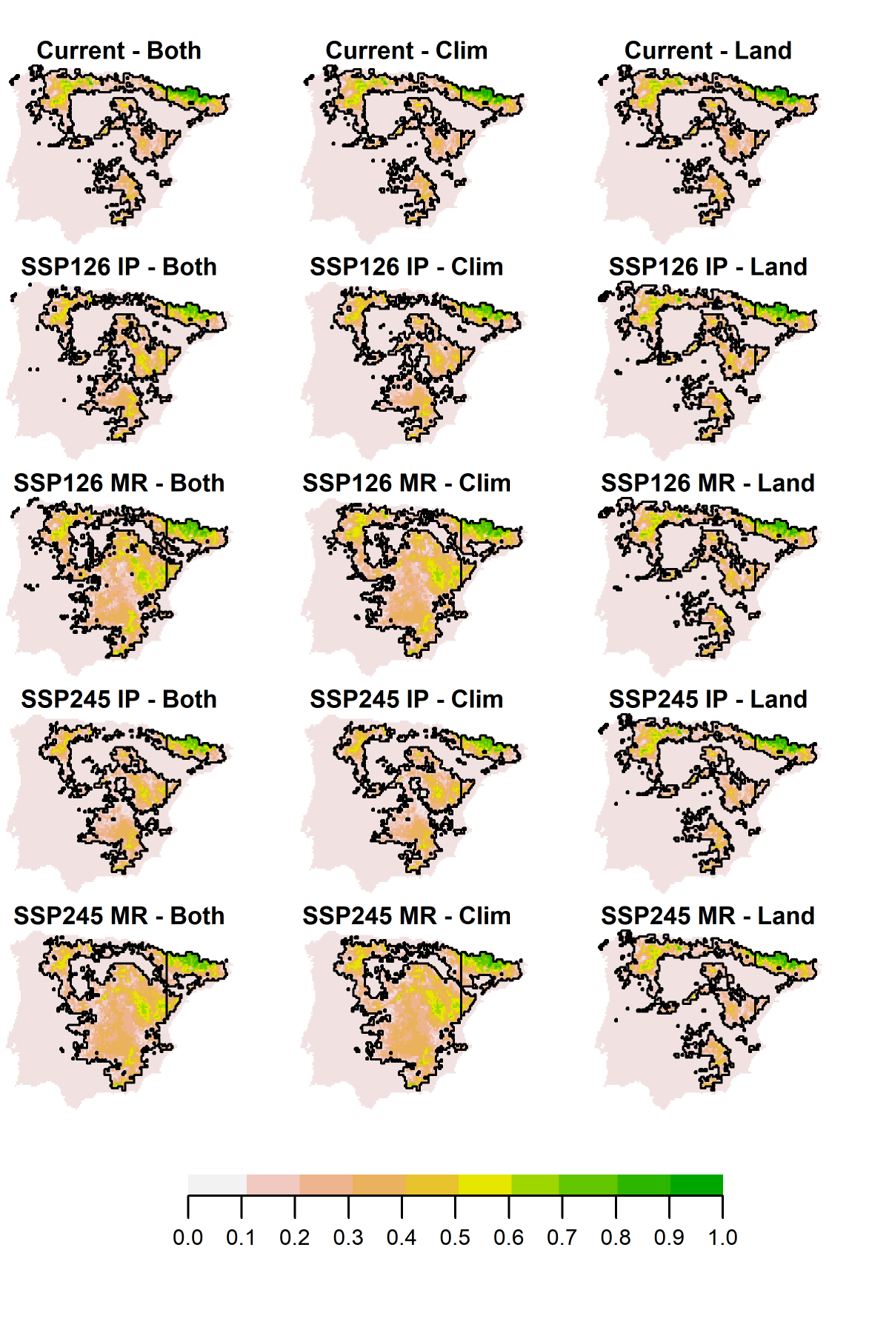
*

**Figure S43.** Map predictions of suitable habitat for *Certhia familiaris.* Continuous predictions (graduated colors) and binary map predictions (polygon) are shown.


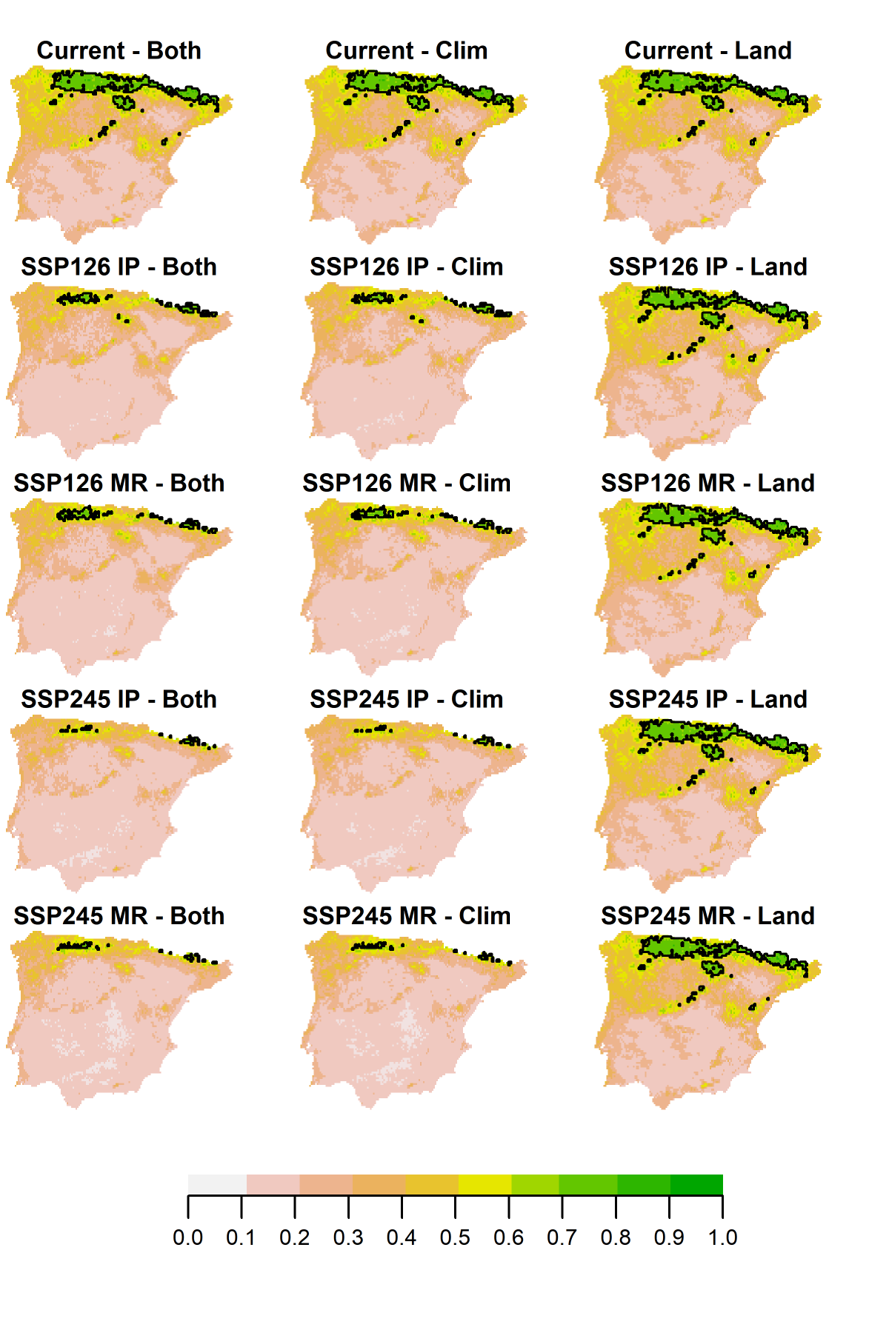


**Figure S44.** Map predictions of suitable habitat for *Circus cyaneus.* Continuous predictions (graduated colors) and binary map predictions (polygon) are shown.

*
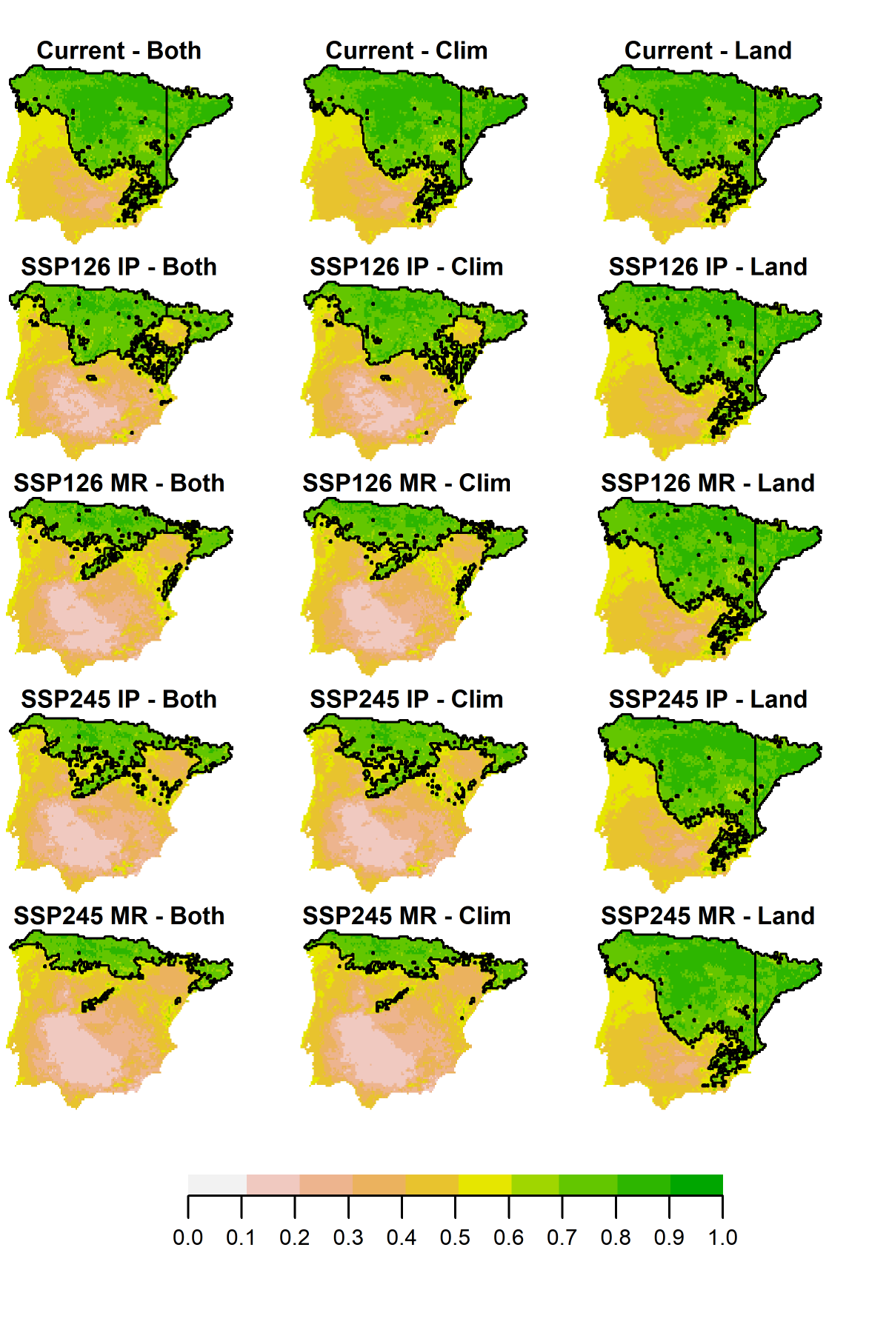
*

**Figure S45.** Map predictions of suitable habitat for *Corvus frugilegus.* Continuous predictions (graduated colors) and binary map predictions (polygon) are shown.

*
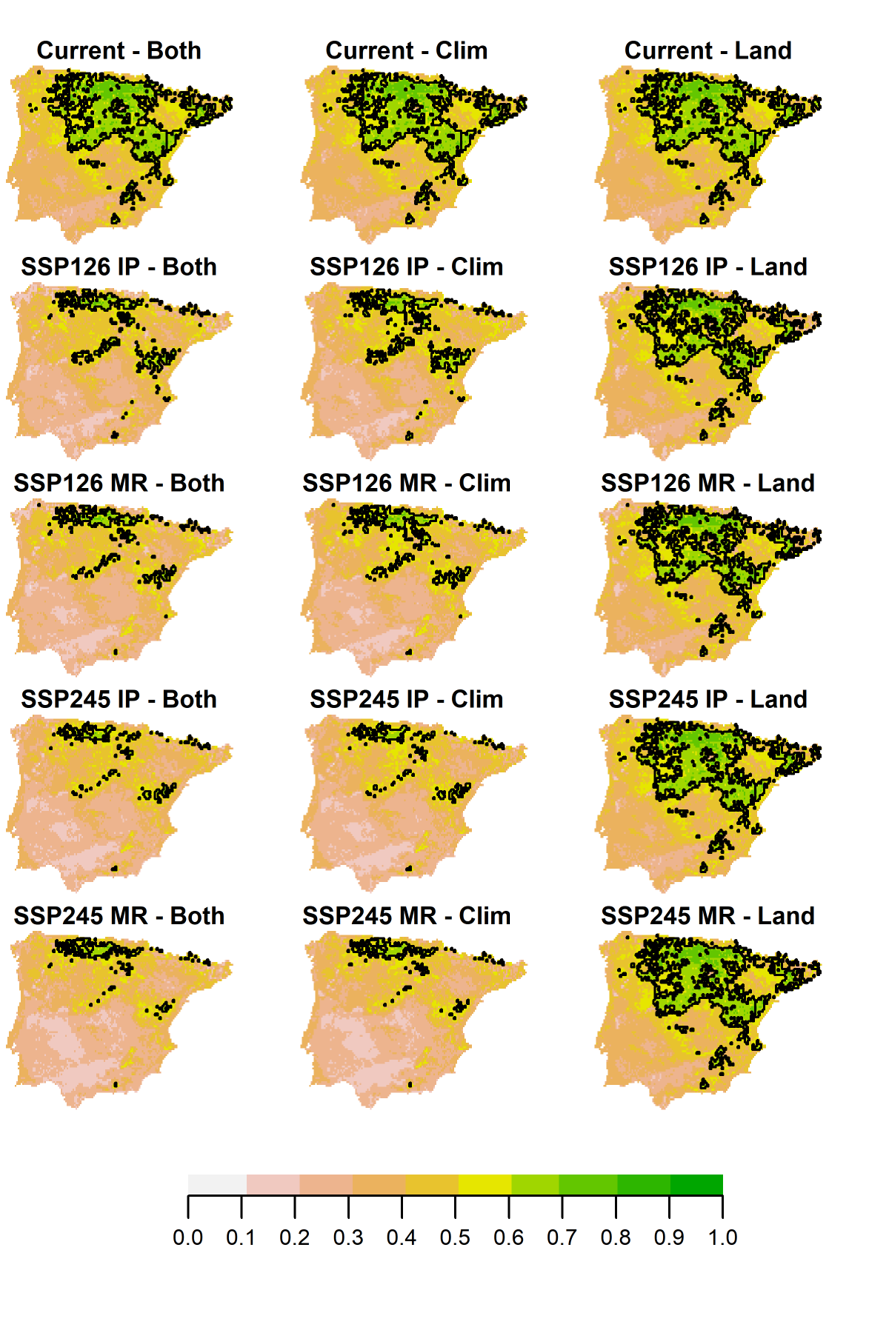
*

**Figure S46.** Map predictions of suitable habitat for *Dendrocopos leucotos.* Continuous predictions (graduated colors) and binary map predictions (polygon) are shown.

*
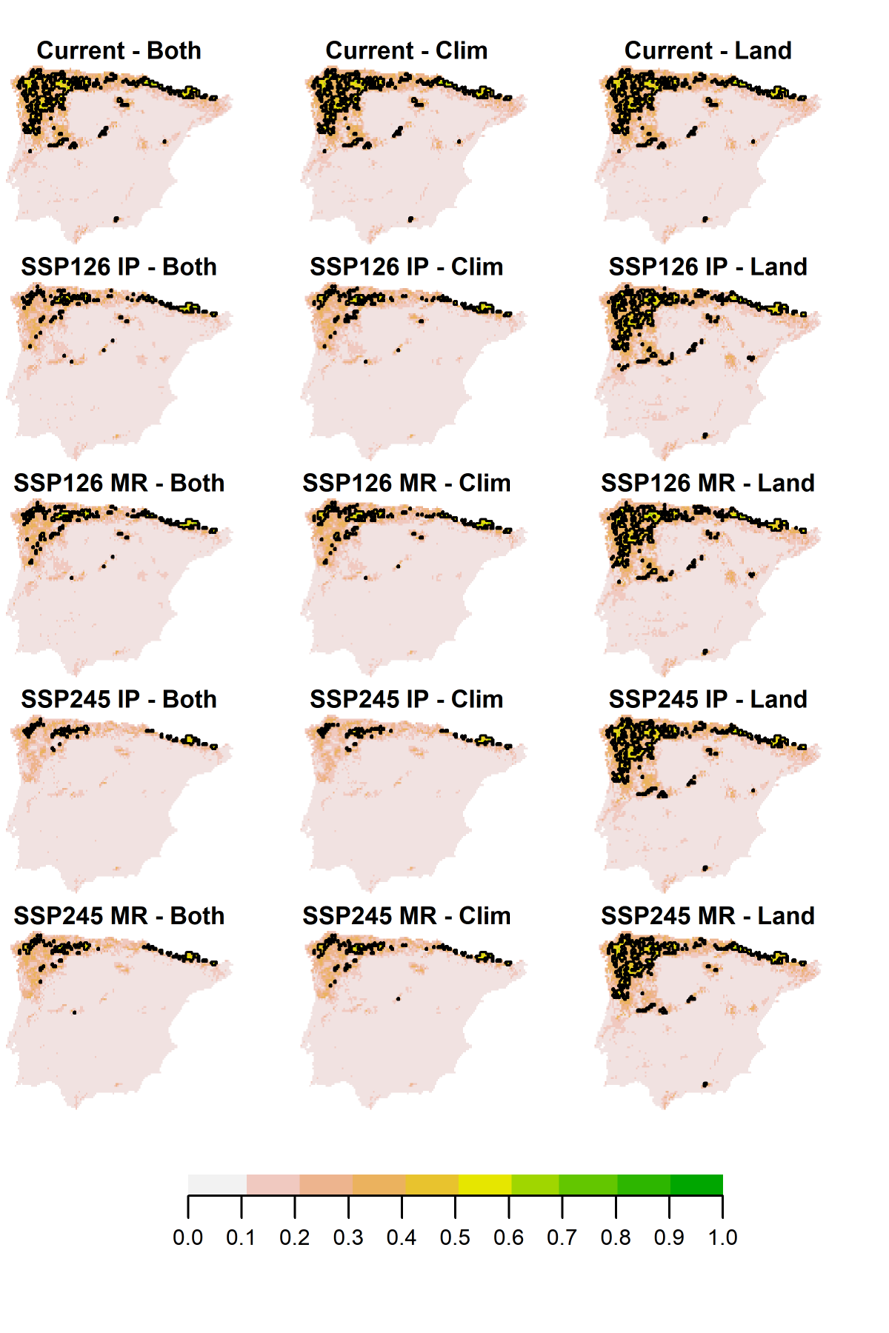
*

**Figure S47.** Map predictions of suitable habitat for *Dendrocoptes medius.* Continuous predictions (graduated colors) and binary map predictions (polygon) are shown.

*
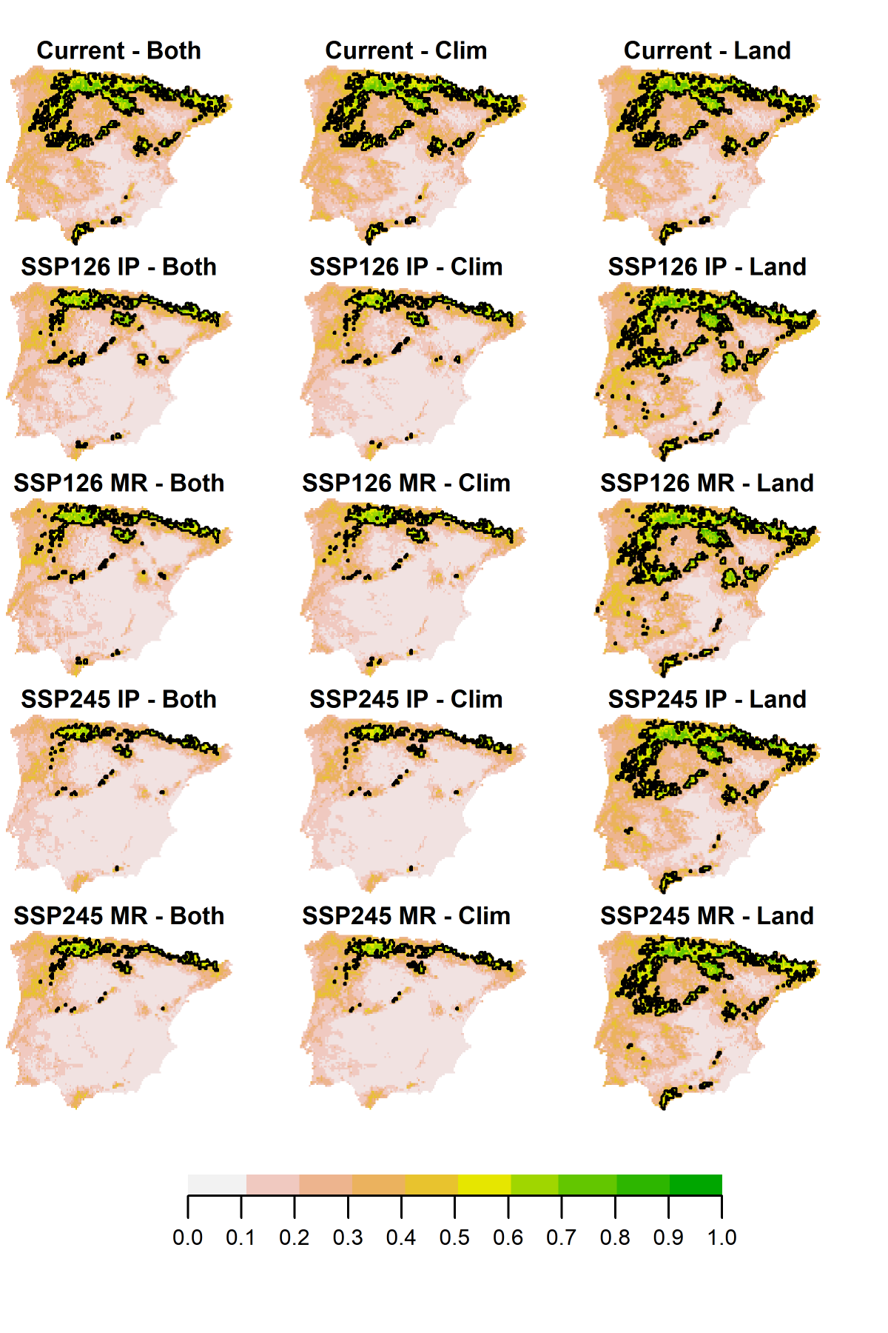
*

**Figure S48.** Map predictions of suitable habitat for *Dryocopus martius.* Continuous predictions (graduated colors) and binary map predictions (polygon) are shown.

*
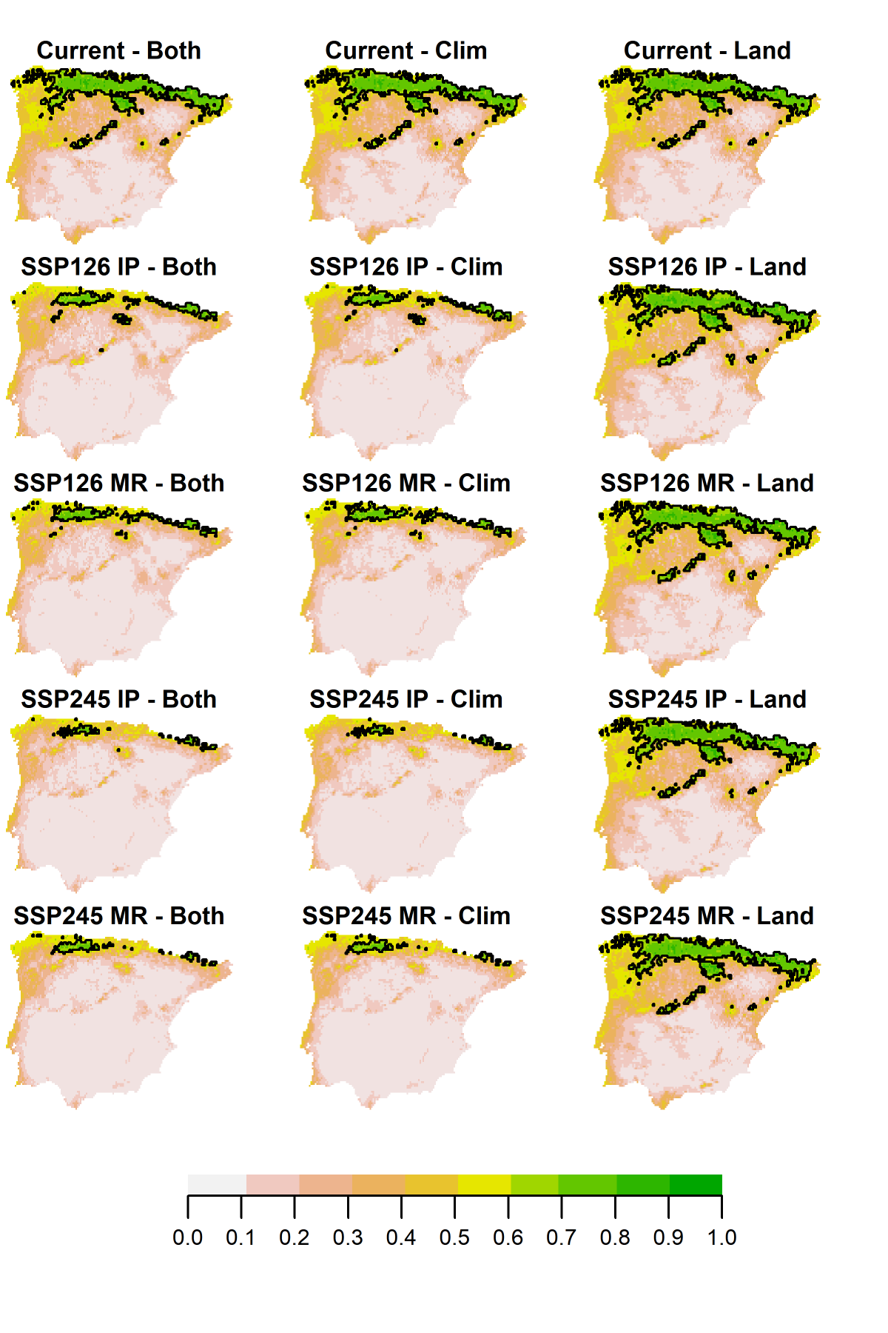
*

**Figure S49.** Map predictions of suitable habitat for *Emberiza citrinella.* Continuous predictions (graduated colors) and binary map predictions (polygon) are shown.

*
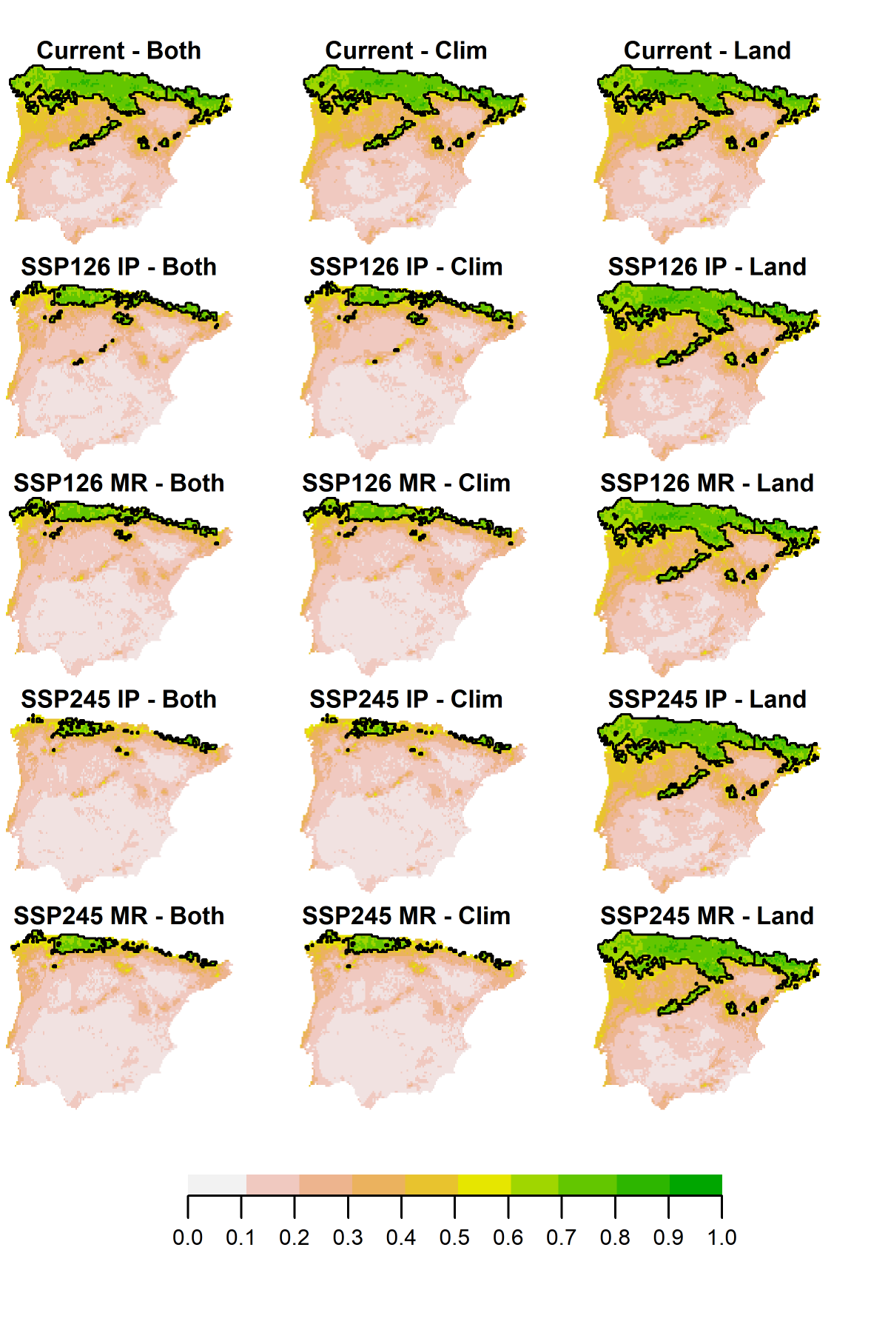
*

**Figure S50.** Map predictions of suitable habitat for *Emberiza hortulana.* Continuous predictions (graduated colors) and binary map predictions (polygon) are shown.

*
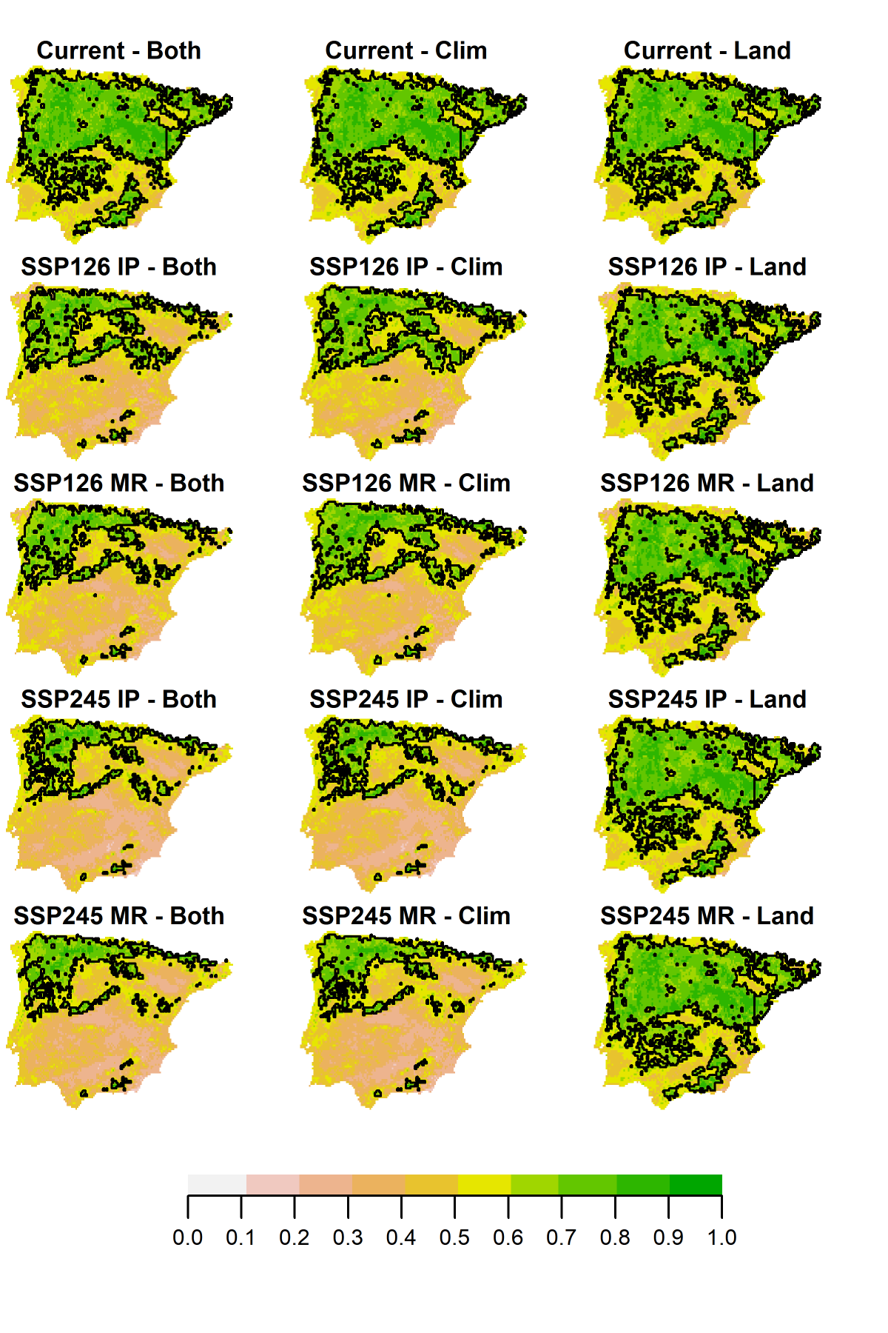
*

**Figure S51.** Map predictions of suitable habitat for *Gallinago gallinago.* Continuous predictions (graduated colors) and binary map predictions (polygon) are shown.


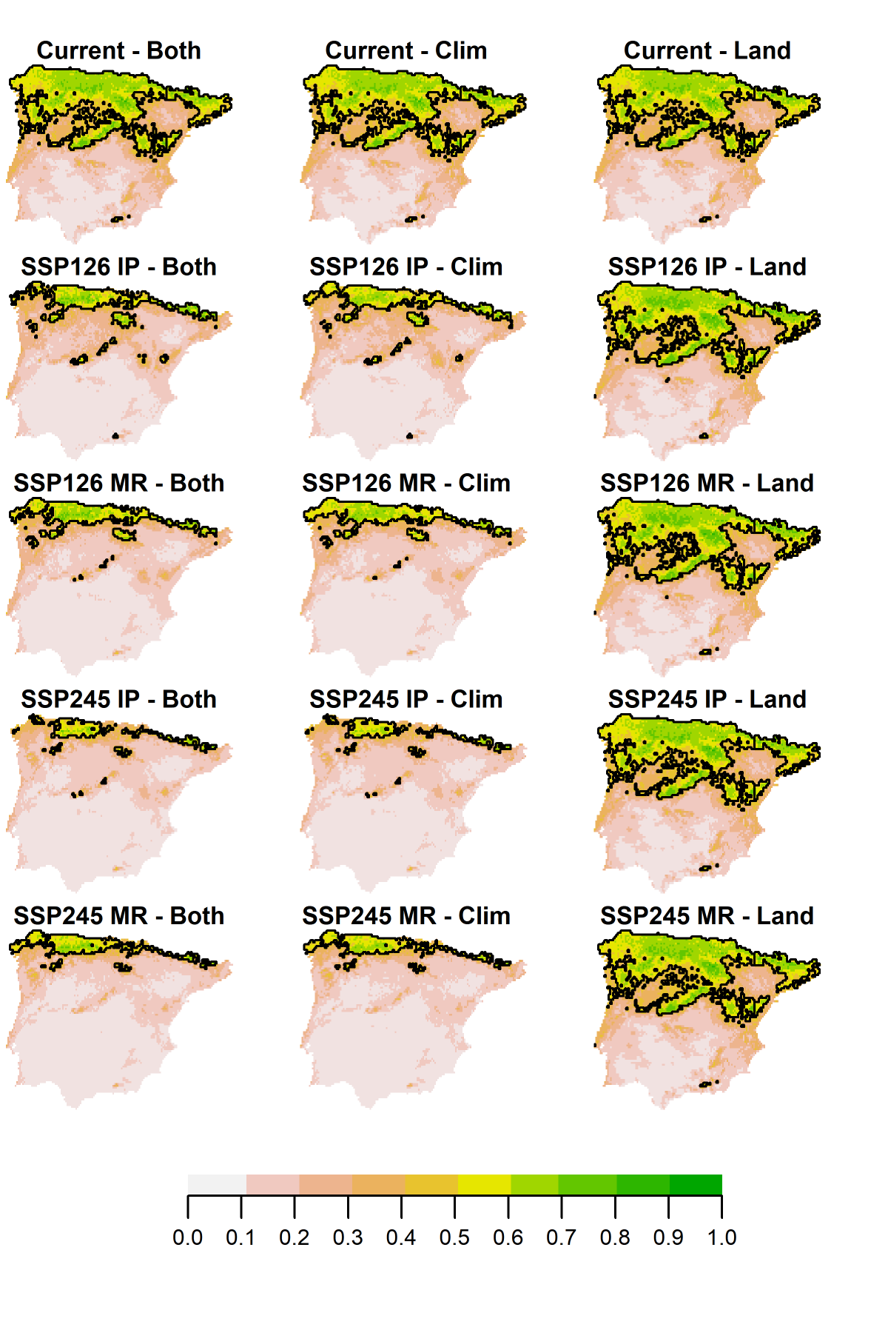


**Figure S52.** Map predictions of suitable habitat for *Lagopus muta.* Continuous predictions (graduated colors) and binary map predictions (polygon) are shown.

*
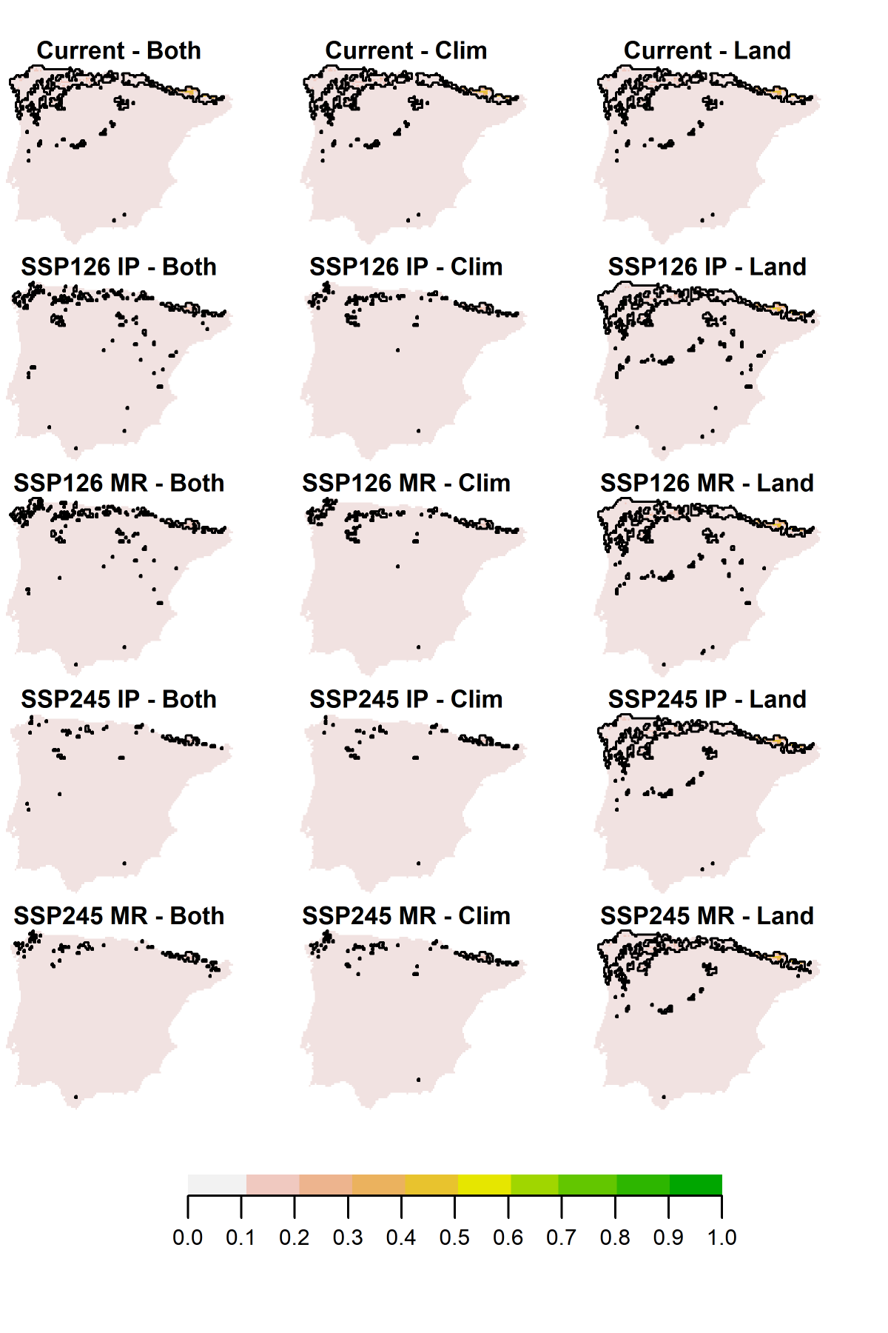
*

**Figure S53.** Map predictions of suitable habitat for *Lanius collurio.* Continuous predictions (graduated colors) and binary map predictions (polygon) are shown.

*
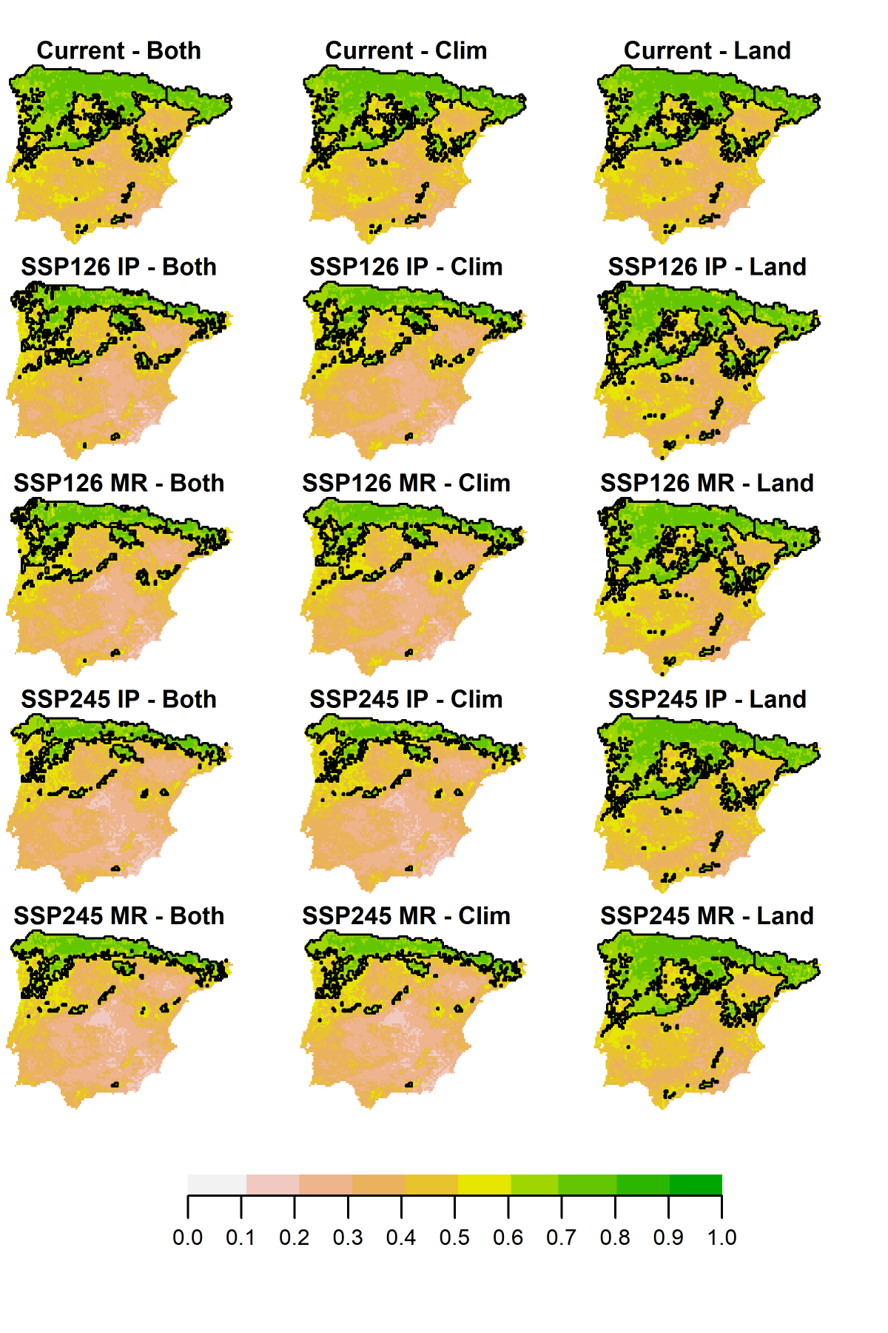
*

**Figure S54.** Map predictions of suitable habitat for *Locustella naevia.* Continuous predictions (graduated colors) and binary map predictions (polygon) are shown.

*
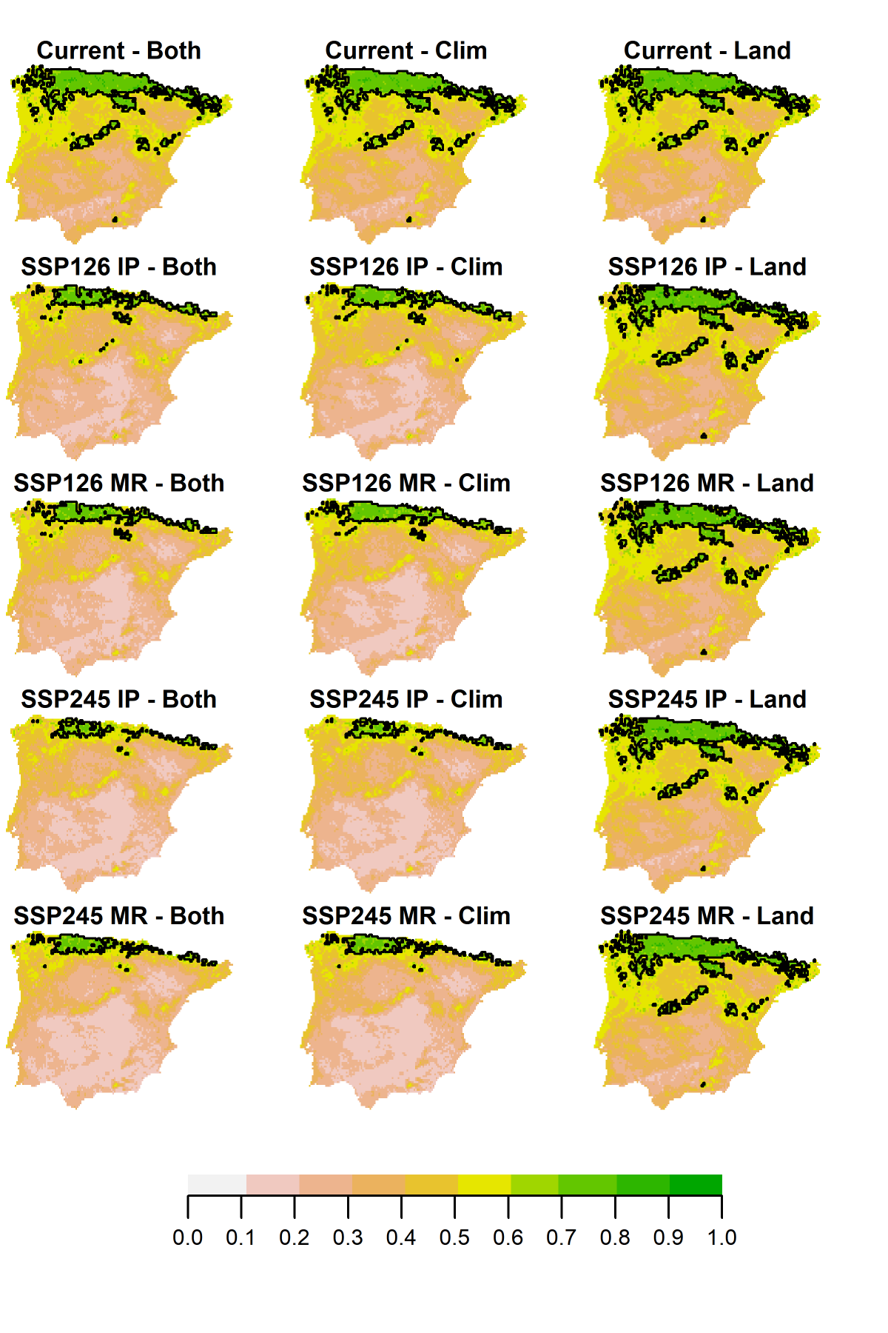
*

**Figure S55.** Map predictions of suitable habitat for *Luscinia svecica.* Continuous predictions (graduated colors) and binary map predictions (polygon) are shown.

*
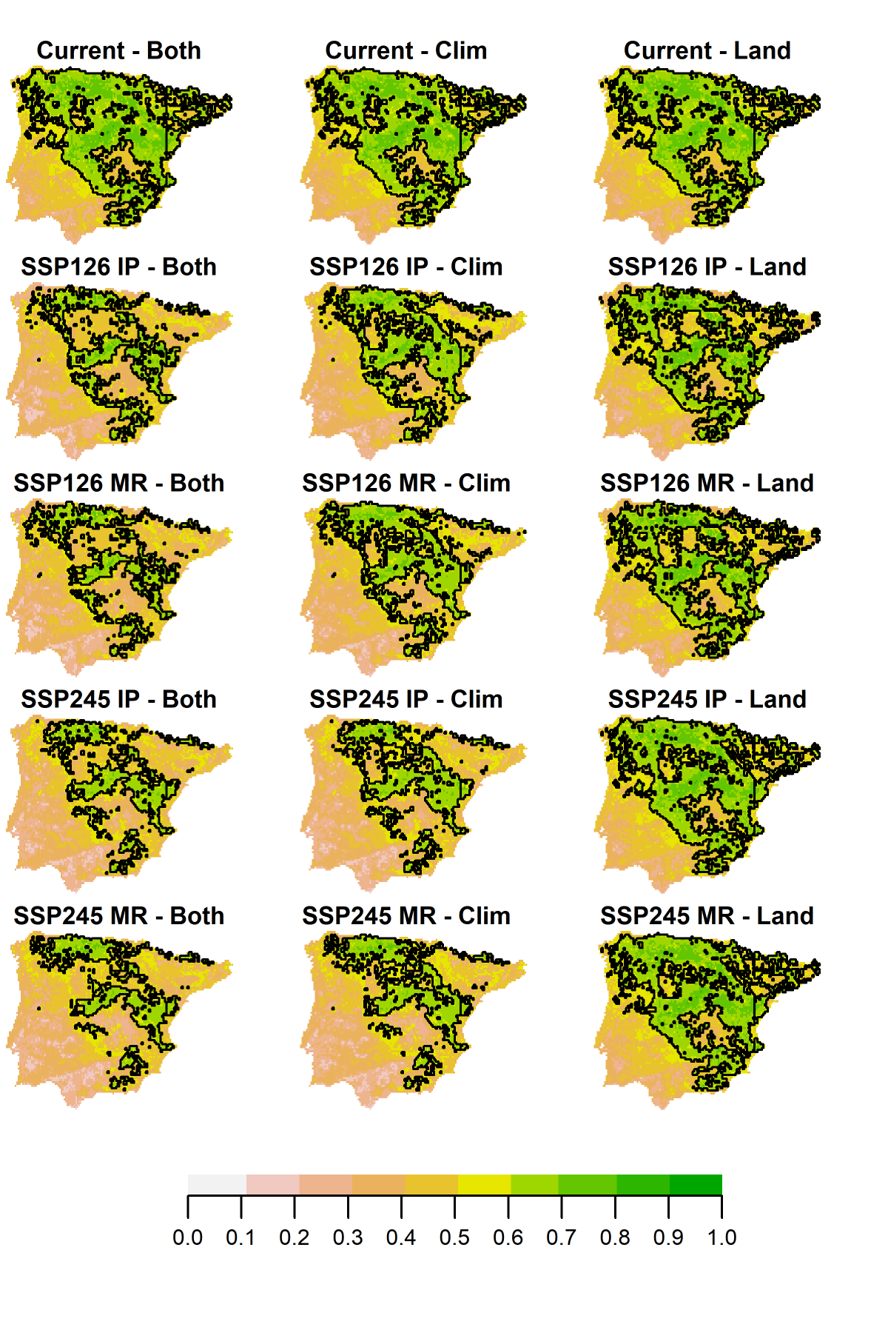
*

**Figure S56.** Map predictions of suitable habitat for *Montifringilla nivalis.* Continuous predictions (graduated colors) and binary map predictions (polygon) are shown.

*
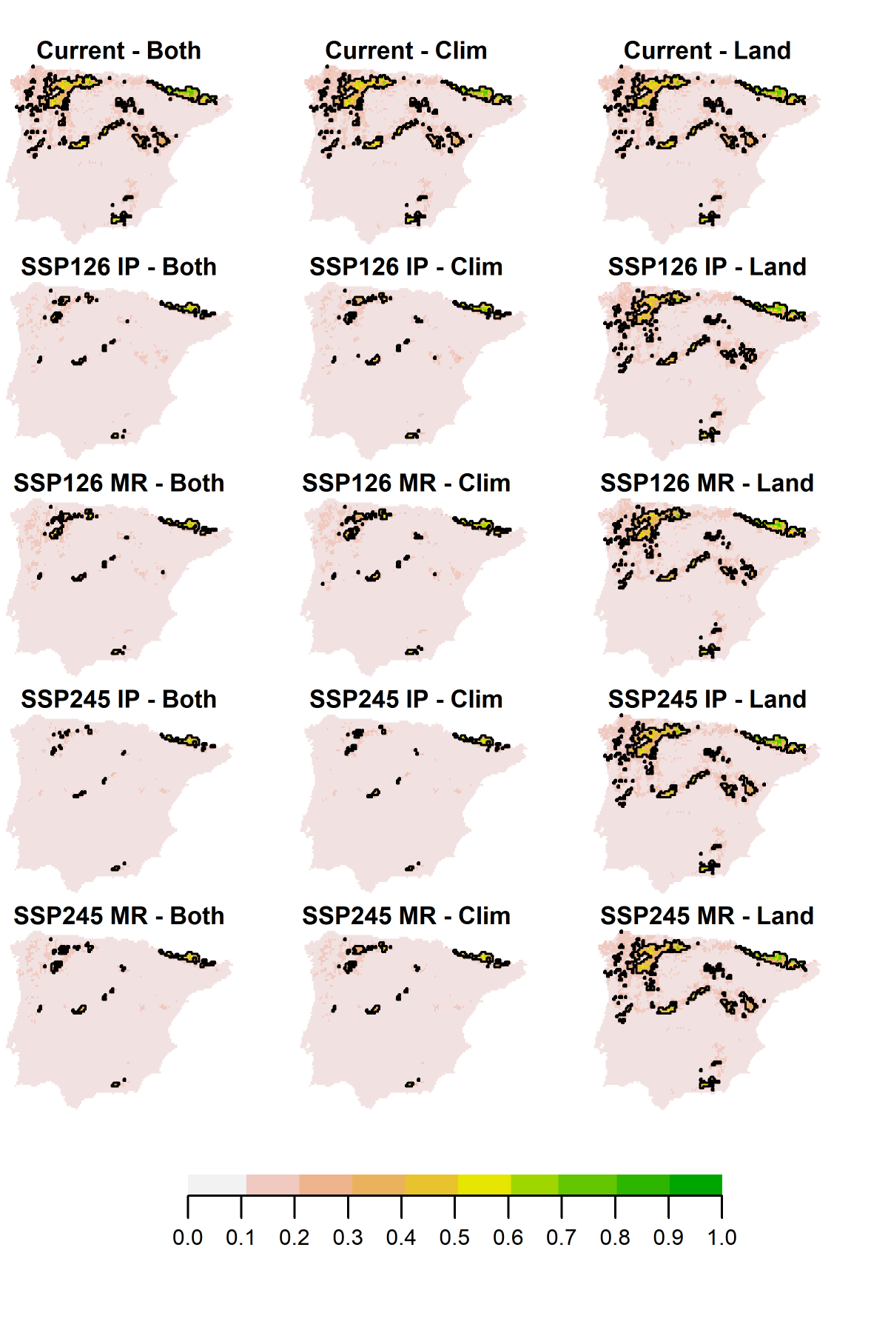
*

**Figure S57.** Map predictions of suitable habitat for *Poecile palustris.* Continuous predictions (graduated colors) and binary map predictions (polygon) are shown.

*
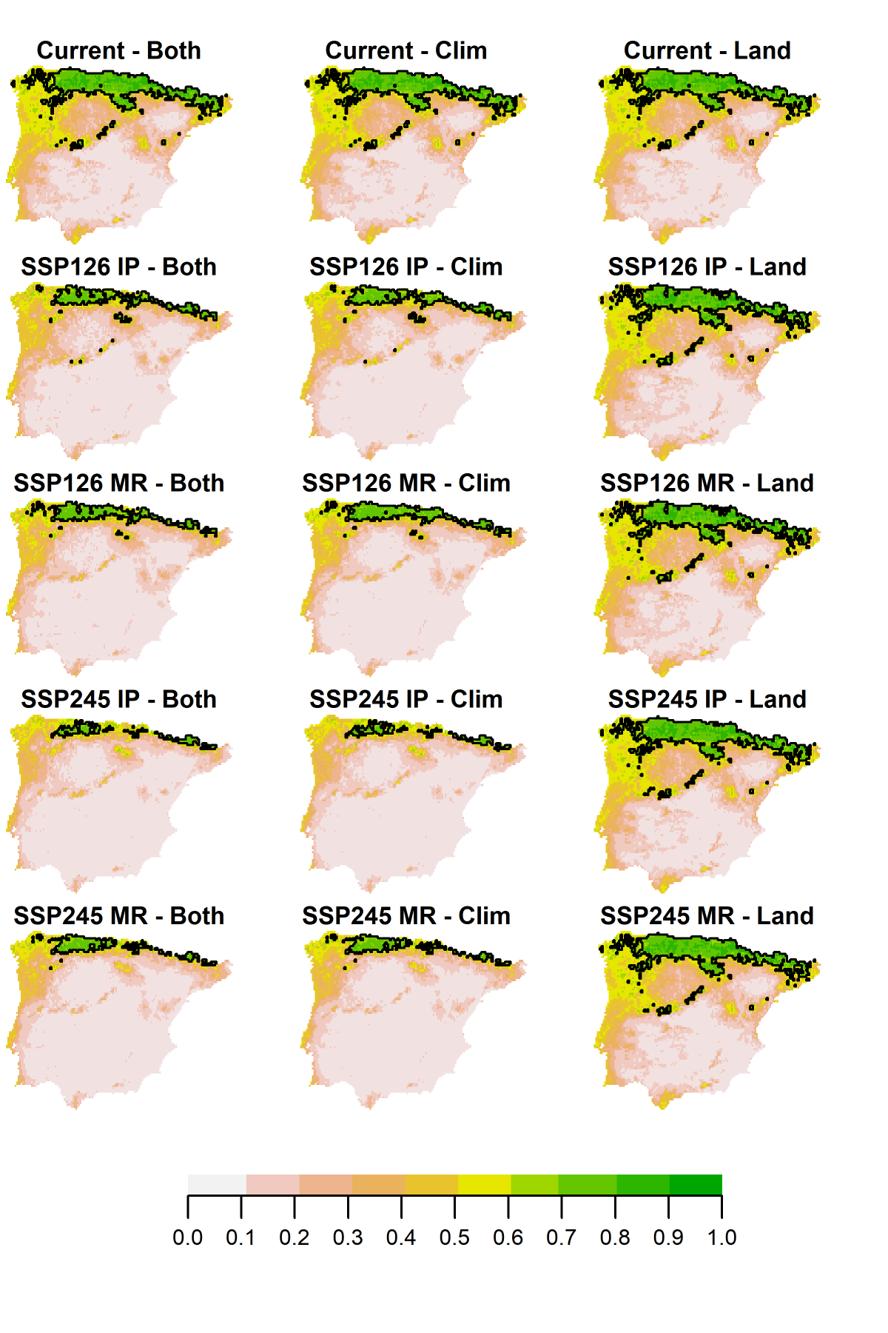
*

**Figure S58.** Map predictions of suitable habitat for *Prunella collaris.* Continuous predictions (graduated colors) and binary map predictions (polygon) are shown.

*
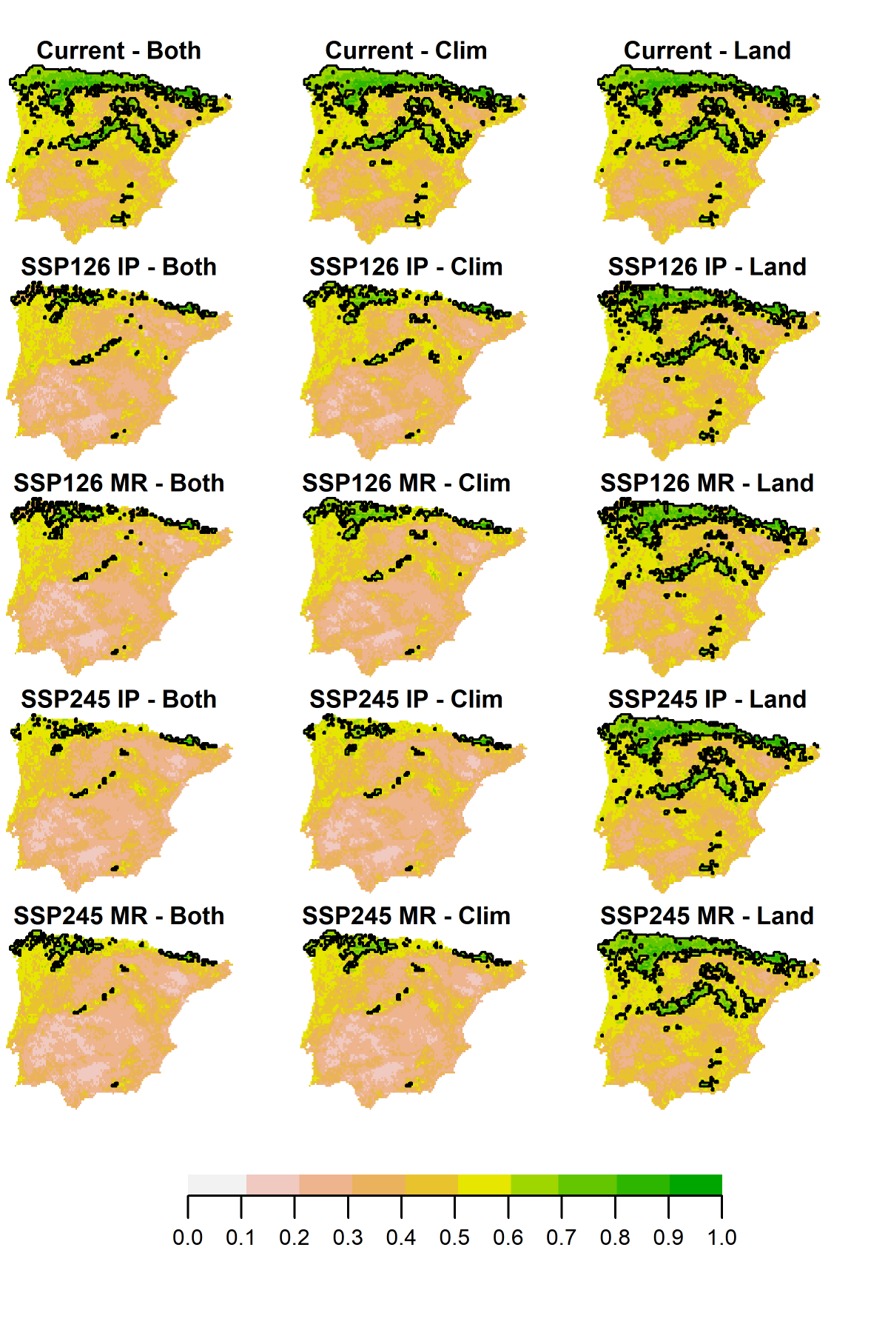
*

**Figure S59.** Map predictions of suitable habitat for *Prunella modularis.* Continuous predictions (graduated colors) and binary map predictions (polygon) are shown.

*
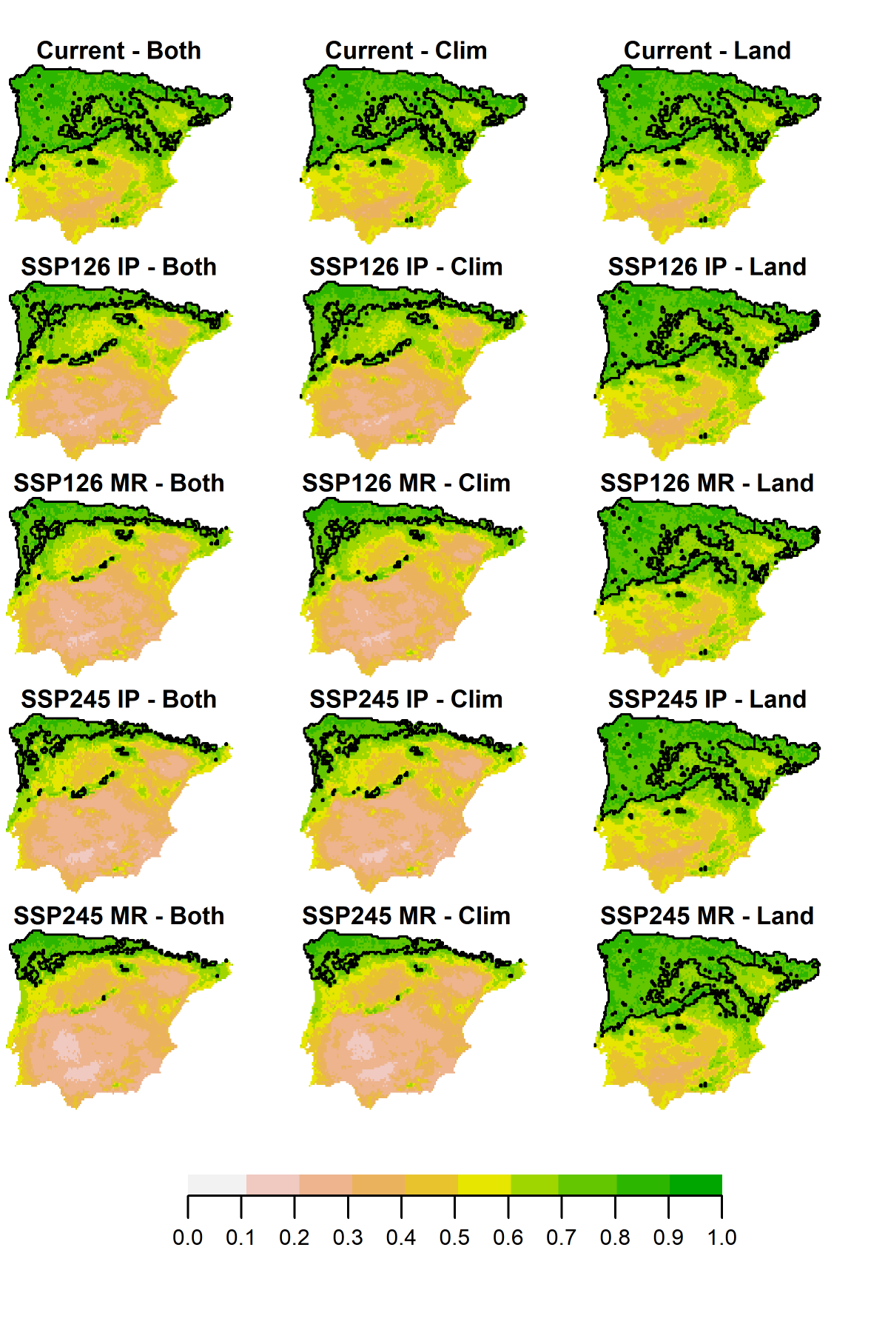
*

**Figure S60.** Map predictions of suitable habitat for *Pyrrhocorax graculus.* Continuous predictions (graduated colors) and binary map predictions (polygon) are shown.

*
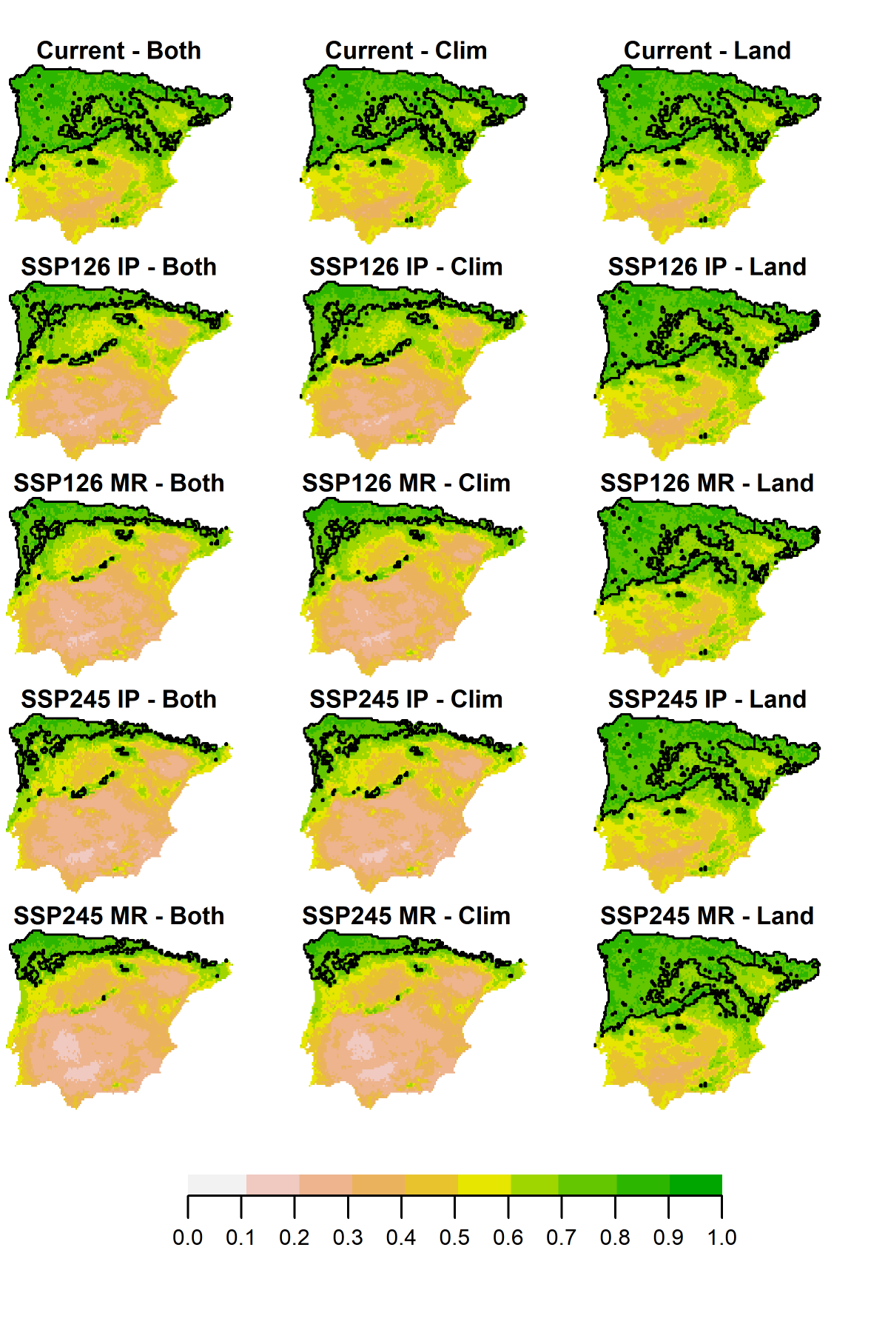
*

**Figure S61.** Map predictions of suitable habitat for *Pyrrhula pyrrhula.* Continuous predictions (graduated colors) and binary map predictions (polygon) are shown.

*
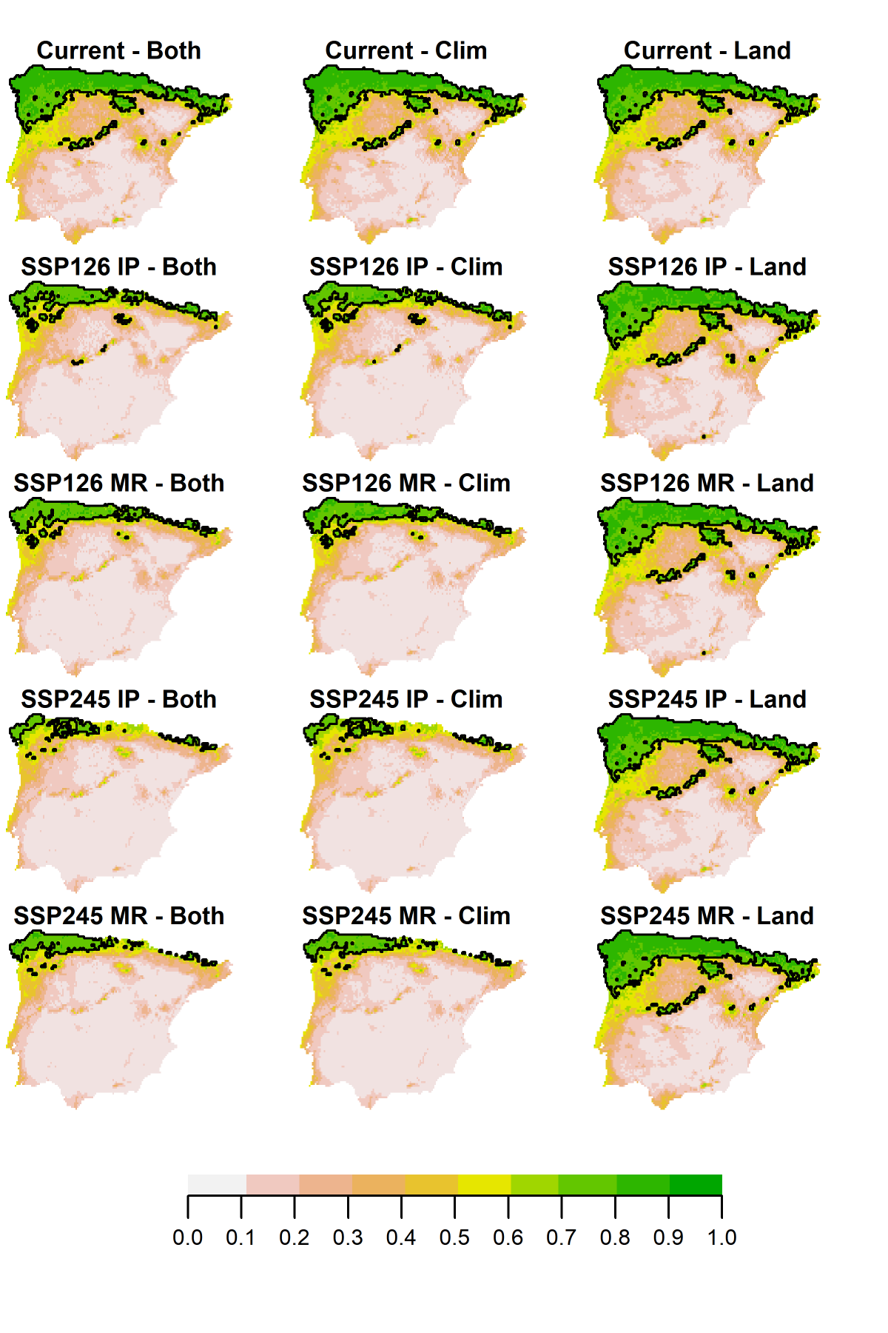
*

**Figure S62.** Map predictions of suitable habitat for *Regulus regulus.* Continuous predictions (graduated colors) and binary map predictions (polygon) are shown.

*
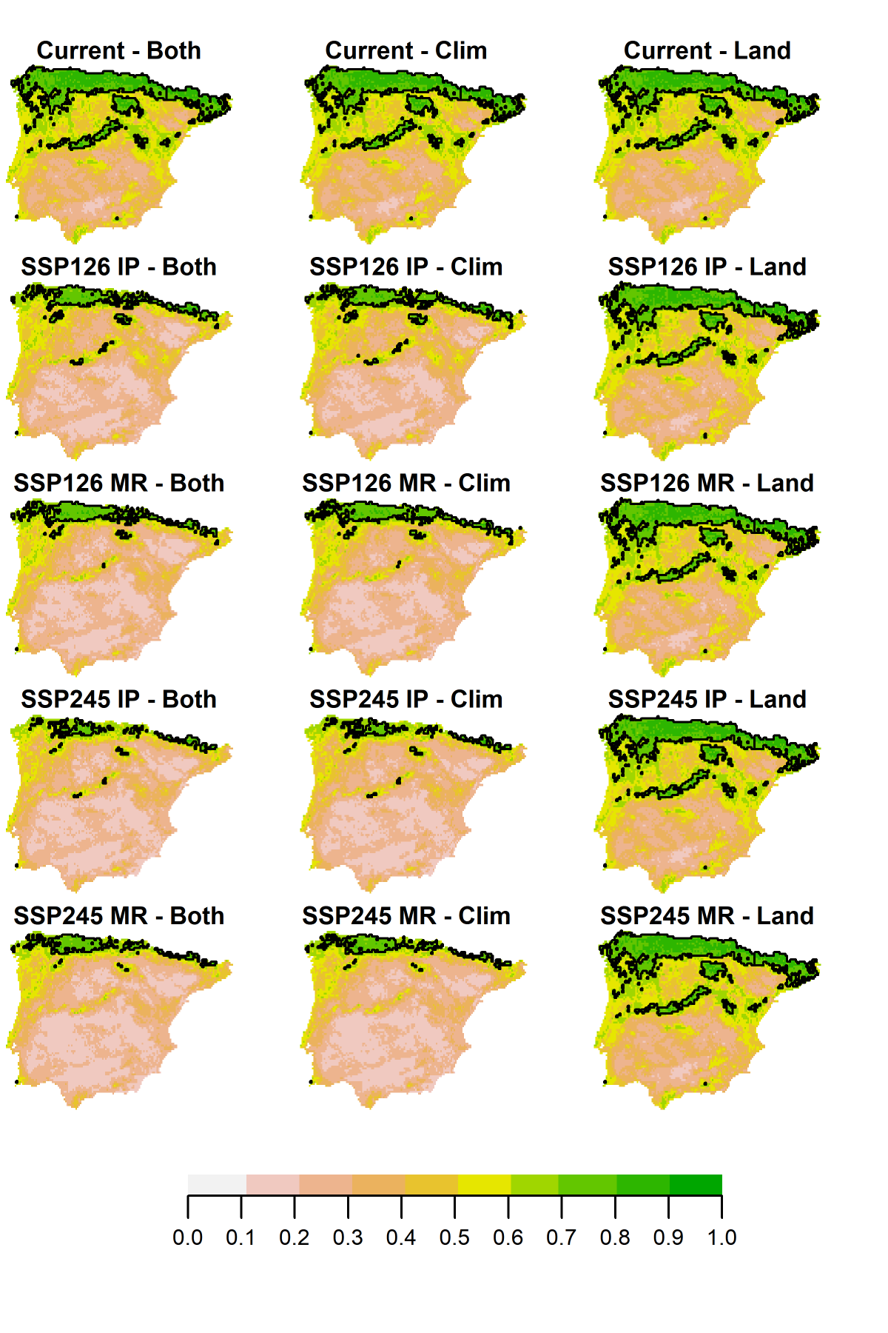
*

**Figure S63.** Map predictions of suitable habitat for *Scolopax rusticola.* Continuous predictions (graduated colors) and binary map predictions (polygon) are shown.

*
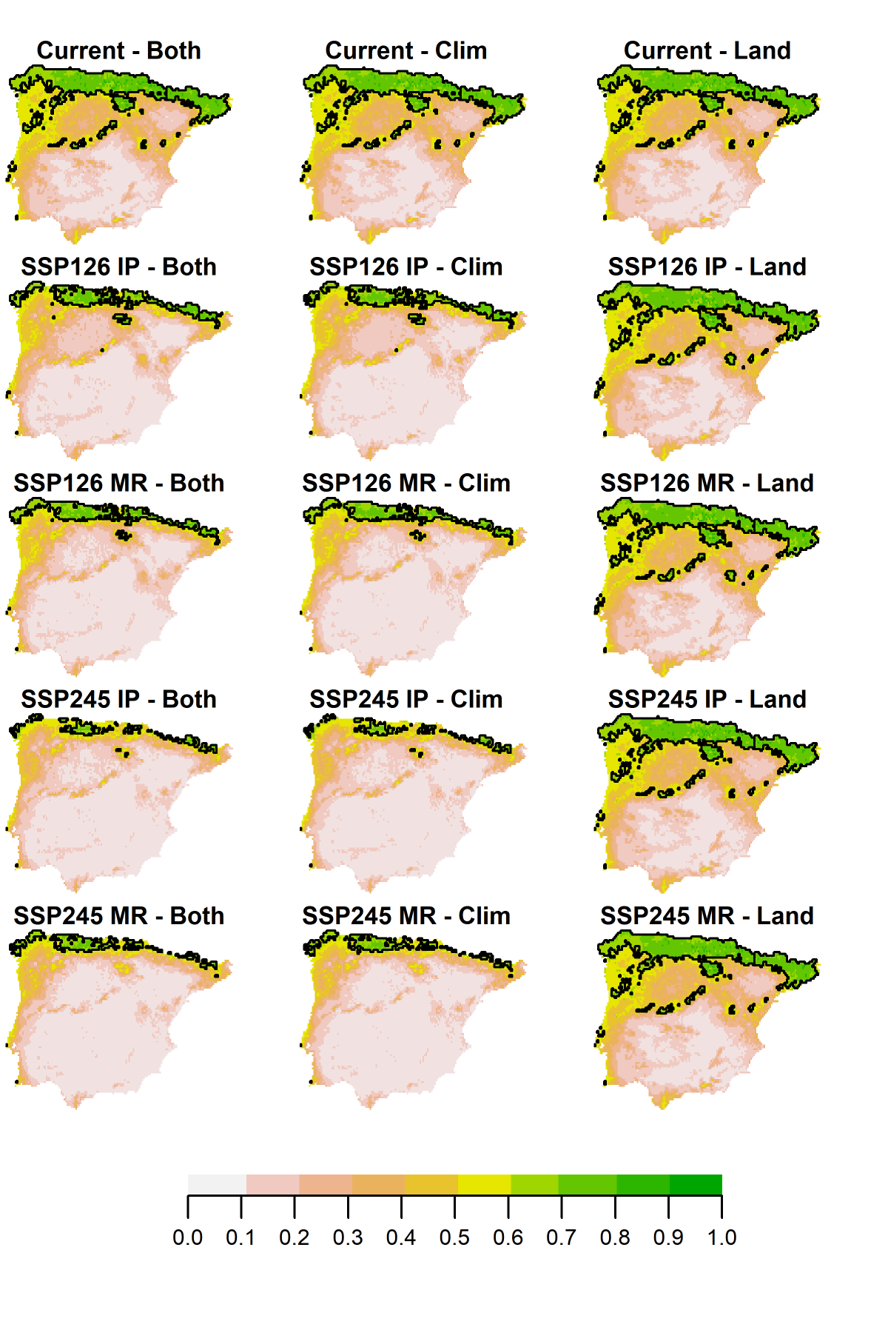
*

**Figure S64.** Map predictions of suitable habitat for *Sylvia borin.* Continuous predictions (graduated colors) and binary map predictions (polygon) are shown.

*
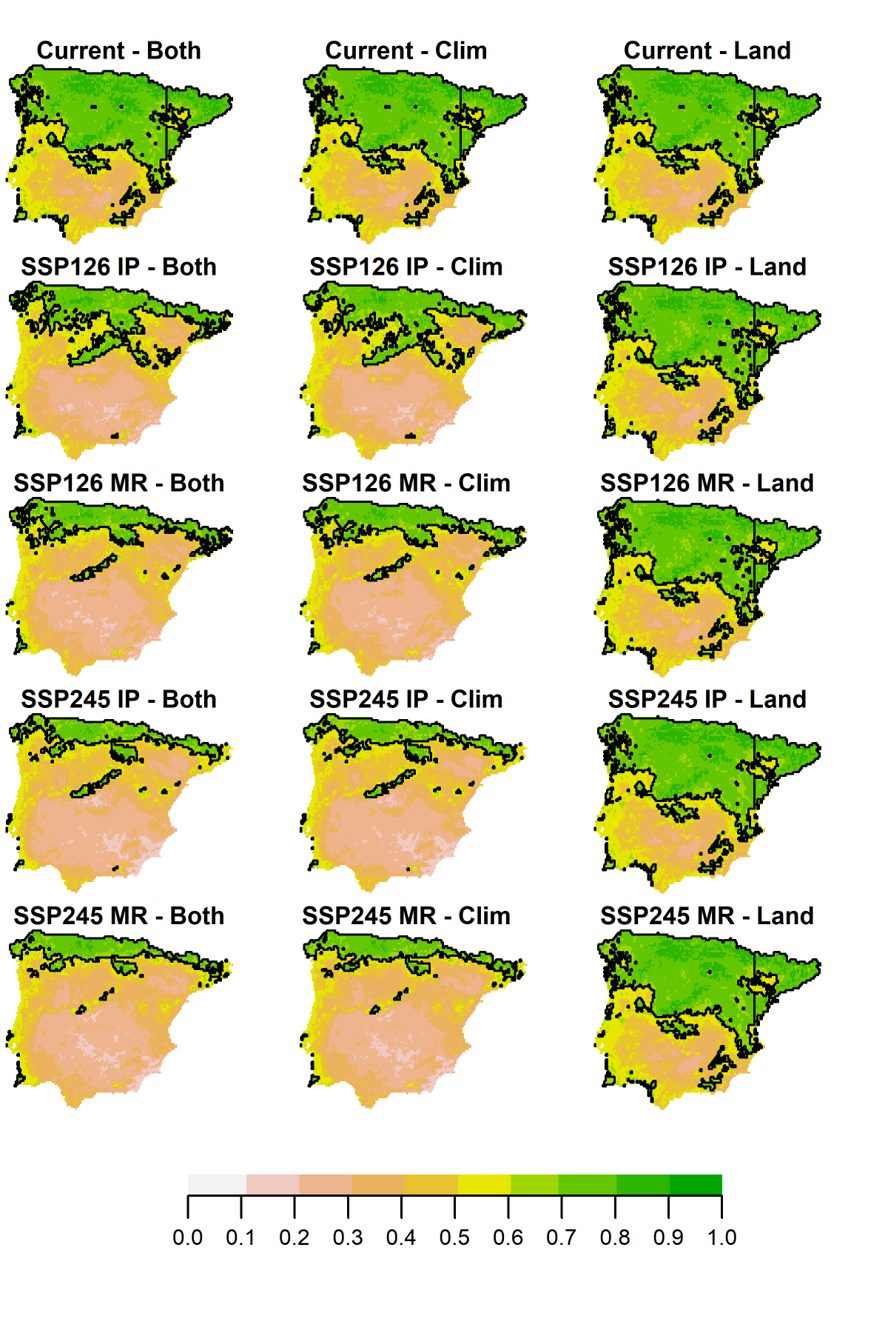
*

**Figure S65.** Map predictions of suitable habitat for *Tetrao urogallus.* Continuous predictions (graduated colors) and binary map predictions (polygon) are shown.

*
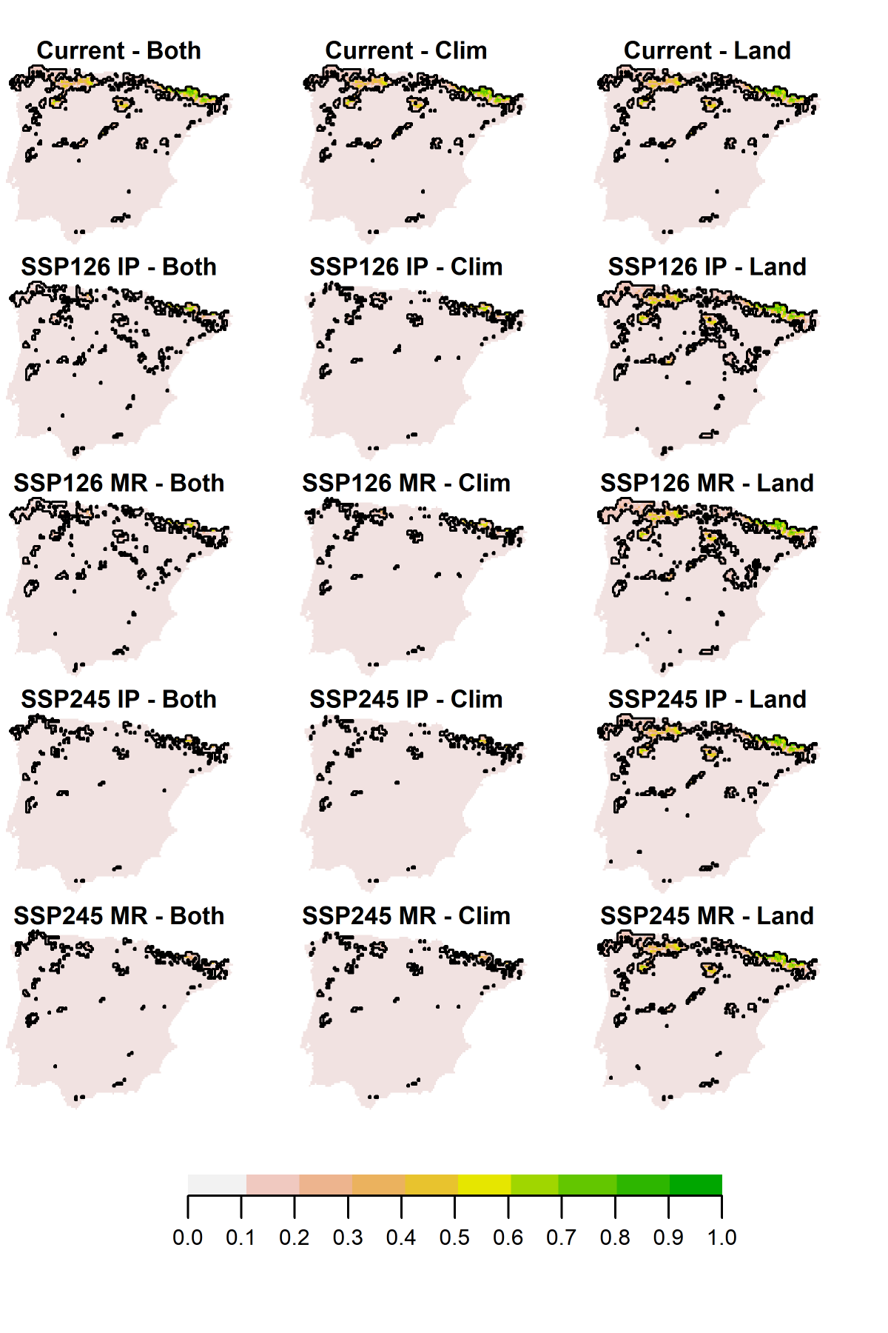
*

**Figure S66.** Map predictions of suitable habitat for *Tichodroma muraria.* Continuous predictions (graduated colors) and binary map predictions (polygon) are shown.

*
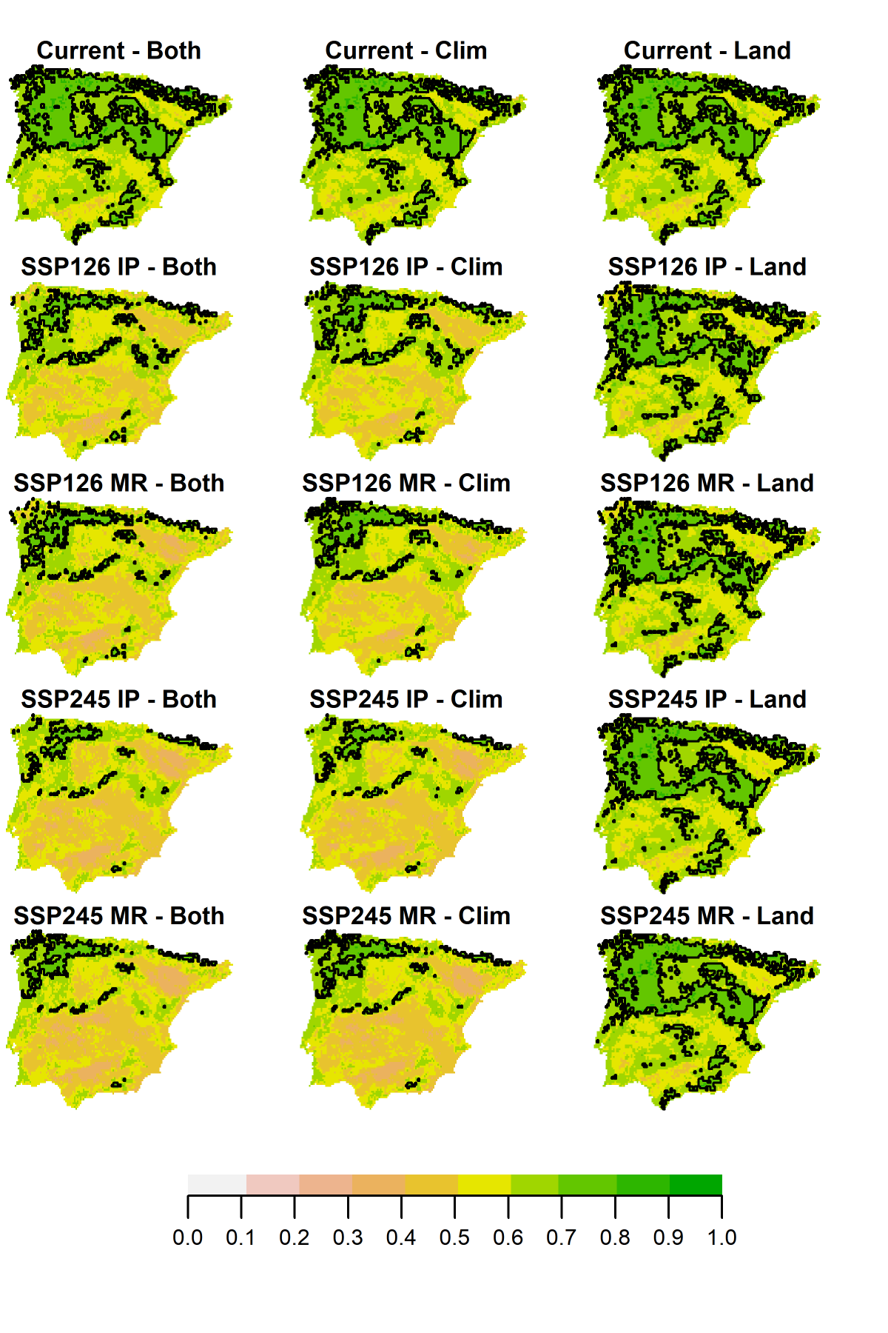
*

**Figure S67.** Map predictions of suitable habitat for *Turdus philomelos.* Continuous predictions (graduated colors) and binary map predictions (polygon) are shown.

*
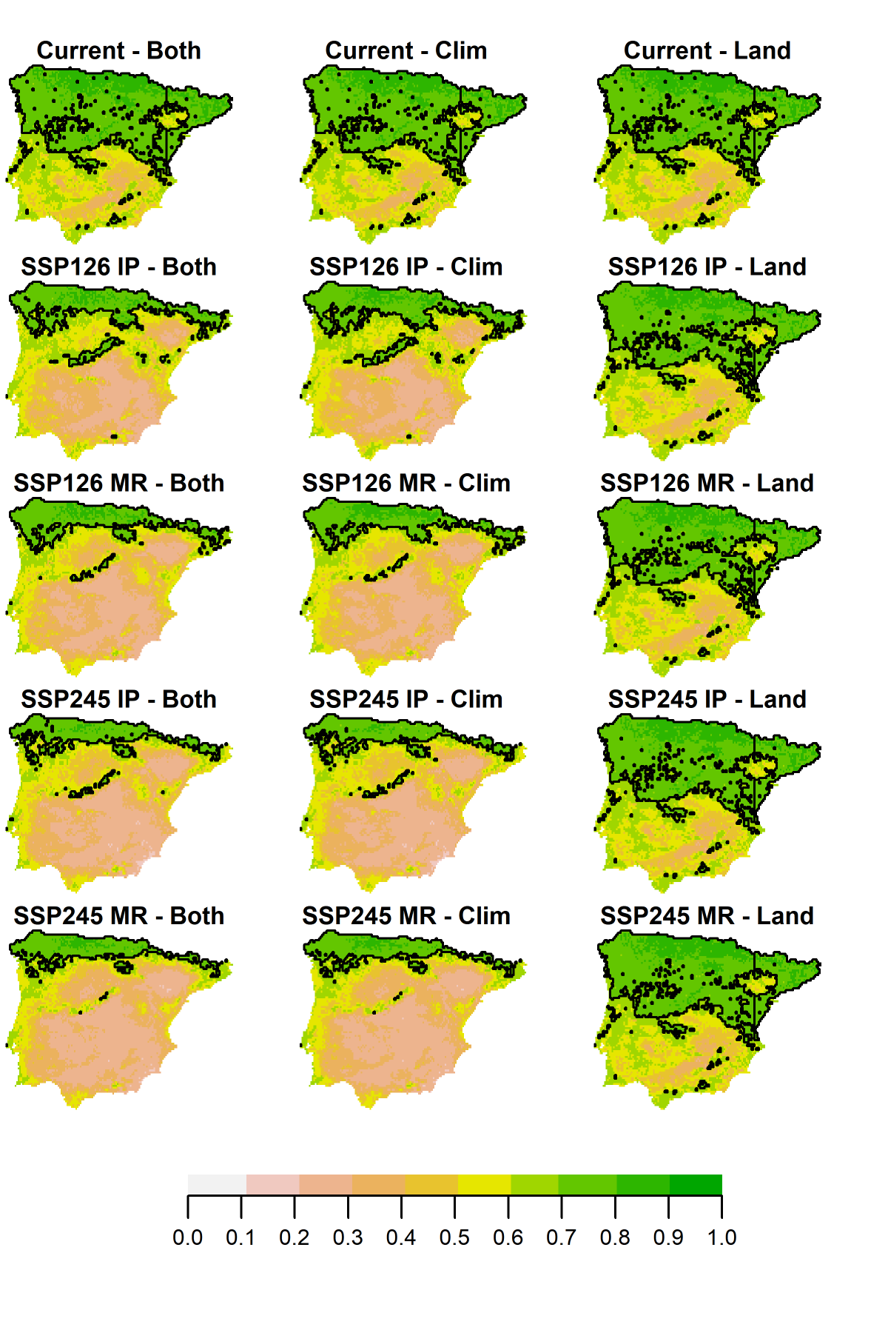
*

**Figure S68.** Map predictions of suitable habitat for *Turdus torquatus.* Continuous predictions (graduated colors) and binary map predictions (polygon) are shown.

*
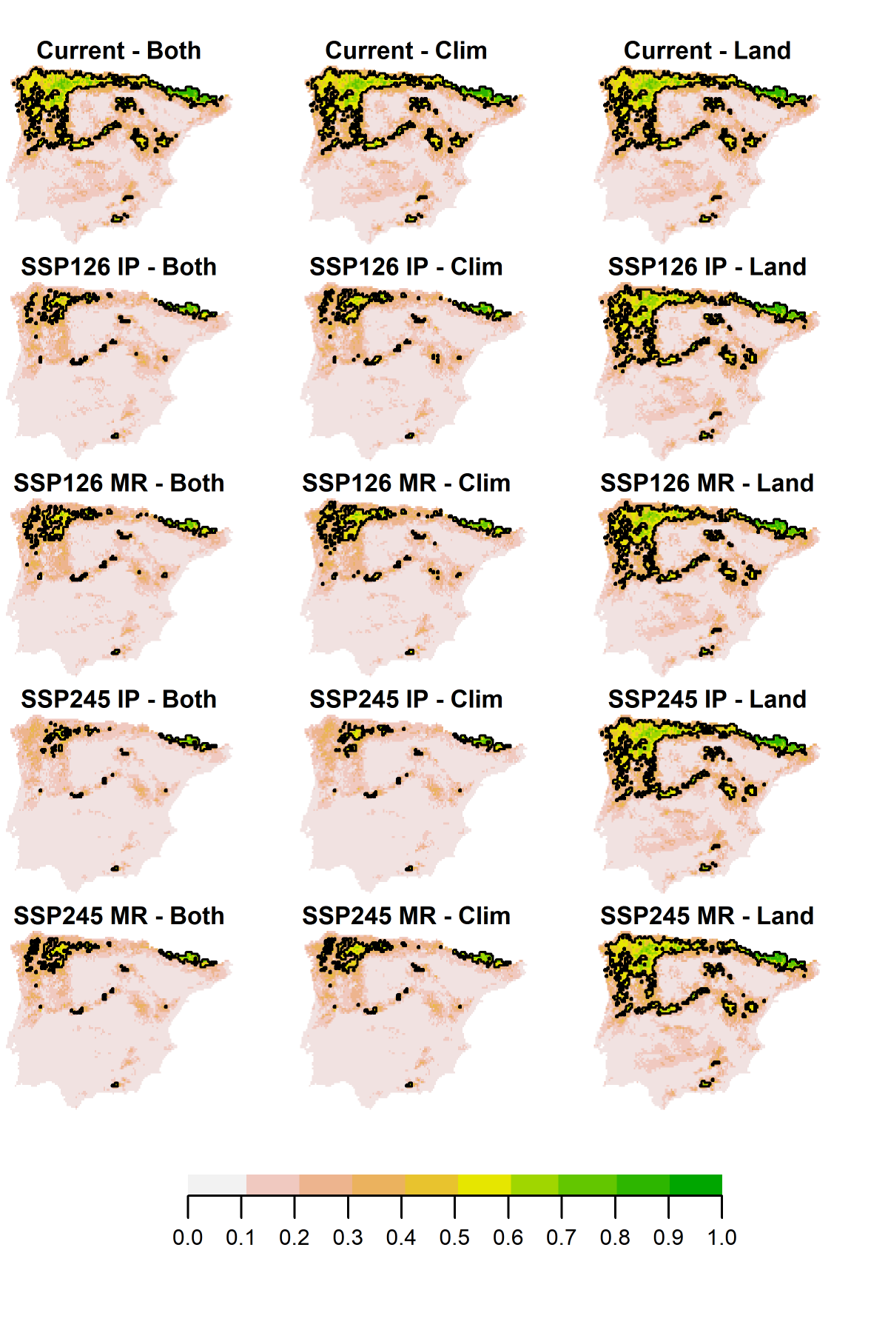
*

**Figure S69.** Map predictions of suitable habitat for *Perdix perdix.* Continuous predictions (graduated colors) and binary map predictions (polygon) are shown.

*
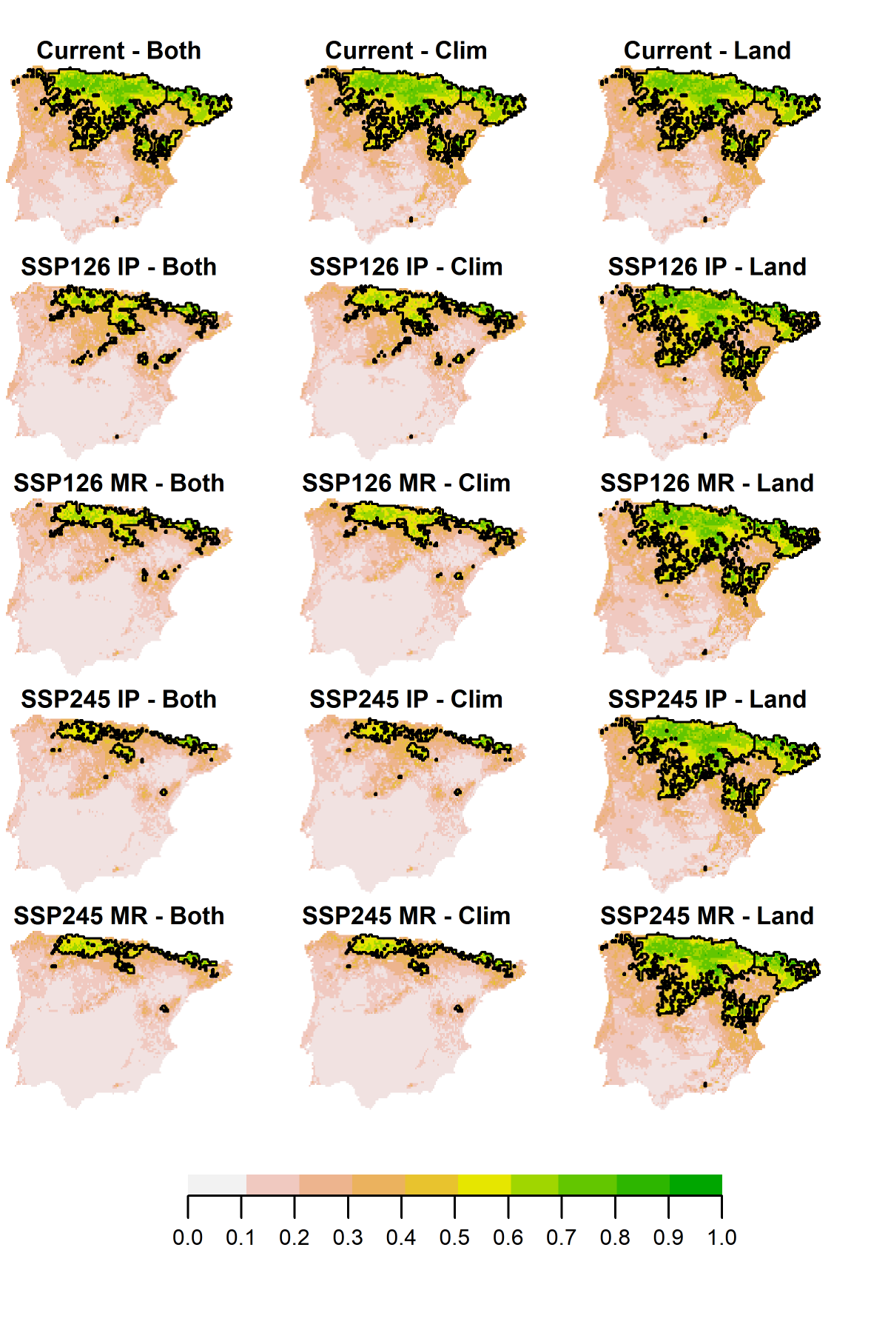
*

**Figure S70.** Map predictions of suitable habitat for *Saxicola rubetra.* Continuous predictions (graduated colors) and binary map predictions (polygon) are shown.

*
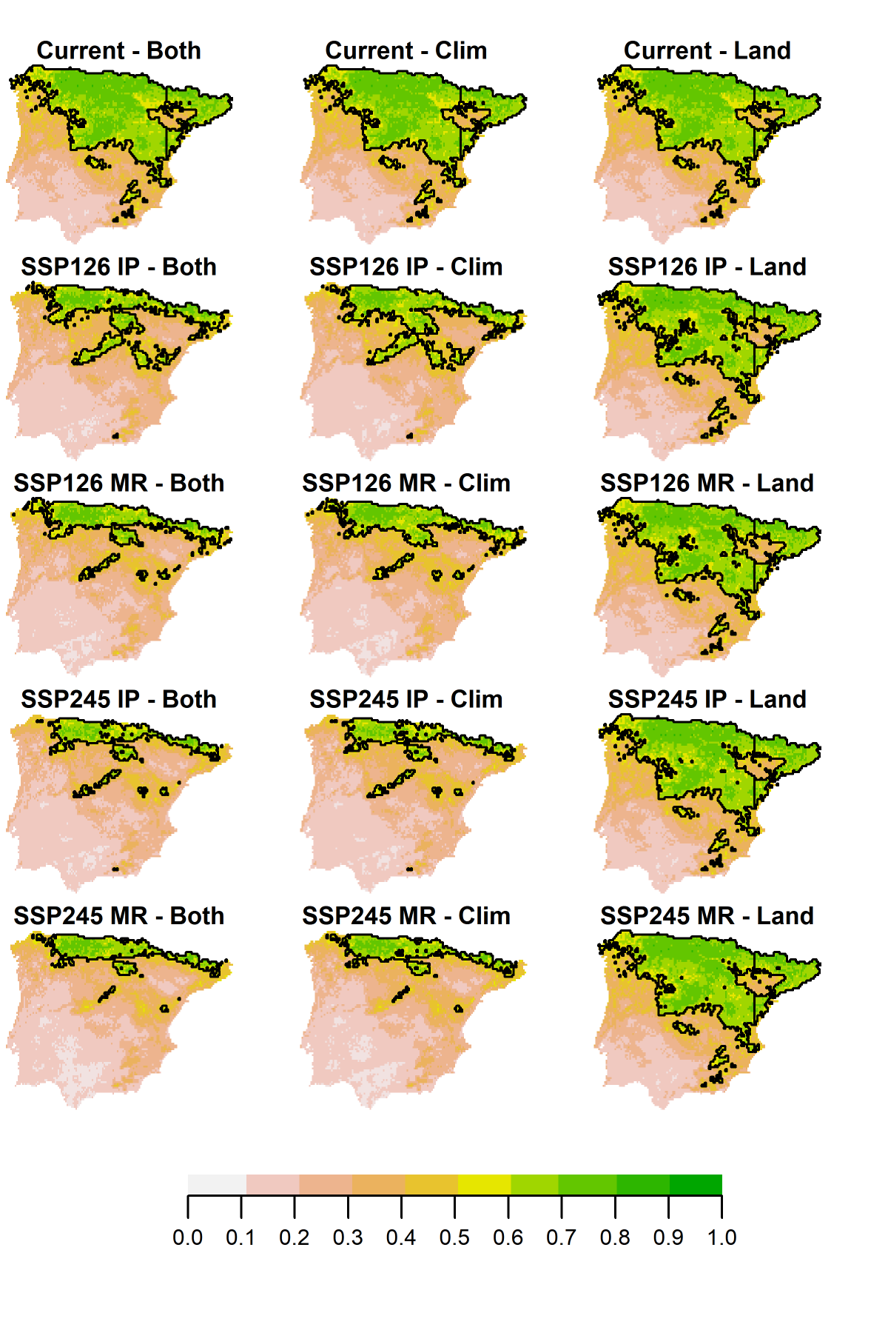
*

| **Table S1.** GBIF occurrence downloads from GBIF.org and access date. | | |
| --- | --- | --- |
| **Species** | **DOI** | **Access date** |
| *Aegolius funereus* | [DOI10.15468/dl.pjusch](https://doi.org/10.15468/dl.pjusch) | 09/12/2020 |
| *Anthus spinoletta* | [DOI10.15468/dl.pwctxe](https://doi.org/10.15468/dl.pwctxe) | 09/12/2020 |
| *Anthus trivialis* | [DOI10.15468/dl.uzjk43](https://doi.org/10.15468/dl.uzjk43) | 09/12/2020 |
| *Carduelis citrinella* | [DOI10.15468/dl.86epjx](https://doi.org/10.15468/dl.86epjx) | 09/12/2020 |
| *Certhia familiaris* | [DOI10.15468/dl.yqz5r6](https://doi.org/10.15468/dl.yqz5r6) | 09/12/2020 |
| *Circus cyaneus* | [DOI10.15468/dl.mpnf4q](https://doi.org/10.15468/dl.mpnf4q) | 09/12/2020 |
| *Corvus frugilegus* | [DOI10.15468/dl.yzg8zk](https://doi.org/10.15468/dl.yzg8zk) | 09/12/2020 |
| *Dendrocopos leucotos* | [DOI10.15468/dl.y3j243](https://doi.org/10.15468/dl.y3j243) | 09/12/2020 |
| *Dendrocoptes medius* | [DOI10.15468/dl.mrj3vt](https://doi.org/10.15468/dl.mrj3vt) | 09/12/2020 |
| *Dryocopus martius* | [DOI10.15468/dl.4s84vs](https://doi.org/10.15468/dl.4s84vs) | 09/12/2020 |
| *Emberiza citrinella* | [DOI10.15468/dl.m9sus4](https://doi.org/10.15468/dl.m9sus4) | 09/12/2020 |
| *Emberiza hortulana* | [DOI10.15468/dl.64szgx](https://doi.org/10.15468/dl.64szgx) | 09/12/2020 |
| *Gallinago gallinago* | [DOI10.15468/dl.j9akcq](https://doi.org/10.15468/dl.j9akcq) | 09/12/2020 |
| *Lagopus muta* | [DOI10.15468/dl.dbfnke](https://doi.org/10.15468/dl.dbfnke) | 09/12/2020 |
| *Lanius collurio* | [DOI10.15468/dl.tv2ja4](https://doi.org/10.15468/dl.tv2ja4) | 09/12/2020 |
| *Locustella naevia* | [DOI10.15468/dl.ye6859](https://doi.org/10.15468/dl.ye6859) | 09/12/2020 |
| *Luscinia svecica* | [DOI10.15468/dl.ts5jcz](https://doi.org/10.15468/dl.ts5jcz) | 09/12/2020 |
| *Montifringilla nivalis* | [DOI10.15468/dl.3tmzr4](https://doi.org/10.15468/dl.3tmzr4) | 09/12/2020 |
| *Perdix perdix* | [DOI10.15468/dl.j3d6qq](https://doi.org/10.15468/dl.j3d6qq) | 09/12/2020 |
| *Poecile palustris* | [DOI10.15468/dl.kdbv2m](https://doi.org/10.15468/dl.kdbv2m) | 09/12/2020 |
| *Prunella collaris* | [DOI10.15468/dl.zamxnm](https://doi.org/10.15468/dl.zamxnm) | 09/12/2020 |
| *Prunella modularis* | [DOI10.15468/dl.95dx76](https://doi.org/10.15468/dl.95dx76) | 09/12/2020 |
| *Pyrrhocorax graculus* | [DOI10.15468/dl.2mktkk](https://doi.org/10.15468/dl.2mktkk) | 09/12/2020 |
| *Pyrrhula pyrrhula* | [DOI10.15468/dl.k4w9vr](https://doi.org/10.15468/dl.k4w9vr) | 09/12/2020 |
| *Regulus regulus* | [DOI10.15468/dl.c9cw89](https://doi.org/10.15468/dl.c9cw89) | 09/12/2020 |
| *Saxicola rubetra* | [DOI10.15468/dl.pmjd7s](https://doi.org/10.15468/dl.pmjd7s) | 09/12/2020 |
| *Scolopax rusticola* | [DOI10.15468/dl.jcxuwq](https://doi.org/10.15468/dl.jcxuwq) | 09/12/2020 |
| *Sylvia borin* | [DOI10.15468/dl.eznbey](https://doi.org/10.15468/dl.eznbey) | 09/12/2020 |
| *Tetrao urogallus* | [DOI10.15468/dl.fs5uck](https://doi.org/10.15468/dl.fs5uck) | 09/12/2020 |
| *Tichodroma muraria* | [DOI10.15468/dl.gnbju4](https://doi.org/10.15468/dl.gnbju4) | 09/12/2020 |
| *Turdus philomelos* | [DOI10.15468/dl.s995pj](https://doi.org/10.15468/dl.s995pj) | 09/12/2020 |
| *Turdus torquatus* | [DOI10.15468/dl.9x27ee](https://doi.org/10.15468/dl.9x27ee) | 09/12/2020 |

| **Table S2.** Climate and land cover characteristics of socio-economic scenarios (SSP1-2.6, SSP2-4.5) based on MIROC general circulation model and current conditions. Mean ± SD and range (in parenthesis) of, minimum temperature of the coldest month (Tmin), maximum temperature of the warmest month (Tmax), annual precipitation (Pan), precipitation seasonality (coefficient of variation of monthly values, Pcv) and percentage of different land cover types at 5-arcminute resolution are shown. | | | |
| --- | --- | --- | --- |
| **Variables** | **Current** | **SSP1-2.6 (MIROC)** | **SSP2-4.5 (MIROC)** |
| **Climate** |  |  |  |
| Tmin (ºC) | 1.6 ± 2.9 (-10.0 - 9.3) | 2.6 ± 2.8 (-8.9 - 10.1) | 3.1 ± 2.7 (-8.4 - 10.7) |
| Tmax (ºC) | 29.5 ± 3.6 (17.7 - 36.5) | 33.5 ± 4.1 (22.3 - 40.6) | 34.6 ± 4.2 (22.9 - 41.7) |
| Pan (mm) | 674 ± 321 (224 - 1925) | 638 ± 297 (215 - 1806) | 629 ± 295 (211 - 1798) |
| Pcv | 0.43 ± 0.14 (0.15 - 0.79) | 0.45 ± 0.14 (0.16 - 0.80) | 0.46 ± 0.15 (0.16 - 0.82) |
| **Land cover types** | | | |
| Needleleaf (%) | 7.9 ± 17.7 (0 - 100) | 13.7 ± 25.0 (0 - 100) | 8.4 ± 18.9 (0 - 100) |
| Broadleaf (%) | 2.5 ± 6.1 (0 - 75) | 4.3 ± 10.5 (0 - 100) | 2.6 ± 6.5 (0 - 76) |
| Grasslands (%) | 40.9 ± 33.3 (0 - 100) | 32.7 ± 33.7 (0 - 100) | 37.6 ± 32.7 (0 - 100) |
| Shrubs (%) | 21.2 ± 28.2 (0 - 100) | 16.1 ± 26.8 (0 - 100) | 19.3 ± 26.8 (0 - 100) |
| Crops (%) | 25.5 ± 27.2 (0 - 100) | 31.2 ± 31.6 (0 - 100) | 30.1 ± 28.6 (0 - 100) |
| Urban (%) | 1.2 ± 3.4 (0 - 78) | 1.2 ± 3.4 (0 - 78) | 1.2 ± 3.4 (0 - 78) |

| **Table S3.** Model accuracy metrics for CLIMLAND models validated at a global scale and Iberia. | | | | | | |  |  |
| --- | --- | --- | --- | --- | --- | --- | --- | --- |
| Species | Global | | | Iberia | | | | |
|  | AUC | Boyce | TSS | AUC | Boyce | TSS | |  |
| *Aegolius funereus* | 1.00 | 0.94 | 0.91 | 0.99 | 0.98 | 0.92 | |  |
| *Anthus spinoletta* | 0.99 | 1.00 | 0.89 | 0.94 | 0.92 | 0.66 | |  |
| *Anthus trivialis* | 0.98 | 0.98 | 0.87 | 0.95 | 0.99 | 0.79 | |  |
| *Carduelis citrinella* | 0.99 | 0.99 | 0.91 | 0.93 | 0.95 | 0.71 | |  |
| *Certhia familiaris* | 1.00 | 0.99 | 0.94 | 0.98 | 0.99 | 0.89 | |  |
| *Circus cyaneus* | 0.99 | 0.99 | 0.91 | 0.81 | 0.91 | 0.45 | |  |
| *Corvus frugilegus* | 0.98 | 0.99 | 0.85 | 0.84 | 0.61 | 0.69 | |  |
| *Dendrocopos leucotos* | 1.00 | 1.00 | 0.92 | 0.99 | 0.96 | 0.86 | |  |
| *Dendrocoptes medius* | 0.99 | 0.98 | 0.88 | 0.96 | 1.00 | 0.82 | |  |
| *Dryocopus martius* | 0.99 | 0.98 | 0.92 | 0.97 | 1.00 | 0.86 | |  |
| *Emberiza citrinella* | 0.99 | 0.98 | 0.88 | 0.97 | 1.00 | 0.84 | |  |
| *Emberiza hortulana* | 0.97 | 1.00 | 0.83 | 0.85 | 0.92 | 0.48 | |  |
| *Gallinago gallinago* | 0.98 | 0.95 | 0.85 | 0.87 | 0.87 | 0.62 | |  |
| *Lagopus muta* | 1.00 | 1.00 | 0.93 | 0.99 | 0.99 | 0.85 | |  |
| *Lanius collurio* | 0.99 | 0.98 | 0.92 | 0.94 | 1.00 | 0.72 | |  |
| *Locustella naevia* | 0.99 | 0.95 | 0.92 | 0.95 | 1.00 | 0.81 | |  |
| *Luscinia svecica* | 0.99 | 0.98 | 0.86 | 0.87 | 0.99 | 0.46 | |  |
| *Montifringilla nivalis* | 1.00 | 0.91 | 0.93 | 0.98 | 0.96 | 0.87 | |  |
| *Poecile palustris* | 0.97 | 0.97 | 0.85 | 0.98 | 0.99 | 0.87 | |  |
| *Prunella collaris* | 0.98 | 0.93 | 0.86 | 0.97 | 0.97 | 0.78 | |  |
| *Prunella modularis* | 0.99 | 1.00 | 0.91 | 0.95 | 0.96 | 0.77 | |  |
| *Pyrrhocorax graculus* | 0.98 | 0.99 | 0.86 | 0.94 | 1.00 | 0.74 | |  |
| *Pyrrhula pyrrhula* | 0.99 | 0.93 | 0.91 | 0.98 | 1.00 | 0.88 | |  |
| *Regulus regulus* | 0.99 | 0.96 | 0.86 | 0.95 | 0.95 | 0.80 | |  |
| *Scolopax rusticola* | 0.98 | 0.98 | 0.89 | 0.94 | 0.89 | 0.78 | |  |
| *Sylvia borin* | 0.98 | 0.99 | 0.78 | 0.90 | 0.98 | 0.58 | |  |
| *Tetrao urogallus* | 0.99 | 0.79 | 0.91 | 0.98 | 0.89 | 0.85 | |  |
| *Tichodroma muraria* | 0.96 | 0.96 | 0.73 | 0.91 | 0.96 | 0.62 | |  |
| *Turdus philomelos* | 0.97 | 0.94 | 0.69 | 0.92 | 0.70 | 0.62 | |  |
| *Turdus torquatus* | 0.99 | 0.98 | 0.92 | 0.97 | 0.99 | 0.80 | |  |
| *Perdix perdix* | 0.99 | 0.98 | 0.92 | 0.94 | 1.00 | 0.70 | |  |
| *Saxicola rubetra* | 0.98 | 0.99 | 0.84 | 0.90 | 0.89 | 0.56 | |  |

| **Table S4.** Wilcoxon pairwise comparisons of the area of habitat between model predictions. Model predictions compared include those from models considering both climate and land cover variables with both allowed to change (CLIMLANDboth), only climate change (CLIMLANDclim) or only land cover change (CLIMLANDland) with respect to the present. Statistic (V) and *P* for the tests are presented. Results for different future scenarios (SSP1-2.6 and SSP2-4.5), using IPSL or MIROC general circulation models, and according to whether suitable habitat extent was calculated across all of Iberia or restricted to current species presences, are shown. Significant results are in bold. N = 32. | | | | | | |
| --- | --- | --- | --- | --- | --- | --- |
| **Scenario** | **Models compared** | | **All Iberia** | | **Restricted** | |
|  |  |  | V | *P* | V | *P* |
| ***IPSL*** |  |  |  |  |  |  |
| SSP1-2.6 | CLIMLANDboth | CLIMLANDland | 0 | **4.7E-10** | 0 | **8.3E-07** |
| SSP2-4.5 | CLIMLANDboth | CLIMLANDland | 2 | **1.4E-09** | 2 | **1.4E-09** |
| SSP1-2.6 | CLIMLANDboth | CLIMLANDclim | 83 | **4.0E-04** | 88 | **0.001** |
| SSP2-4.5 | CLIMLANDboth | CLIMLANDclim | 155 | **0.041** | 141 | **0.037** |
| SSP1-2.6 | CLIMLANDland | CLIMLANDclim | 1 | **9.3E-10** | 1 | **9.3E-10** |
| SSP2-4.5 | CLIMLANDland | CLIMLANDclim | 2 | **1.4E-09** | 2 | **1.4E-09** |
| ***MIROC*** |  |  |  |  |  |  |
| SSP1-2.6 | CLIMLANDboth | CLIMLANDland | 18 | **1.2E-07** | 18 | **1.2E-07** |
| SSP2-4.5 | CLIMLANDboth | CLIMLANDland | 18 | **1.2E-07** | 18 | **1.2E-07** |
| SSP1-2.6 | CLIMLANDboth | CLIMLANDclim | 82 | **3.6E-04** | 95 | **0.002** |
| SSP2-4.5 | CLIMLANDboth | CLIMLANDclim | 154 | **0.039** | 154 | 0.067 |
| SSP1-2.6 | CLIMLANDland | CLIMLANDclim | 22 | **2.5E-07** | 21 | **2.1E-07** |
| SSP2-4.5 | CLIMLANDland | CLIMLANDclim | 19 | **1.4E-07** | 19 | **1.4E-07** |

| **Table S5.** Wilcoxon pairwise comparisons of the area of habitat across future scenarios (SSP1-2.6 and SSP2-4.5), using IPSL or MIROC general circulation models, and current conditions. Statistic (V) and *P* for the tests are presented. Results are shown for different model predictions and for suitable habitat extent calculated across all of Iberia or restricted to current species presences. Significant results are indicated in bold. N = 32. | | | | | | |
| --- | --- | --- | --- | --- | --- | --- |
| **Model** | **Scenarios compared** | | **All Iberia** | | **Restricted** | |
|  |  |  | V | *P* | V | *P* |
| ***IPSL*** |  |  |  |  |  |  |
| CLIMLANDboth | Current | SSP1-2.6 | 527 | **9.3E-10** | 527 | **9.3E-10** |
| CLIMLANDboth | Current | SSP2-4.5 | 527 | **9.3E-10** | 527 | **9.3E-10** |
| CLIMLANDboth | SSP1-2.6 | SSP2-4.5 | 525 | **2.3E-09** | 524 | **1.2E-06** |
| CLIMLANDclim | Current | SSP1-2.6 | 527 | **9.3E-10** | 527 | **9.3E-10** |
| CLIMLANDclim | Current | SSP2-4.5 | 526 | **1.4E-09** | 526 | **1.4E-09** |
| CLIMLANDclim | SSP1-2.6 | SSP2-4.5 | 520 | **1.2E-08** | 521 | **1.6E-06** |
| CLIMLANDland | Current | SSP1-2.6 | 409 | **0.006** | 386 | **0.007** |
| CLIMLANDland | Current | SSP2-4.5 | 428 | **0.002** | 406 | **0.008** |
| CLIMLANDland | SSP1-2.6 | SSP2-4.5 | 132 | **0.012** | 135 | **0.016** |
| ***MIROC*** |  |  |  |  |  |  |
| CLIMLANDboth | Current | SSP1-2.6 | 509 | **1.4E-07** | 510 | **1.2E-07** |
| CLIMLANDboth | Current | SSP2-4.5 | 511 | **9.6E-08** | 513 | **6.4E-08** |
| CLIMLANDboth | SSP1-2.6 | SSP2-4.5 | 526 | **1.4E-09** | 526.5 | **9.6E-07** |
| CLIMLANDclim | Current | SSP1-2.6 | 508 | **1.7E-07** | 509 | **4.8E-06** |
| CLIMLANDclim | Current | SSP2-4.5 | 510 | **1.2E-07** | 510 | **1.2E-07** |
| CLIMLANDclim | SSP1-2.6 | SSP2-4.5 | 522 | **6.5E-09** | 522.5 | **1.4E-06** |
| CLIMLANDland | Current | SSP1-2.6 | 402 | **0.009** | 397.5 | **0.013** |
| CLIMLANDland | Current | SSP2-4.5 | 444 | **4.3E-04** | 405 | **0.002** |
| CLIMLANDland | SSP1-2.6 | SSP2-4.5 | 166 | 0.068 | 171.5 | 0.085 |

| **Table S6.** Wilcoxon pairwise comparisons of the centroid position (longitude and latitude) of suitable habitat **in Iberia** and their distance to the centroid of current suitable habitat across scenarios. Results for scenarios using IPSL or MIROC general circulation models are shown. Statistic (V) and *P* for the tests are presented. Results for different model predictions are shown. Significant results are in bold. N = 32. | | | | | | | | |
| --- | --- | --- | --- | --- | --- | --- | --- | --- |
| **Model** | **Scenarios compared** | | **Longitude** | | **Latitude** | | **Distance** | |
|  |  |  | V | *P* | V | *P* | V | *P* |
| ***IPSL*** |  |  |  |  |  |  |  |  |
| CLIMLANDboth | Current | SSP1-2.6 | 453 | **2.0E-04** | 147 | **0.028** |  |  |
| CLIMLANDboth | Current | SSP2-4.5 | 473 | **2.5E-05** | 137 | **0.017** |  |  |
| CLIMLANDclim | Current | SSP1-2.6 | 470 | **3.5E-05** | 125 | **0.008** |  |  |
| CLIMLANDclim | Current | SSP2-4.5 | 465 | **6.0E-05** | 131 | **0.012** |  |  |
| CLIMLANDland | Current | SSP1-2.6 | 333 | 0.203 | 527 | **9.3E-10** |  |  |
| CLIMLANDland | Current | SSP2-4.5 | 470 | **3.5E-05** | 528 | **4.7E-10** |  |  |
| CLIMLANDboth | SSP1-2.6 | SSP2-4.5 | 493 | **2.0E-06** | 147 | **0.028** | 11 | **2.6E-08** |
| CLIMLANDclim | SSP1-2.6 | SSP2-4.5 | 447 | **3.3E-04** | 234 | 0.586 | 26 | **5.0E-07** |
| CLIMLANDland | SSP1-2.6 | SSP2-4.5 | 262 | 0.978 | 6 | **6.5E-09** | 523 | **4.7E-09** |
| ***MIROC*** |  |  |  |  |  |  |  |  |
| CLIMLANDboth | Current | SSP1-2.6 | 505 | **3.0E-07** | 124 | **0.008** |  |  |
| CLIMLANDboth | Current | SSP2-4.5 | 507 | **2.1E-07** | 123 | **0.007** |  |  |
| CLIMLANDclim | Current | SSP1-2.6 | 516 | **3.3E-08** | 121 | **0.006** |  |  |
| CLIMLANDclim | Current | SSP2-4.5 | 509 | **1.4E-07** | 123 | **0.007** |  |  |
| CLIMLANDland | Current | SSP1-2.6 | 321 | 0.295 | 521 | **8.8E-09** |  |  |
| CLIMLANDland | Current | SSP2-4.5 | 394 | **0.014** | 484 | **6.8E-06** |  |  |
| CLIMLANDboth | SSP1-2.6 | SSP2-4.5 | 463 | **7.4E-05** | 87 | **5.6E-04** | 0 | **4.7E-10** |
| CLIMLANDclim | SSP1-2.6 | SSP2-4.5 | 476 | **1.8E-05** | 159 | **0.050** | 4 | **3.3E-09** |
| CLIMLANDland | SSP1-2.6 | SSP2-4.5 | 224 | 0.465 | 6 | **6.5E-09** | 526 | **1.4E-09** |

| **Table S7.** Wilcoxon pairwise comparisons of the centroid position (longitude and latitude) of suitable habitat and their distance to the centroid of current suitable habitat across models. Results based in all Iberia or restricted to current species presences are shown. Statistic (V) and *P* for the tests are presented. Results for different future scenarios are shown. Significant results are in bold. N = 32. | | | | | | | | | | | | |
| --- | --- | --- | --- | --- | --- | --- | --- | --- | --- | --- | --- | --- |
| Scenario | Models being compared | | | **Longitude** | | | **Latitude** | | | **Distance** | | |
|  |  | |  | V | *P* | | V | | *P* | V | *P* | |
| ***All Iberia*** |  | |  |  |  | |  | |  |  |  | |
| SSP1 (IPSL) | CLIMLANDboth | | CLIMLANDland | 66 | **8.2E-05** | | 399 | | **0.011** | 518 | **2.0E-08** | |
| SSP1 (MIROC) | CLIMLANDboth | | CLIMLANDland | 15 | **6.4E-08** | | 406 | | **0.007** | 523 | **4.7E-09** | |
| SSP2 (IPSL) | CLIMLANDboth | | CLIMLANDland | 64 | **6.7E-05** | | 396 | | **0.012** | 528 | **4.7E-10** | |
| SSP2 (MIROC) | CLIMLANDboth | | CLIMLANDland | 18 | **1.2E-07** | | 405 | | **0.007** | 528 | **4.7E-10** | |
| SSP1 (IPSL) | CLIMLANDboth | | CLIMLANDclim | 252 | 0.832 | | 6 | | **6.5E-09** | 135 | **0.015** | |
| SSP1 (MIROC) | CLIMLANDboth | | CLIMLANDclim | 285 | 0.705 | | 10 | | **2.0E-08** | 86 | **5.1E-04** | |
| SSP2 (IPSL) | CLIMLANDboth | | CLIMLANDclim | 41 | **4.6E-06** | | 4 | | **3.3E-09** | 230 | 0.536 | |
| SSP2 (MIROC) | CLIMLANDboth | | CLIMLANDclim | 190 | 0.172 | | 107 | | **0.003** | 251 | 0.818 | |
| SSP1 (IPSL) | CLIMLANDland | | CLIMLANDclim | 60 | **4.4E-05** | | 408 | | **0.006** | 528 | **4.7E-10** | |
| SSP1 (MIROC) | CLIMLANDland | | CLIMLANDclim | 13 | **4.1E-08** | | 420 | | **0.003** | 528 | **4.7E-10** | |
| SSP2 (IPSL) | CLIMLANDland | | CLIMLANDclim | 75 | **2.0E-04** | | 397 | | **0.012** | 528 | **4.7E-10** | |
| SSP2 (MIROC) | CLIMLANDland | | CLIMLANDclim | 14 | **5.1E-08** | | 405 | | **0.007** | 528 | **4.7E-10** | |
| ***Restricted*** |  | |  |  |  | |  | |  |  |  | |
| SSP1 (IPSL) | CLIMLANDboth | CLIMLANDland | | 309 | | 0.410 | 418 | **0.003** | | 524 | | **3.3E-09** |
| SSP1 (MIROC) | CLIMLANDboth | CLIMLANDland | | 148 | | **0.029** | 475 | **2.0E-05** | | 523 | | **4.7E-09** |
| SSP2 (IPSL) | CLIMLANDboth | CLIMLANDland | | 280 | | 0.775 | 429 | **0.001** | | 528 | | **4.7E-10** |
| SSP2 (MIROC) | CLIMLANDboth | CLIMLANDland | | 148 | | **0.029** | 473 | **2.5E-05** | | 528 | | **4.7E-10** |
| SSP1 (IPSL) | CLIMLANDboth | CLIMLANDclim | | 210 | | 0.322 | 123 | **0.007** | | 314 | | 0.360 |
| SSP1 (MIROC) | CLIMLANDboth | CLIMLANDclim | | 237 | | 0.625 | 129 | **0.011** | | 295 | | 0.573 |
| SSP2 (IPSL) | CLIMLANDboth | CLIMLANDclim | | 326 | | 0.254 | 203 | 0.262 | | 399 | | **0.011** |
| SSP2 (MIROC) | CLIMLANDboth | CLIMLANDclim | | 326 | | 0.254 | 239 | 0.651 | | 359 | | 0.077 |
| SSP1 (IPSL) | CLIMLANDland | CLIMLANDclim | | 296 | | 0.561 | 443 | **4.7E-04** | | 510 | | **1.2E-07** |
| SSP1 (MIROC) | CLIMLANDland | CLIMLANDclim | | 123 | | **0.007** | 479 | **1.3E-05** | | 502 | | **5.0E-07** |
| SSP2 (IPSL) | CLIMLANDland | CLIMLANDclim | | 278 | | 0.803 | 438 | **7.1E-04** | | 525 | | **2.3E-09** |
| SSP2 (MIROC) | CLIMLANDland | CLIMLANDclim | | 112 | | **0.004** | 486 | **5.2E-06** | | 516 | | **3.3E-08** |

| **Table S8.** Wilcoxon pairwise comparisons of the centroid position (longitude and latitude) of suitable habitat within species **current presences** and their distance to the centroid of current suitable habitat across scenarios. Results for scenarios using IPSL or MIROC general circulation models are shown. Statistic (V) and P for the tests are presented. Results for different model predictions are shown. Significant results are in bold. N = 32. | | | | | | | | |
| --- | --- | --- | --- | --- | --- | --- | --- | --- |
| **Model** | **Scenarios being compared** | | **Longitude** | | **Latitude** | | **Distance** | |
|  |  |  | V | *P* | V | *P* | V | *P* |
| ***IPSL*** |  |  |  |  |  |  |  |  |
| CLIMLANDboth | Current | SSP1 (IPSL) | 224 | 0.465 | 152 | **0.036** |  |  |
| CLIMLANDboth | Current | SSP2 (IPSL) | 242 | 0.692 | 107 | **0.003** |  |  |
| CLIMLANDclim | Current | SSP1 (IPSL) | 221 | 0.432 | 107 | **0.003** |  |  |
| CLIMLANDclim | Current | SSP2 (IPSL) | 249 | 0.789 | 101 | **0.002** |  |  |
| CLIMLANDland | Current | SSP1 (IPSL) | 293 | 0.599 | 453 | **2.0E-04** |  |  |
| CLIMLANDland | Current | SSP2 (IPSL) | 216 | 0.379 | 379 | **0.031** |  |  |
| CLIMLANDboth | SSP1 (IPSL) | SSP2 (IPSL) | 296 | 0.561 | 52 | **1.8E-05** | 50 | **1.4E-05** |
| CLIMLANDclim | SSP1 (IPSL) | SSP2 (IPSL) | 336 | 0.184 | 91 | **7.7E-04** | 42 | **5.2E-06** |
| CLIMLANDland | SSP1 (IPSL) | SSP2 (IPSL) | 226 | 0.488 | 97 | **0.001** | 482 | **8.7E-06** |
| ***MIROC*** |  |  |  |  |  |  |  |  |
| CLIMLANDboth | Current | SSP1 (MIROC) | 369 | **0.050** | 61 | **4.9E-05** |  |  |
| CLIMLANDboth | Current | SSP2 (MIROC) | 357 | 0.084 | 59 | **3.9E-05** |  |  |
| CLIMLANDclim | Current | SSP1 (MIROC) | 389 | **0.018** | 50 | **1.4E-05** |  |  |
| CLIMLANDclim | Current | SSP2 (MIROC) | 384 | **0.024** | 50 | **1.4E-05** |  |  |
| CLIMLANDland | Current | SSP1 (MIROC) | 278 | 0.803 | 439 | **6.6E-04** |  |  |
| CLIMLANDland | Current | SSP2 (MIROC) | 219 | 0.410 | 336 | 0.184 |  |  |
| CLIMLANDboth | SSP1 (MIROC) | SSP2 (MIROC) | 354 | 0.095 | 64 | **6.7E-05** | 18 | **1.2E-07** |
| CLIMLANDclim | SSP1 (MIROC) | SSP2 (MIROC) | 399 | **0.011** | 65 | **7.4E-05** | 29 | **8.1E-07** |
| CLIMLANDland | SSP1 (MIROC) | SSP2 (MIROC) | 175 | 0.098 | 113 | **0.004** | 442 | **0.001** |
